# Uprolides N, O and P from the Panamanian Octocoral *Eunicea succinea*

**DOI:** 10.3390/molecules21060819

**Published:** 2016-06-22

**Authors:** Daniel Torres-Mendoza, Yisett González, José Félix Gómez-Reyes, Héctor M. Guzmán, José Luis López-Perez, William H. Gerwick, Patricia L. Fernandez, Marcelino Gutiérrez

**Affiliations:** 1 Center for Biodiversity and Drug Discovery, Institute for Scientific Research and Technology Services (INDICASAT), Clayton, City of Knowledge 0843-01103, Panama; dtorres@indicasat.org.pa (D.T.-M.); gomezjosefelix@gmail.com (J.F.G.-R.); 2 Department of Biotechnology, Acharya Nagarjuna University, Nagarjuna Nagar, Guntur 522510, India; yisettgonzalez@gmail.com; 3 Center for Molecular and Cellular Biology of Diseases, Institute for Scientific Research and Technology Services (INDICASAT), Clayton, City of Knowledge 0843-01103, Panama; pllanes@indicasat.org.pa; 4 Smithsonian Tropical Research Institute, Balboa, Ancon 0843-03092, Panama; guzmanh@si.edu; 5 Department of Pharmaceutical Sciences, Faculty of Pharmacy, University of Salamanca, Ave. Campo Charro, s/n, Salamanca 37007, Spain; lopez@usal.es; 6 Center for Marine Biotechnology and Biomedicine, Scripps Institution of Oceanography and The Skaggs School of Pharmacy and Pharmaceutical Sciences, University of California at San Diego, La Jolla, CA 92037, USA; wgerwick@ucsd.edu

**Keywords:** *Eunicea succinea*, cembranolide diterpenes, uprolides, anti-inflammatory

## Abstract

Three new diterpenes, uprolide N (**1**), uprolide O (**2**), uprolide P (**3**) and a known one, dolabellane (**4**), were isolated from the CH_2_Cl_2_-MeOH extract of the gorgonian octocoral *Eunicea succinea*, collected from Bocas del Toro, on the Caribbean coast of Panama. Their structures were determined using spectroscopic analyses, including 1D and 2D NMR and high-resolution mass spectrometry (HRMS) together with molecular modeling studies. Compounds **1**–**3** displayed anti-inflammatory properties by inhibiting production of Tumor Necrosis Factor (TNF) and Interleukin (IL)-6 induced by lipopolysaccharide (LPS) in murine macrophages.

## 1. Introduction

Gorgonian octocorals are a well-known source of natural products [1,2]. Many of these secondary metabolites are important to the survival strategies of these corals in that they have ecological roles as defensive substances against predators and pathogens and also enhance their ability to compete during reproduction and for space in which to grow [3]. Octocoral metabolites have also proven to be a good resource for drug discovery showing a wide range of pharmacological properties [1,2]. Among octocoral metabolites, diterpenes are a group of compounds frequently found, and these have shown biological activities in different therapeutic areas including antibacterial, antiviral, antifungal, antitumor, anti-arthritic, calcium-antagonistic, anti-inflammatory and antiprotozoal [1,2].

Octocorals from the genus *Eunicea* (family *Plexauridae*) were among the first marine invertebrates subjected to chemical studies [4]. These animals have continued to be a prolific source of chemically complex diterpenoids with diverse architectures, such as the cembradienes, cembrane lactones, dolabellanes, cubitanes, dilophols and fuscols [1]. Furthermore, previous work on *Eunicea* species resulted in the isolation of a group of highly functionalized α-methylene-γ-lactone cembranolides, including a series of compounds that were characterized by the presence of a Δ^6^ olefin known as the uprolides [5,6,7,8,9,10].

As part of our drug discovery program at INDICASAT, we collected several gorgonian octocorals from the Caribbean and Pacific Coasts of Panama. The extracts of these organisms were subsequently evaluated using a series of bioassays including antimicrobial, anticancer, anti-parasitic and anti-inflammatory. Herein we describe the isolation and structural determination of three new compounds, uprolide N (**1**), uprolide O (**2**) and uprolide P (**3**), from the octocoral *Eunicea succinea*, which possessed *in vitro* anti-inflammatory effects by inhibiting the production of TNF and IL-6 in LPS-induced macrophages.

## 2. Results and Discussion

### 2.1. Isolation and Structural Elucidation

**Figure 1:**
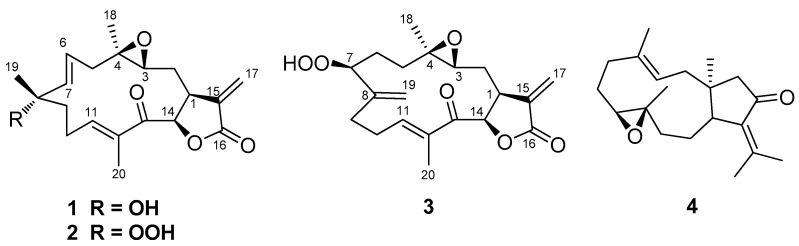
Structures of compounds **1**–**4**.

The octocoral *Eunicea succinea* was collected at −4.5 m by hand using SCUBA in the Isla Bastimentos National Park on the Caribbean coast of Panama. The sample was extracted with a methanol-dichloromethane mixture, and the extract was fractionated using silica gel column chromatography, and subsequently, high performance liquid chromatographic (HPLC) separation to yield compounds **1**–**4** (Figure 1).

The HRESITOF-MS data, collected in positive ion mode for compound **1**, showed a pseudomolecular ion peak [M + H]^+^ at *m*/*z* 347.1871. This mass corresponded with the molecular formula C_20_H_26_O_5_ and was consistent with the carbon and proton count from the ^13^C-NMR and ^1^H-NMR experiments (Table 1, Figures S1 and S2). DEPT and HSQC experiments revealed six quaternary carbons, six methines, five methylenes and three methyl groups (Figures S3 and S5). Their chemical shifts and multiplicities were consistent with an exocyclic olefin at δ_C_ 136.1 (C-15) and 120.0 (CH_2_-17), four sp^3^ carbons bearing oxygen (two at δ_C_ 73.1 (C-8) and 72.5 (CH-14), and two forming an epoxide at δ_C_ 60.9 (C-4) and 56.3 (CH-3)), one ester carbonyl at δ_C_ 169.4 (C-16), and a disubstituted olefin at δ_C_ 140.7 (CH-7) and 124.2 (CH-6). An α,β-unsaturated ketone moiety was implicated by shifts at δ_C_ 195.2 (C-13), 147.8 (CH-11) and 137.2 (C-12). Eight degrees of unsaturation were inferred from the molecular formula: five were accounted for by the two carbonyls and three double bonds. Therefore, compound **1** was deduced to be tricyclic.

**Figure 2:**
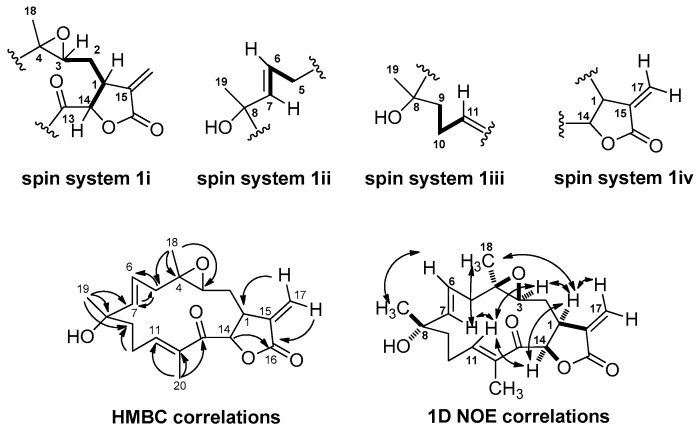
Correlation spectroscopy (COSY), heteronuclear multiple bond correlation (HMBC) and selected 1D nuclear Overhauser effect correlations for compound **1**.

Four spin systems were developed on the basis of COSY experiments (Figure 2 and Figure S4). In spin system **1i**, a deshielded doublet proton at δ_H_ 5.80, attached to an oxygen bearing carbon (C-14, δ_C_ 72.5), was adjacent to a midfield methine (C-1, δ_H_ 3.17, δ_C_ 41.7) with shifts consistent with its allylic nature. In turn, this latter resonance was adjacent to a methylene group (C-2, δ_H_ 1.74, δ_C_ 27.7) that was further coupled to an α-epoxy proton (C-3, δ_H_ 2.65, δ_C_ 56.3). In spin system **1ii**, a doublet vinyl proton (C-7, δ_H_ 5.65, δ_C_ 140.7) was coupled to its olefinic partner (C-6, δ_H_ 5.34, δ_C_ 124.2) with a *J* value of 15.3 Hz, indicating the *trans* nature of this double bond [6,8]. The latter resonance was also coupled to an adjacent methylene group (C-5, δ_H_ 2.58/2.43, δ*_C_* 39.2). The spin system **1iii** was comprised of a significantly deshielded olefin proton (C-11, δ_H_ 6.81, δ_C_ 147.8) that was adjacent to a CH_2_-CH_2_ moiety (C-10, δ_H_ 2.40, δ_C_ 25.3; C-9, δ_H_ 1.97, δ_C_ 41.6). The final spin system **1iv** was comprised of two olefinic protons at δ_H_ 6.28 (d, *J* = 3.3 Hz, H-17a) and 5.44 (d, *J* = 3.3 Hz, H-17b), and were assigned to an exocyclic methylene. In addition, the ^1^H-NMR spectrum for compound **1** showed signals for three methyl groups, two attached to sp^3^ quaternary carbons at δ_H_ 1.37 (s, H_3_-18) and 1.34 (s, H_3_-19) and one attached to a double bond at δ_H_ 1.83 (s, H_3_-20).

The connectivity of these four spin systems with their intervening quaternary carbon atoms and proximate methyl groups was determined using ^2,3^*J* HMBC experiments (Figure 2 and Figure S6). An α-methylene-γ-lactone scaffold was indicated by long range H–C coupling from the exocyclic methylene protons (H_2_-17) to the ester carbonyl at δ_C_ 169.4 (C-16) and methine carbon C-1. A further long range H–C coupling from methine proton H-14 to the same ester carbonyl clarified this bond connection, the 5-membered ring nature of the lactone, and the interconnection of spin systems **1i** and **1iv**. The singlet methyl group at δ_H_ 1.37 (H_3_-18), a shift consistent with its placement on a carbon bearing oxygen, showed HMBC correlations to C-3, C-4 and C-5, thereby connecting partial structures **1i** and **1ii** and defining the epoxide ring. Similarly, the methyl group at δ_H_ 1.34 (H_3_-19), also at a shift consistent with its placement on a carbon bearing oxygen, tied together spin systems **1ii** and **1iii**. Finally, the third singlet methyl group at δ_H_ 1.83 (H_3_-20), a shift consistent with its placement on an olefinic bond, showed HMBC correlations to C-11, C-12 and carbonyl carbon C-13, thereby defining the terminus of spin system **1iii** as belonging to a disubstituted enone system. Because all atoms of **1** were accounted for and placed into a linear array, but required the presence of one additional ring, a final bond connection was made between C-13 and C-14, thereby completing the planar structure of compound **1** as a novel cembrenolide diterpene.

A literature search for related cembrenolides indicated that compound **1** was structurally similar to uproeuniolide, a diterpene isolated from *Eunicea succinea* [7], uprolide K, which was isolated from *Eunicea pinta* [8] and peunicin, isolated from a *Eunicea succinea-mammosa* complex [11]. Comparisons with the spectroscopic data reported for these compounds supported our assignments for compound **1**.

The relative configuration of compound **1** was determined by 1D NOE experiments (Figure 2 and Figures S7–S11), and showed correlations between H-1 and H-3, H-14 and H_3_-18, indicating they were in the same side of the molecule, assigned as the α-plane. Correlations between H-14 and H-11 revealed these latter protons were also in an α-orientation. Moreover, H-7 showed correlations with H_3_-18 and H-11 but not with H-6, confirming their orientation in the α-plane, as well as the *E* geometry of the C-6/C-7 double bond. On the other hand H-6 showed a correlation with H_3_-19, confirming its orientation on the β-plane. Finally, H-11 showed correlations with H-3, H-7 and H-14, but not with H_3_-20, confirming its orientation into the α-plane and the *E*-geometry of the C-11/C-12 double bond.

**Figure 3:**
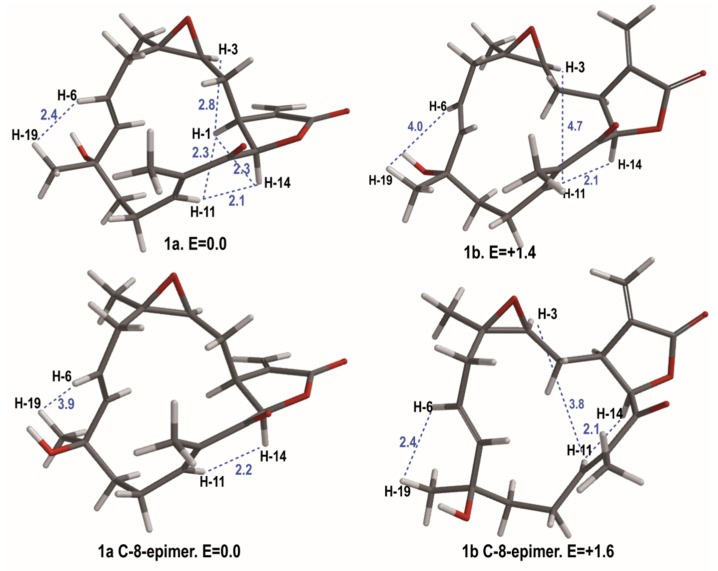
The two lower energy conformers for each C-8 epimer of uprolide N (**1**). Relative energies are expressed in Kcal∙mol^−1^. Selected distances (in blue) are given in Å.

To validate the stereo-configuration of compound **1**, molecular modeling studies were undertaken. A conformational hybrid search method recommended for macrocyclic systems [12], implemented in Macromodel [13], allowed the detection of several conformers for compound **1** (Figure 3). The C-8 epimer was also considered in this study to corroborate the configuration at this carbon atom. The relative stabilities for each conformer were obtained using the MMFF94s force field calculation. To emulate the experimental conditions, the effects of chloroform were considered implicitly during the calculations. The two lower energy conformations for each C-8 epimer of uprolide N (**1**) are represented in Figure 3. The lowest energy conformer **1a** of uprolide N, which is 1.4 kcal mol^-1^ more stable than **1b**, showed a spatial disposition for the Me-19 and C6-C7 double bond very similar to the one of the 3D model of uprolide-A acetate obtained through X-ray diffraction [6]. The calculated inter-proton distances for the lowest energy conformer (**1a**) of uprolide N (Figure 3) agreed with the experimentally determined nOe effects (Figures S7–S11) for this compound, but they disagreed with values for conformer **1b**. In fact, the observations of a strong nOe between H-6 and H-19 and between H-3 and H-11 in compound **1** agree with the distances calculated between both pairs of protons in the lowest energy conformation **1a**. In the case of conformer **1b**, the distances between H-6 and H-19 and between H-3 and H-11 (4.0 and 4.7Å respectively) are too large and do not agree with the strong nOe’s observed between these proton pairs in compound **1**. Consequently, compound **1** should correspond to the configuration and conformation represented in **1a**, reinforcing the assignment of structure (**1**) for this compound.

**Figure 4:**
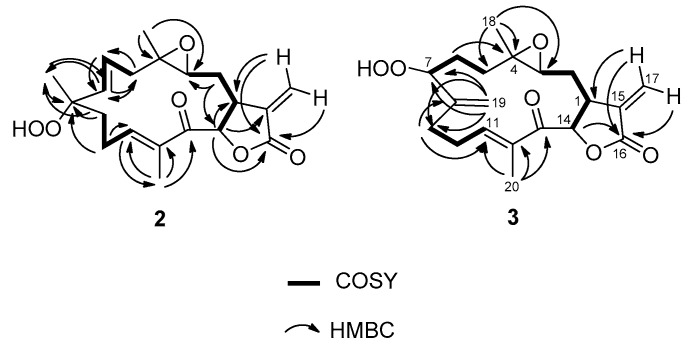
^1^H-^1^H**-**COSY and ^1^H-^13^C-HMBC correlations for compounds **2** and **3**.

The HRESITOF-MS spectra of compound **2** showed a pseudomolecular ion peak [M + Na]^+^ at *m*/*z* 385.1615, consistent with a molecular formula of C_20_H_26_O_6_ which is one O-atom more than compound **1**. This was consistent with the data obtained from the ^13^C- and ^1^H-NMR spectra (Figures S12 and S13), and indicated that compound **2** also had 8 degrees of unsaturation. Five of these were accounted for by two carbonyls (δ_C_ 169.4 (C-16) and 195.2 (C-13)) and three carbon-carbon double bonds (δ_C_ 147.2 (CH-11), 137.5 (C-12), 136.5 (CH-7), 136.1 (C-15), 128.3 (CH-6), 120.0 (CH_2_-17)), indicating that compound **2** was also tricyclic. Indeed, the NMR data for compounds **1** and **2** were highly similar (Table 1), indicating a high degree of structural relatedness (Figure 2 and Figure 4). The major differences between the two compounds were found at C-8 and its surrounding carbon atoms. The presence of an additional oxygen in the molecular formula of compound **2** combined with the chemical shift at C-8, which appeared more deshielded (δ_C_ 84.4) than that in compound **1** (δ_C_ 73.1), was consistent with the presence of a hydroperoxide group at this position (Figure 4) [8].

The relative stereochemistry of **2** was determined by 1D NOE experiments (Figures S18–S23) wherein H-1 correlated with H-14, and H_3_-18 and H-14 correlated with H-1 and H-11, indicating their same relative disposition, assigned to the α-plane. Irradiation on H-7 showed correlations with H-11 but not with H-6. Irradiation on H-6 showed no correlation with H-7 confirming the *trans* orientation of this double bond. A correlation between H-6 and H_3_-19 indicated the β-orientation of these protons.

The HRESITOF-MS spectra of compound **3** showed a pseudomolecular [M + Na]^+^ ion at *m*/*z* 385.1620; this was consistent with the molecular formula C_20_H_26_O_6_. As in compounds **1** and **2**, eight degrees of unsaturation were deduced from the molecular formula; five were accounted for by two carbonyls and three double bonds, indicating compound **3** also had three rings. The ^1^H-NMR data for compound **3** were highly comparable to compounds **1** and **2**, except in the region of C-5 to C-9 and C-19 (Table 1, Figures S24 and S25). The differences were that a new exocyclic olefin was present at C-8 to C-19 (δ_H_ 5.25 (s, H-19a), 5.15 (s, H-19b); δ_C_ 145.7 (C-8), 113.7 (CH_2_-19)), and a new oxygen-bearing carbon appeared at δ_C_ 87.3, C-7 (δ_H_ 4.28, br t, *J* = 6.9 Hz, H-7). By COSY and ^2,3^*J* HMBC experiments (Figure 4, Figures S27 and S29), this latter resonance could be connected to higher field methylene groups at C-6 (δ_H_ 1.84 and 1.61; δ_C_ 30.8) and C-5 (δ_H_ 1.24; δ_C_ 31.9). The location of oxidation was further revealed by a ^3^*J* HMBC correlation observed for H_2_-19 to C-7. Considering the molecular formula of **3** which showed one more O-atom than compound **1**, and the relatively deshielded shift for C-7 (δ_C_ 87.3) [8], it became clear that compound **3** was the alternate allylic oxidation product from a presumed biosynthetic precursor containing a C-7-C-8 double bond such as peunicin [11]. The structure of compound **3** resembles that of uprolide C, previously described from *Eunicea mammosa* [6,8]. Comparisons with the spectroscopic data for this latter compound supported our assignments for compound **3**.

The relative stereochemistry of compound **3** was confirmed by 1D NOE experiments (Figures S30–S36). Irradiation of H-1 showed correlations with, H-14 and H_3_-18, indicating that they are all on the same α-plane. Also, correlations observed between H-14 and H-11 and between H-11 and H-7 indicated these protons were α-oriented, and therefore, the hydroperoxy group on C-7 must be β-orientated.

To date, more than 30 uprolide diterpenes have been isolated from several *Eunicea* species. This family is characterized by the presence of an α-methylene-γ-lactone moiety attached to a highly functionalized 14-membered carbocycle which forms the cembranolide scaffold. The major differences among the uprolides are the stereochemical assignments of C-1, C-8, C-12, C-13 and C-14, the degree of oxidation at C-13, and the presence of a hydroxyl group, a hydroperoxy group or an exocyclic double bond at C-8. Additionally, uprolides D-G possess an unusual 4,7-oxa-bridge functionality.

### 2.2. Anti-inflammatory Effect

Inflammation is the biological response of a host towards harmful stimuli, such as pathogens, physical injury or damaged cells. The inflammatory response initiates with the recruitment of immune cells to injured areas. These cells produce inflammatory mediators including cytokines, chemokines and reactive nitrogen and oxygen species. These mediators are critical for resolving inflammation and for tissue repair; however they can also cause tissue damage. Immune cells, such as macrophages, are equipped with various pattern recognition receptors (PRRs) that recognize many structurally different molecules, including lipopolysaccharide (LPS), lipoproteins, lipids, nucleic acids and proteins [14]. The activation of macrophages by LPS leads to the production of high levels of pro-inflammatory cytokines such as TNF-α and IL-6.

**Figure 5:**
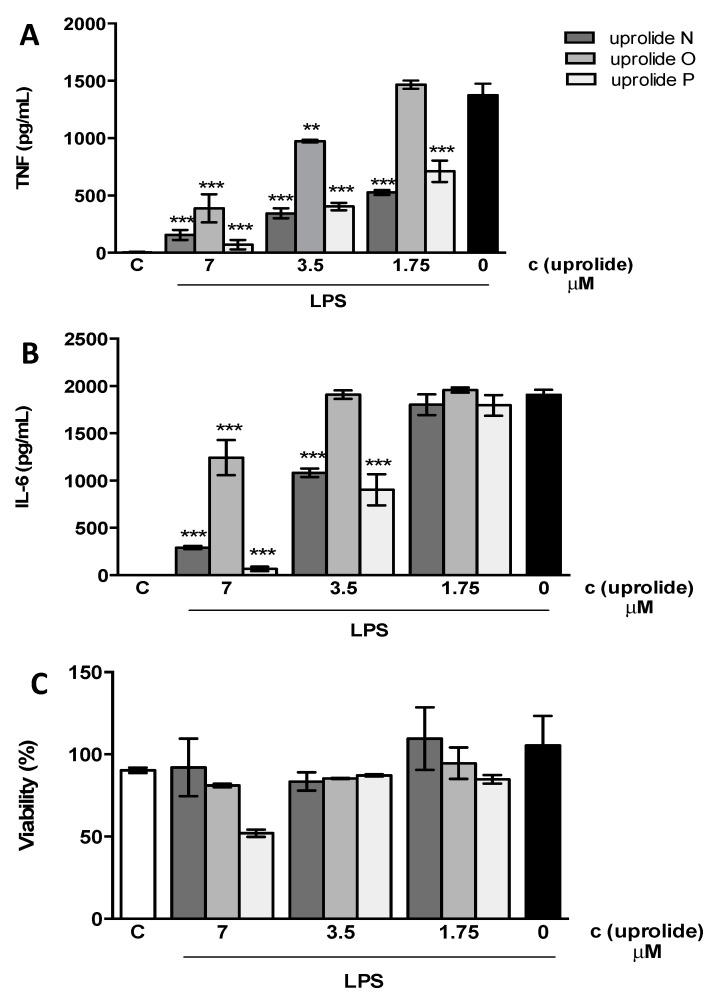
Uprolides compounds inhibit the production of TNF (**A**) and IL-6 (**B**) induced by LPS in macrophages. Peritoneal macrophages from C57B1/6 mice were treated with different concentrations of compounds **1**, **2** or **3** (uprolides N, O or P) 1 h before stimulation with 100 μg/mL of LPS. The supernatants were harvested after 6 h, and cytokine concentrations were determined by ELISA. Results are represented as Mean ± S.E.M. from two independent experiments performed in duplicate. **, *p* < 0.01, ***, *p* < 0.001 relative to the LPS stimulus alone. (**C**) The cell viability was assessed using a MTT assay after collecting the supernatant. Results are represented as Mean ± S.E.M. from stimuli performed in duplicate and are representative of two different experiments. C represents the control plus 0.5% of DMSO without the stimulus.

Inhibition of secretion of pro-inflammatory cytokines is frequently used to indicate anti-inflammatory activity. Herein, we evaluated the ability of compounds **1**–**3** to inhibit the production of TNF-α and IL-6 in primary murine macrophages stimulated with LPS. Compounds **1**–**3** inhibited the production of TNF and IL-6 in a dose-dependent manner (Figure 5A,B). IC_50_ values for both mediators are presented in Table 2 (also in Figure S46). The effect of these compounds was not attributed to cellular death because cell viability was not affected at the inhibitory concentrations (Figure 5C and Table 2). However, 60%–70% of cell death was observed after the exposure of cells to 14 μM concentrations of compounds **1** and **3** (data not shown). A cytotoxic effect on cancer cell lines has been demonstrated for other uprolides [15]. In the absence of LPS compounds were unable to induce the production of TNF and IL-6 (Figure S47).

The anti-inflammatory activity of certain families of cembranoids has been attributed to the presence of the α-methylene-γ-lactone group [16], a feature that is also present in compounds **1**–**3**. Although the lactone group is commonly present in other uprolides previously described, the anti-inflammatory activity of these compounds was not evaluated. This is the first report showing this effect and thus identifies the uprolides as possessing anti-inflammatory properties; however, further studies are necessary to characterize the mechanism of action of these compounds. Compound **4** did not exhibit inhibitory effect on the activation of macrophages by LPS (Table 2).

## 3. Experimental Section

### 3.1. General Procedures

Optical rotations were measured in CHCl_3_ using either a P-2000 digital polarimeter (JASCO, Easton, MD, USA) or an Autopol^®^ III automatic polarimeter (Rudolph, Hackettstown, NJ, USA). UV spectra were recorded using a model UV-2401 (PC) UV-Vis spectrophotometer (Shimadzu, Columbia, MD, USA). IR spectra were recorded using a Platinum ATR ALPHA instrument (Bruker, Billerica, MA, USA). The ^1^H-, ^13^C- and 2D-NMR spectra were recorded on a Eclipse 400 MHz spectrometer (JEOL, Peabody, MA, USA). The chemical shifts were calibrated internally based on the signal for the residual solvent in which the sample was dissolved (CDCl_3_, δ_H_ 7.26, δ_C_ 77.0). Accurate mass APCI spectra were acquired on a JEOL LC-mate mass spectrometer (Peabody, MA, USA) and HRESITOF-MS were acquired on a microTOF-QIII spectrometer (Bruker Daltonics, Billerica, MA, USA). HPLC purification was carried out using an 1100 HPLC system (Agilent, Santa Clara, CA, USA) equipped with a quaternary pump, an Agilent diode array detector 1200 Series and a normal phase silica gel column (Sphereclone, 250 mm × 10.0 mm, 5 μm, Phenomenex^®^, Torrance, CA, USA). The flash chromatographic separations were performed using grade 60 silica gel (70–230 mesh, 60 Å, Sigma-Aldrich, St. Louis, MO, USA) and silica gel (Sigma-Aldrich, St. Louis, MO, USA, grade 9385, 230–400 mesh, 60 Å, Sigma). Merck TLC sheets (silica gel 60 F_254_) were used to perform analytical TLC (aluminum-supported, layer-thickness 200 μm).

### 3.2. Animal Material

One specimen of the octocoral *Eunicea succinea* (Order Gorgonacea, Family Plexauridae) was collected at −4.5 m by hand using SCUBA in the Bastimentos National Park, which is located in the Caribbean off the coast of Bocas del Toro, Panama, in November 2009. The coral specimen was identified as *Eunicea succinea* (Pallas 1766) based on its morphology. A reference specimen is deposited at INDICASAT’s CBDD under the number GLBO-241109-02.

### 3.3. Extraction and Isolation

The organism (442 g) was minced and exhaustively extracted at room temperature with 1 × 400 mL of CH_2_Cl_2_ and then with 6 × 400 mL of 1:1 CH_2_Cl_2_/MeOH. The organic extracts were combined and evaporated in vacuo to yield a dark-green oily residue (16.0 g). The CH_2_Cl_2_–MeOH extract was fractionated using column chromatography on silica gel with a stepwise gradient of 0%–100 % of EtOAc in hexanes followed by 0%–100% of MeOH in EtOAc to yield 10 fractions (A–J). Fraction D (2.4 g) was chromatographed on silica gel with a stepwise gradient of CH_2_Cl_2_-acetone (100:0, 98:2, 90:10, 80:20, 0:100) to obtain 150 fractions collected in 18-mL vials. The fractions were analyzed and combined based on their TLC profile to yield 13 fractions (D1–D13). Fraction D10 (136.7 mg) was purified by HPLC (normal phase, gradient from 20% to 100% of EtOAc in hexanes at 1 mL/min, for 75 min, followed by 5 min of 100% EtOAc) to yield uprolides O (**2**) (1.3 mg, t_R_ 53.6 min) and N (**1**) (1.3 mg, t_R_ 76.4 min). Fraction D7 (647.1 mg) was fractionated using column chromatography on silica gel with a gradient of CH_2_Cl_2_–acetone (100:0, 97:3, 95:5, 91:9, 80:20, 0:100) to yield 12 subfractions (I-XII). Subfraction III was purified by HPLC (normal phase, gradient from 0% to 100% of EtOAc in hexanes at 1 mL/min, in 120 min) to yield dolabellane **4** (1.3 mg, t_R_ 48.1 min). Fraction E (300 mg) was purified by HPLC (normal phase, isocratic gradient 46:54% Hx-EtOAc, at 1 mL/min) to yield uprolide O (7.6 mg, t_R_ 35 min) and uprolide P (**3**) (5.0 mg, t_R_ 31.8 min).

### 3.4. Compound Characterization

*Uprolide N* (**1**): colorless glassy solid; ${\left[\alpha \right]}_{D}^{25}$ −26.0° (0.8 *c*, CHCl_3_); UV (MeOH) *λ*_max_ 241 nm (ε 7711); IR 3533, 3093, 1764, 1664, 1377, 1262 cm^−1^; for ^1^H- and ^13^C-NMR data see Table 1; HRESITOF-MS *m*/*z* 347.1871 [M + H]^+^ (calcd for C_20_H_27_O_5_, 347.1853).

*Uprolide O* (**2**): colorless glassy solid; ${\left[\alpha \right]}_{D}^{25}$ −23.3° (0.45 *c*, CHCl_3_); UV (MeOH) *λ*_max_ 243 nm (ε 9926); IR 3510, 3127, 1766, 1664, 1461, 1377, 1251 cm^−1^; for ^1^H- and ^13^C-NMR data see Table 1; HRESITOF-MS *m*/*z* 385.1615 [M + Na]^+^ (calcd for C_20_H_26_O_6_Na, 385.1622).

*Uprolide P* (**3**): colorless glassy solid; ${\left[\alpha \right]}_{D}^{25}$ −32.6° (0.31 *c*, CHCl_3_); UV (MeOH) *λ*_max_ 242 nm (ε 10014); IR 3514, 3162, 1763, 1666, 1457, 1377, 1263 cm^−1^; for ^1^H- and ^13^C-NMR data see Table 1; HRESITOF-MS *m*/*z* 385.1620 [M + Na]^+^ (calcd for C_20_H_26_O_6_Na, 385.1622).

*Dolabellane* (**4**) [17]: colorless glassy solid, ^1^H-NMR (CDCl_3_, 400 MHz) 5.42 (1H, dd, *J* = 11.6. 11.8 Hz, H-3), 2.91 (1H, d, *J* = 12.6 Hz, H-7), 2.68 (1H, d, *J* = 12.6, H-11), 2.40 (1H, br d, *J* = 12.6, H-14), 2.26 (3H, s, H_3_-20), 2.16 (1H, dd, *J* = 11.6, 11.8, H-2), 2.12 (1H, d, *J* = 18.6, H-14), 2.04 (1H, br m, H-5), 1.93 (3H, s, H_3_-19), 1.68 (1H, m, H-6), 1.63 (1H, dd, *J* = 11.8, 4.7, H-2), 1.57 (3H, s, H_3_-16), 1.50 (1H, m, H-6), 1.38 (1H, m, H-5), 1.36 (3H, s, H_3_-17), 1.19 (3H, s, H_3_-15) . ^13^C-NMR (CDCl_3_, 100 MHz) 206.5 (C=O, C-13), 149.2 (C, C-18), 13 7.7 (C, C-4), 136.0 (C, C-12), 124.7 (CH, C-3), 65.8 (CH, C-7), 60.5 (C, C-8), 54.4 (CH_2_, C-14), 42.1 (CH, C-11), 41.1 (C, C-1), 39.8 (CH_2_, C-2), 38.0 (CH_2_, C-9), 36.9 (CH_2_, C-5), 27.5 (CH_2_, C-6), 25.0 (CH_3_, C-19), 23.4 (CH_3_, C-15), 22.9 (CH_2_, C-10), 21.8 (CH_3_, C-20), 17.7 (CH_3_, C-17), 15.7 (CH_3_, C-16); APCI-MS *m*/*z* 303.25 [M + H]^+^; -LRAPCI-MS *m*/*z* 303.2255 [M + H]^+^ (calcd for C_20_H_31_O_2_, 303.2324).

### 3.5. Molecular Modeling Studies

A conformational search was performed using the default protocol for macrocycle sampling in Macromodel. This method uses a hybrid search method that employs 5000 cycles of MD-simulated annealing followed by 5000 cycles of LLMOD (large-scale low mode) search steps. The simulated annealing stage produces a set of nonredundant candidate structures that are used as seeds for the conformational search. The torsion sampling option called “enhanced” was selected. Each conformation was minimized using a MMFF94 force field, and no energy cutoff was applied to discard unreasonable conformations. The structural drawings were produced in Spartan08 (Wavefunction, Inc., Irvine, CA, USA).

### 3.6. Cell Culture and Cytokines Determination

The peritoneal macrophages from C57B1/6 mice were obtained five days after an i.p. instillation of 2 mL of thioglycollate 3%, through peritoneal washing with chilled RPMI. The cells were seeded in RPMI with 10% FCS at 2 × 10^5^ cells/well in 96-well plates. The plates were incubated for 2 h at 37 °C under 5% CO_2_ atmosphere. The non-adherent cells were removed by washing, and the adherent cells were stimulated with 100 ng/mL of LPS with or without different concentrations of compounds **1**, **2** or **3** (14, 7, 3.5 or 1.75 μM). The supernatants were collected 6 h after the stimulus, and the concentrations of TNF and IL-6 were determined by ELISA (DuoSet kit, R & D System, Inc. Minneapolis, MN, USA), according to the manufacturer′s protocol.

### 3.7. Cytotoxicity Assay

After the removing the supernatants, 100 μL of MTT (Sigma) (0.5 mg/mL) in RPMI was added to each well, and the cells were incubated overnight at 37 °C. The MTT is a water soluble tetrazolium salt, which is reduced by the activity of succinate dehydrogenase of living cells mitochondria to an insoluble purple product (formazan crystals). The supernatants were removed, and the formazan crystals were dissolved in 100 μL of 0.04 M HCl in isopropanol. The color was analyzed at 570 nm on an ELISA plate reader. The percentage of viable cells was calculated as % viability = (OD sample/OD control) × 100%. The non-stimulated cells cultured in medium plus 10% FCS represent the control. As compounds were diluted in DMSO, we determined the sensitivity of macrophages to several concentrations of this diluent (2%, 1%, 0.5%, 0.25% and 0.125%). Only concentrations above 2% showed significant toxicity. Therefore, we performed the rest of the experiments in the presence of 0.5% DMSO. At this concentration the cell viability was 99.1% ± 9.0% (mean ± SD).

### 3.8. Statistical Analysis

The results were analyzed using the statistical software package GraphPad Prism 5 (GraphPad Software, La Jolla, CA, USA). The statistical analysis was performed using a one-way ANOVA followed by Dunnet’s multiple comparisons post-test. The differences between groups were considered significant when *p* < 0.05. The 50% inhibitory concentration (IC_50_) was calculated by adjusting a sigmoidal dose-response curve following the standard procedure in GraphPad Prism 5.

## 4. Conclusions

In this work, we isolated three new uprolides N-P (**1**–**3**); these compounds are the first members of this family that have shown anti-inflammatory activity, indicating that this group of compounds should be further explored as a source of new anti-inflammatory lead compounds.

## Figures and Tables

**Table 1:** ^1^H- and ^13^C-NMR data in ppm for uprolides N, O and P (**1**–**3**) measured in CDCl_3_ at 400 MHz.

Position	Uprolide N (1)		Uprolide O (2)		Uprolide P (3)	
	δ_C_	δ_H_ (*J* in Hz)	δ_C_	δ_H_ (*J* in Hz)	δ_C_	δ_H_ (*J* in Hz)
**1**	41.7, CH	3.17, m	41.6, CH	3.17, m	40.8, CH	3.26, m
**2**	27.7,CH_2_	1.74, m	27.6, CH_2_	1.74, m	29.1, CH_2_	1.95, m 1.69, m
**3**	56.3, CH	2.65, dd (4.0, 9.9)	56.2, CH	2.72, m	56.6, CH	2.47, dd (4.7, 9.5)
**4**	60.9, C		60.8, C		59.2, C	
**5a** **5b**	39.2, CH_2_	2.58, m 2.43, m	39.1, CH_2_	2.51, m 2.65, m	31.9, CH_2_	1.24, m
**6**	124.2, CH	5.34, ddd (4.7, 9.8, 15.3)	128.3, CH	5.45, ddd (4.3, 10.2, 16.5)	30.8, CH_2_	1.84, m 1.61, m
**7**	140.7, CH	5.65, d (15.3)	136.5, CH	5.63, d (16.5)	87.3, CH	4.28, br t (6.9)
**8**	73.1, C		84.4, C		145.7, C	
**9**	41.6, CH_2_	1.97, m	36.9, CH_2_	2.01, m	30.2, CH_2_	2.40, m 2.34, m
**10**	25.3, CH_2_	2.40, m	24.8, CH_2_	2.45, m	28.3, CH_2_	2.72, m 2.51, m
**11**	147.8, CH	6.81, br t (6.6)	147.2, CH	6.76, br t (5.9)	145.4, CH	6.91 br dd (4.8, 9.1)
**12**	137.2, C		137.5, C		138.0, C	
**13**	195.2, C		195.2, C		195.5, C	
**14**	72.5, CH	5.80, br d (7.3)	72.9, CH	5.80, d (8.0)	73.3, CH	5.82, d (8.0)
**15**	136.1, C		136.1, C		136.8, C	
**16**	169.4, C		169.4, C		169.3, C	
**17a** **17b**	120.0, CH_2_	6.28, d (3.3) 5.44, d (3.3)	120.0, CH_2_	6.28, d (3.3) 5.44, d (3.3)	120.8, CH_2_	6.28, d (2.2) 5.47, d (2.2)
**18**	18.2, CH_3_	1.37, s	18.2, CH_3_	1.36, s	18.0, CH_3_	1.32, s
**19a** **19b**	25.9, CH_3_	1.34, s	19.5, CH_3_	1.35, s	113.7, CH_2_	5.25, s 5.15, s
**20**	11.4, CH_3_	1.83, s	11.3, CH_3_	1.82, s	11.2, CH_3_	1.86, s

**Table 2:** Anti-inflammatory activity of the new uprolides N-P (**1**–**3**) and dolabellane (**4**).

Mediator	IC_50_ ± S.D. (μM)			
	Uprolide N (1)	Uprolide O (2)	Uprolide P (3)	Dolabellane (4)
**TNF**	1.39 ± 0.48	2.73 ± 0.03	2.27 ±0.91	n.a.
**IL-6**	3.26 ± 0.32	4.22 ± 1.52	2.60 ±1.47	n.a.

Values represent average of IC_50_ from four independent experiments ± S.D. n.a. non-active.

## Supplementary Materials

NMR, MS and IR spectra for uprolides N (**1**), O (**2**) and P (**3**). The following are available online at http://www.mdpi.com/1420-3049/21/6/819/s1.
