# Supplementary material for: Uprolides N, O and P from the Panamanian Octocoral Eunicea succinea
Source: Molecules. 2016 Jun 22;21(6):819. doi: 10.3390/molecules21060819 (PMC6273691; doi:10.3390/molecules21060819)
Supplement: Supplementary file 1 [file molecules-21-00819-s001.pdf]

Supplementary Materials: Uprolides N, O and P from the Panamanian Gorgonian Octocoral *Eunicea succinea*

Daniel Torres-Mendoza, Yisett González, José Félix Gómez-Reyes, Héctor M. Guzmán, José Luis López-Perez, William H. Gerwick, Patricia L. Fernandez and Marcelino Gutiérrez

Figure S1. Uprolide N, ^{1}H-NMR spectrum
Figure S2. Uprolide N, ^{13}C-NMR spectrum
Figure S3. Uprolide N, ^{13}C-NMR-DEPT 135 spectrum
Figure S4. Uprolide N, ^{1}H-^{1}H-COSY spectrum
Figure S5. Uprolide N, HSQC spectrum
Figure S6. Uprolide N, HMBC spectrum
Figure S7. Uprolide N, 1D NOE irradiation on H-1
Figure S8. Uprolide N, 1D NOE irradiation on H-6
Figure S9. Uprolide N, 1D NOE irradiation on H-7
Figure S10. Uprolide N, 1D NOE irradiation on H-11
Figure S11. Uprolide N, 1D NOE irradiation on H-14
Figure S12. Uprolide O, ^{1}H-NMR spectrum
Figure S13. Uprolide O, ^{13}C-NMR spectrum
Figure S14. Uprolide O, ^{13}C-NMR-DEPT 135 spectrum
Figure S15. Uprolide O, ^{1}H-^{1}H-COSY spectrum
Figure S16. Uprolide O, HSQC spectrum
Figure S17. Uprolide O, HMBC spectrum
Figure S18. Uprolide O, 1D NOE irradiation on H-1
Figure S19. Uprolide O, 1D NOE irradiation on H-6/H-17
Figure S20. Uprolide O, 1D NOE irradiation on H-7
Figure S21. Uprolide O, 1D NOE irradiation on H-11
Figure S22. Uprolide O, 1D NOE irradiation on H-14
Figure S23. Uprolide O, 1D NOE irradiation on H-17
Figure S24. Uprolide P, ^{1}H-NMR spectrum
Figure S25. Uprolide P, ^{13}C-NMR spectrum
Figure S26. Uprolide P, ^{13}C-NMR DEPT 135 spectrum
Figure S27. Uprolide P, ^{1}H-^{1}H-COSY spectrum
Figure S28. Uprolide P, HSQC spectrum
Figure S29. Uprolide P, HMBC spectrum

Figure S30. Uprolide P, 1D NOE irradiation on H-1
Figure S31. Uprolide P, 1D NOE irradiation on H-7
Figure S32. Uprolide P, 1D NOE irradiation on H-11
Figure S33. Uprolide P, 1D NOE irradiation on H-14
Figure S34. Uprolide P, 1D NOE irradiation on H-19a
Figure S35. Uprolide P, 1D NOE irradiation on H-19b
Figure S36. Uprolide P, 1D NOE irradiation on H-20
Figure S37. Uprolide N, HRESITOF-MS
Figure S38. Uprolide O, ESI-TOF-MS
Figure S39. Uprolide P, ESI-TOF-MS
Figure S40. Uprolide N IR spectrum
Figure S41. Uprolide O IR spectrum
Figure S42. Uprolide P IR spectrum
Figure S43. Dolabellane, ^{1}H-NMR spectrum
Figure S44. Dolabellane, ^{13}C-NMR spectrum
Figure S45. Dolabellane, LRAPCI-MS
Figure S46. Uprolides N, O and P inhibit the production of inflammatory mediators induced by LPS in murine macrophages
Figure S47: Compounds N and P do not induced the production of TNF and IL-6

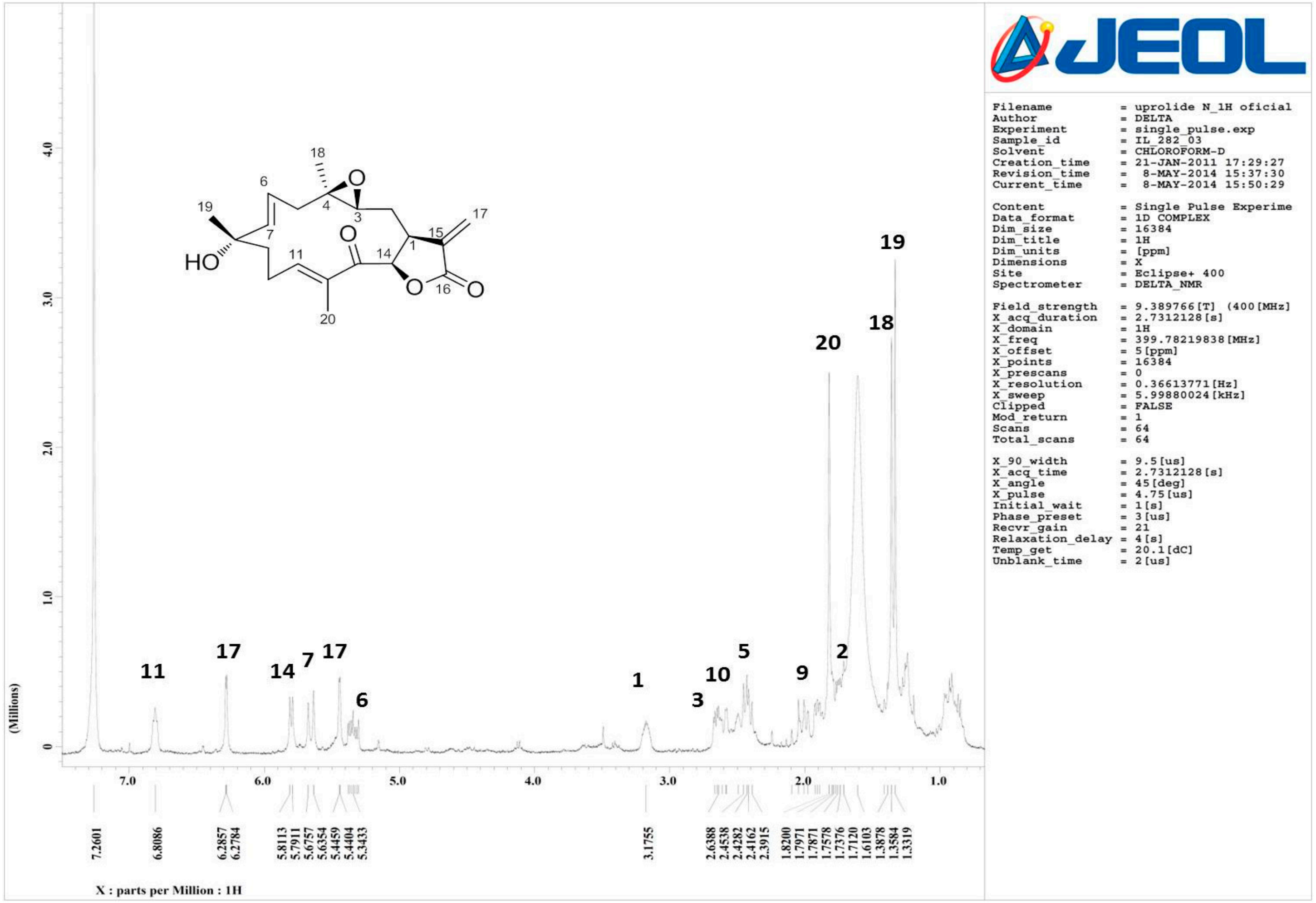

Figure S1. Uprolide N, ¹H-NMR spectrum.

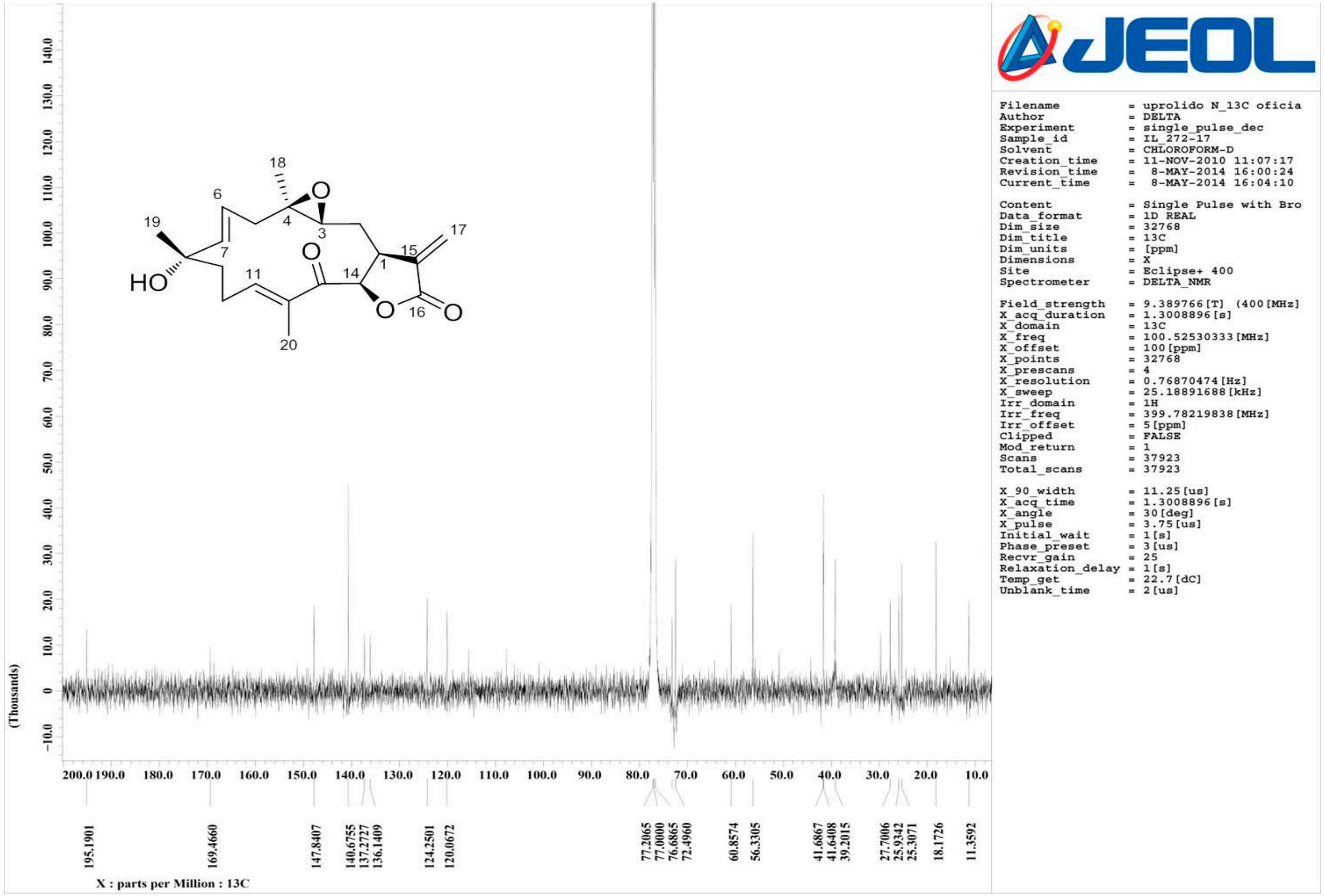

Figure S2. Uprolide N, ^{13}C-NMR spectrum.

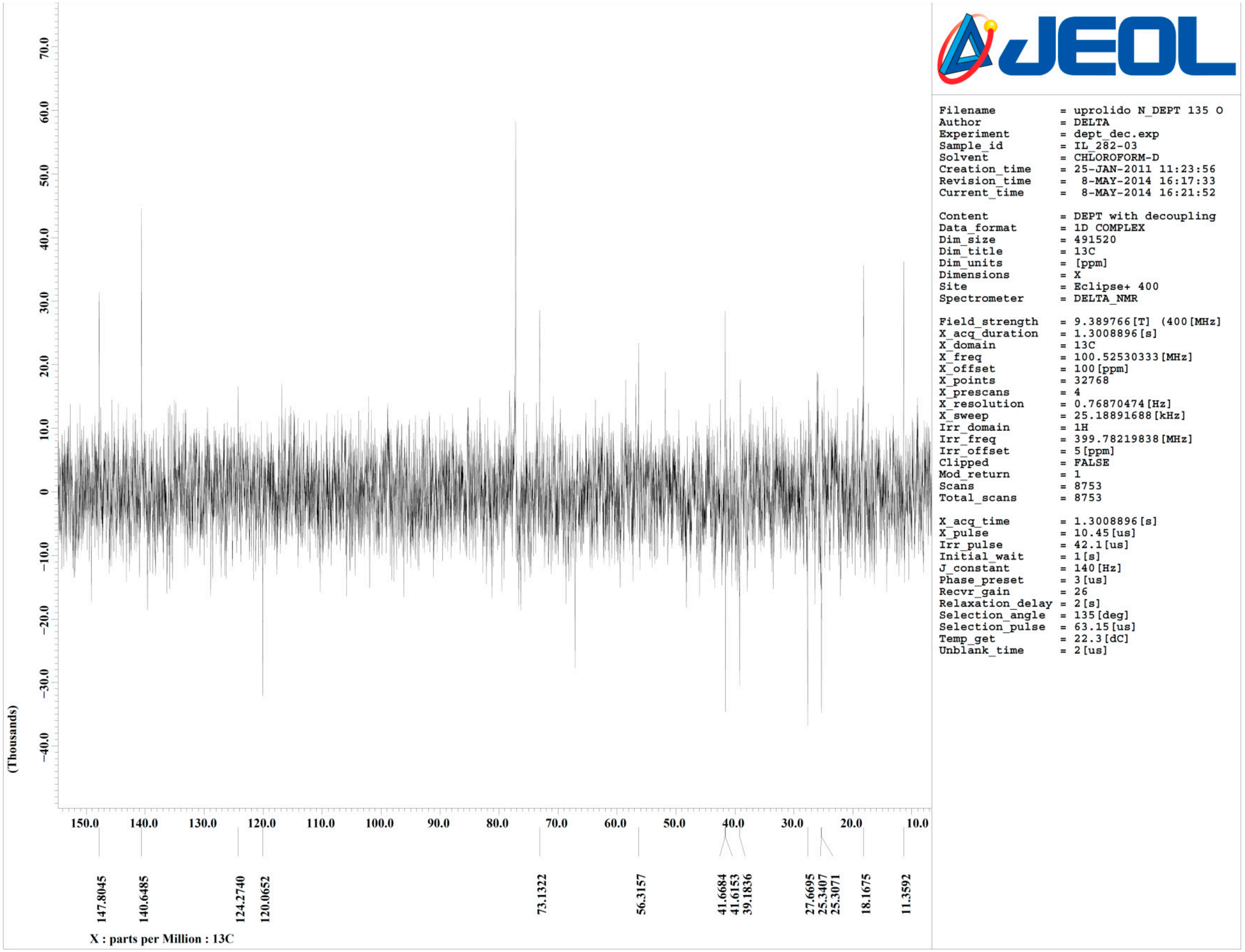

Figure S3. Uprolide N, ^{13}C-NMR-DEPT 135 spectrum.

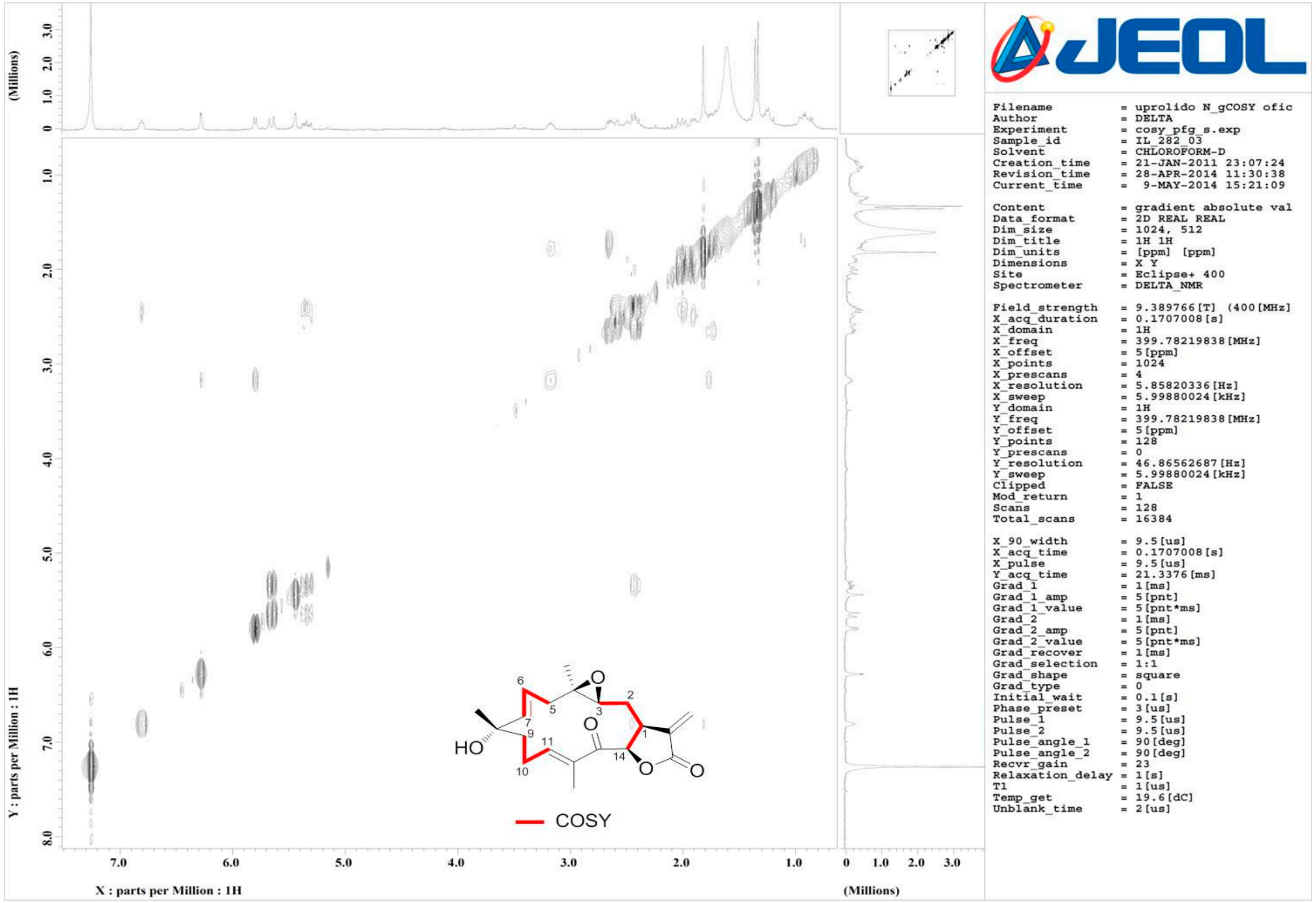

Figure S4. Uprolide N, ^{1}H-^{1}H-COSY spectrum.

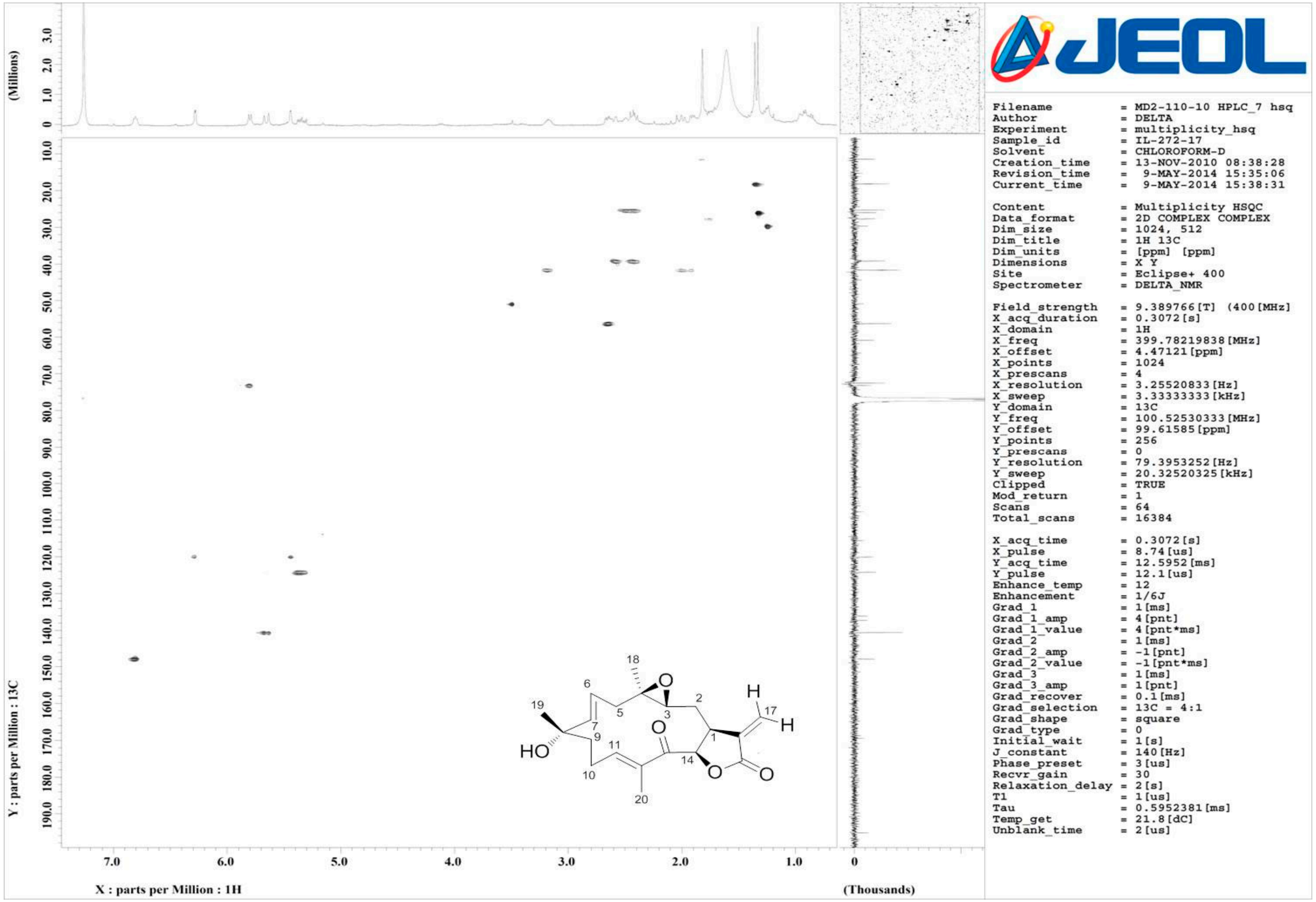

Figure S5. Uprolide N, HSQC spectrum.

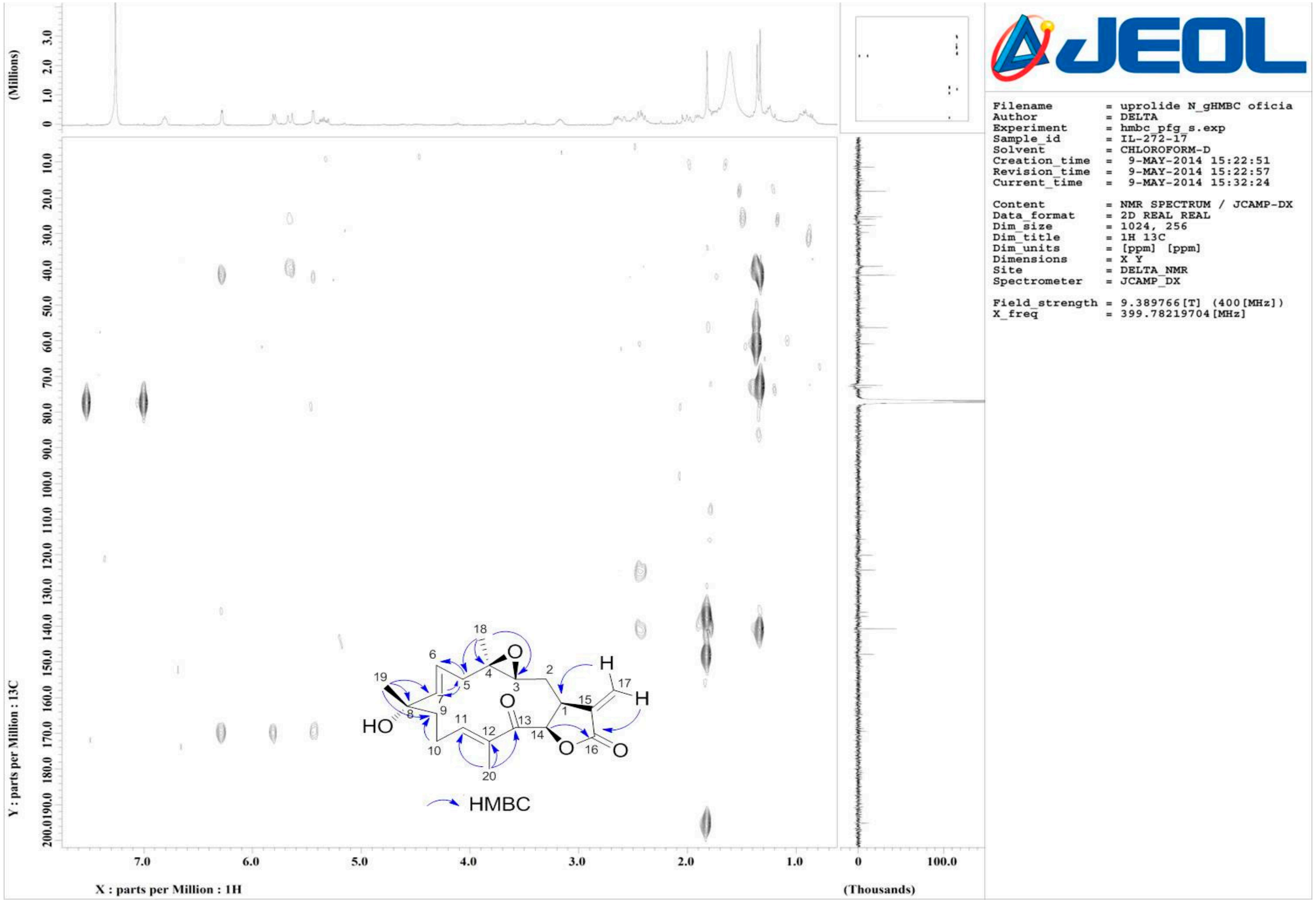

Figure S6. Uprolide N, HMBC spectrum.

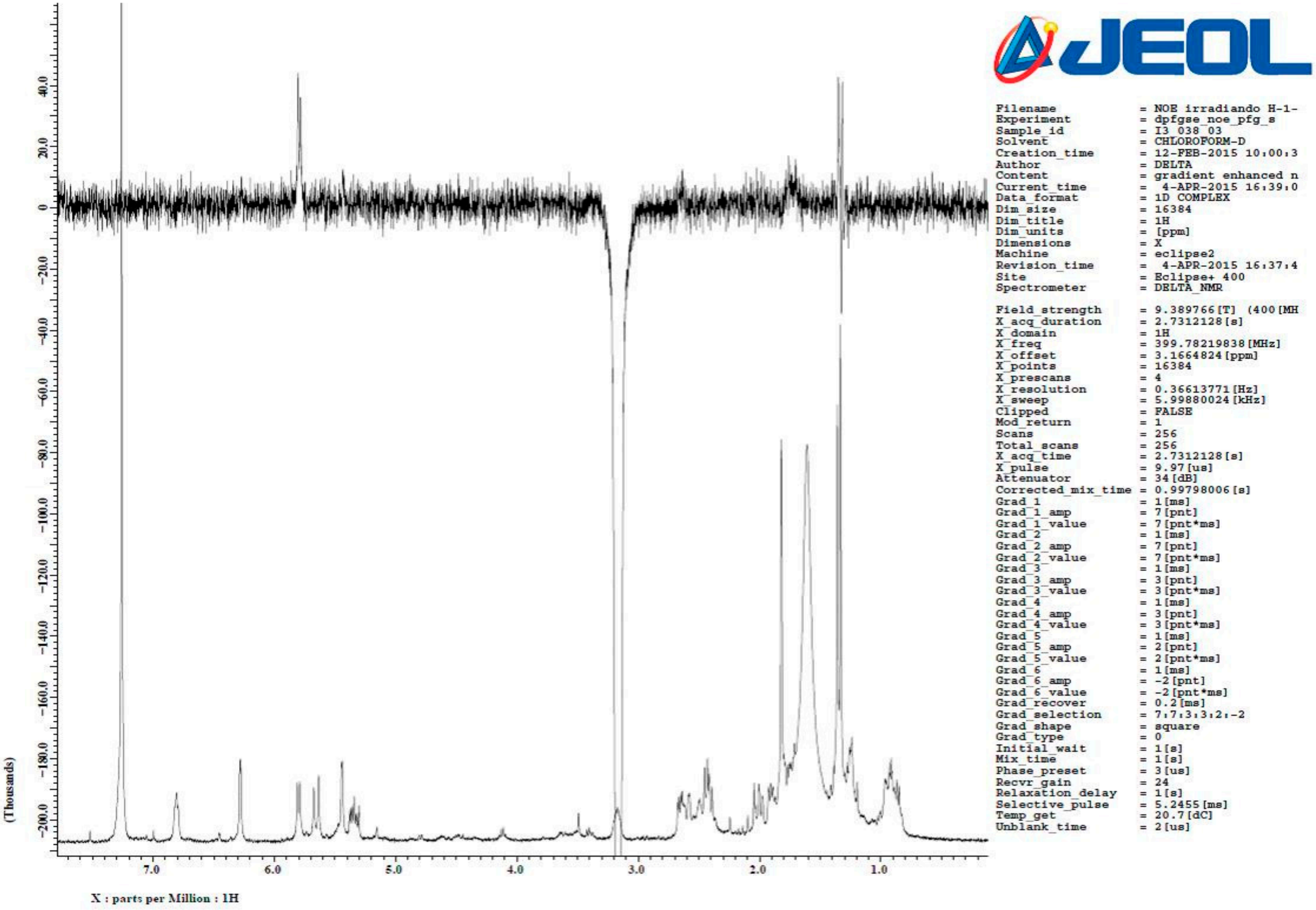

Figure S7. Uprolide N, 1D NOE irradiation on H-1.

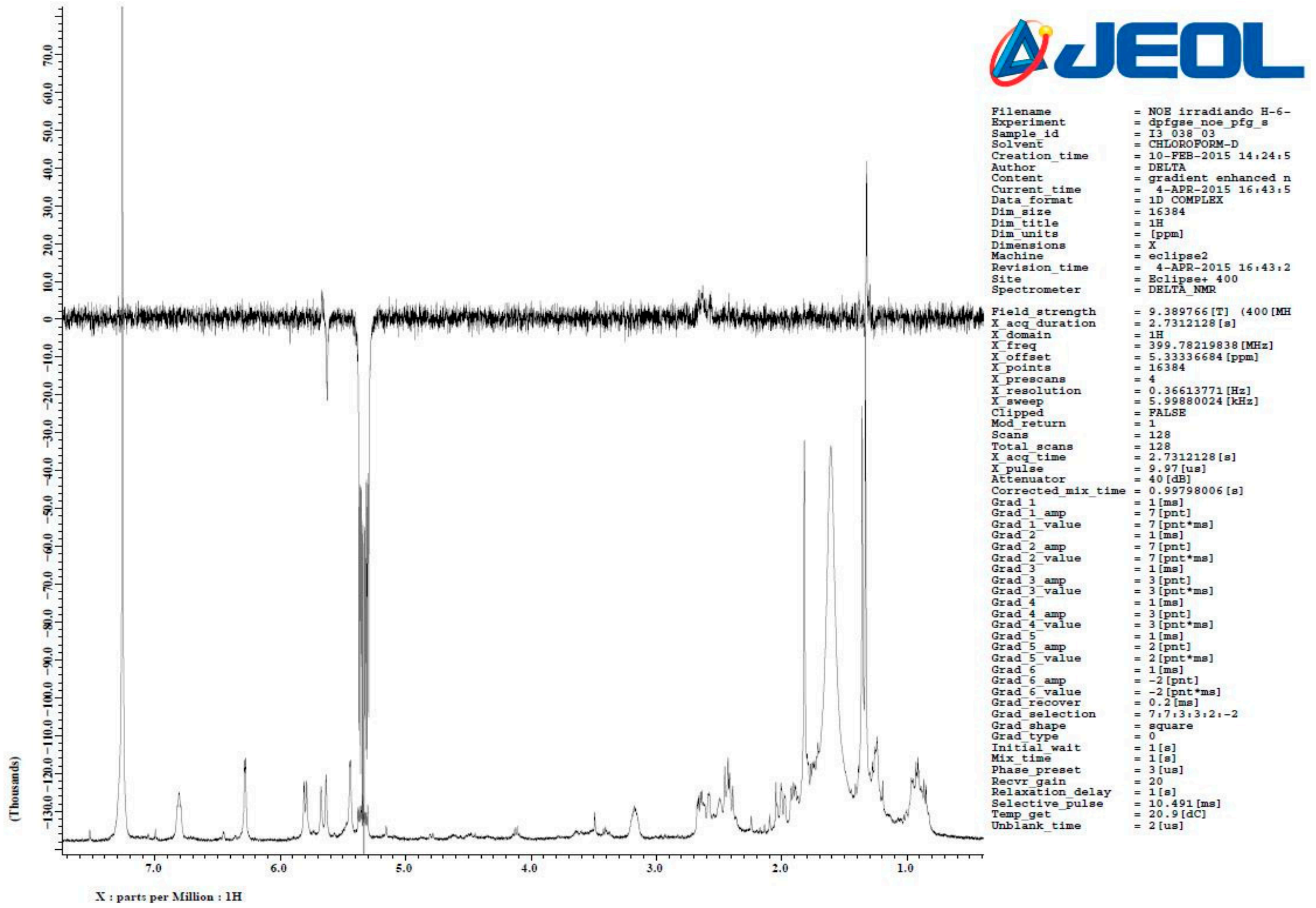

Figure S8. Uprolide N, 1D NOE irradiation on H-6.

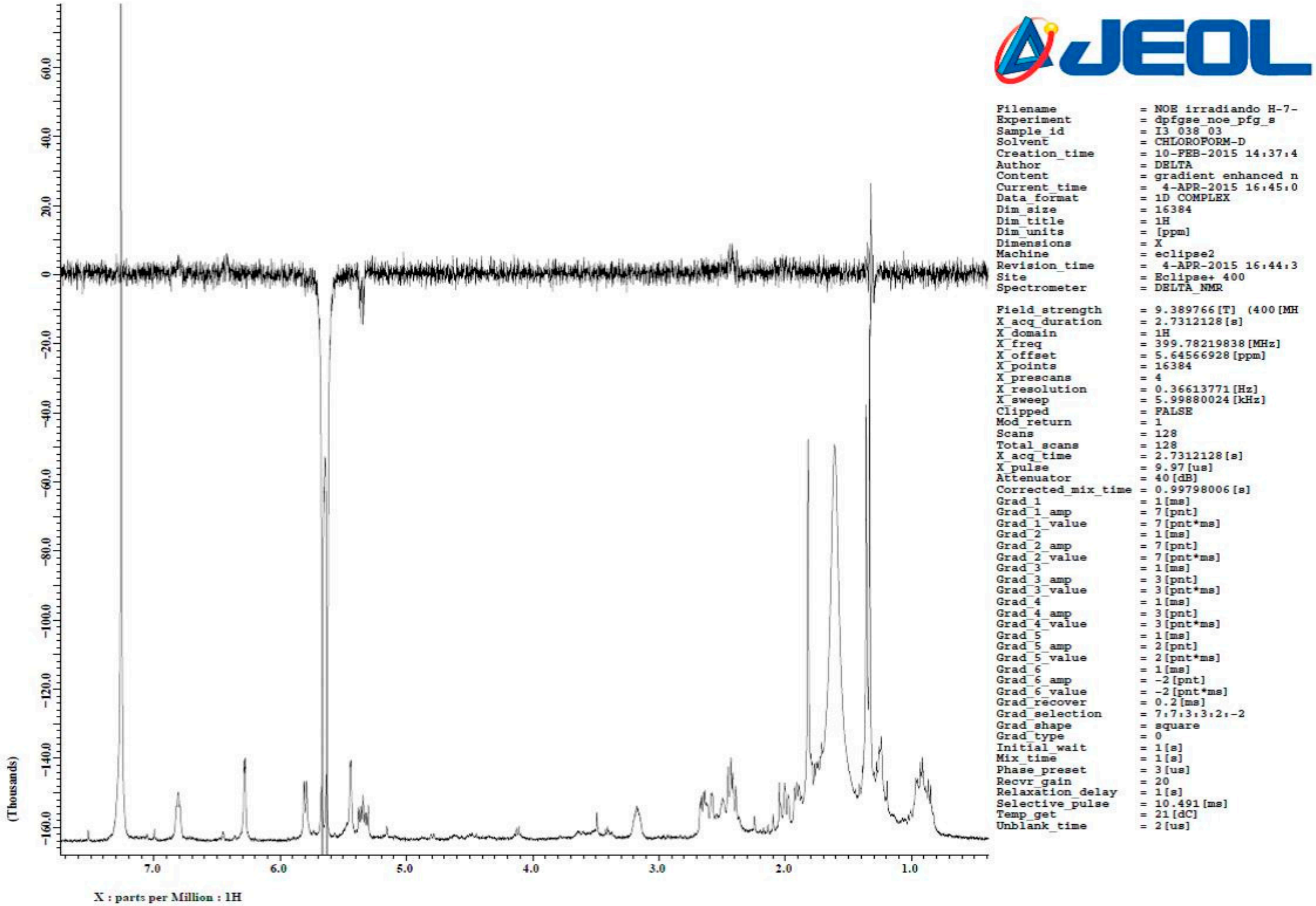

Figure S9. Uprolide N, 1D NOE irradiation on H-7.

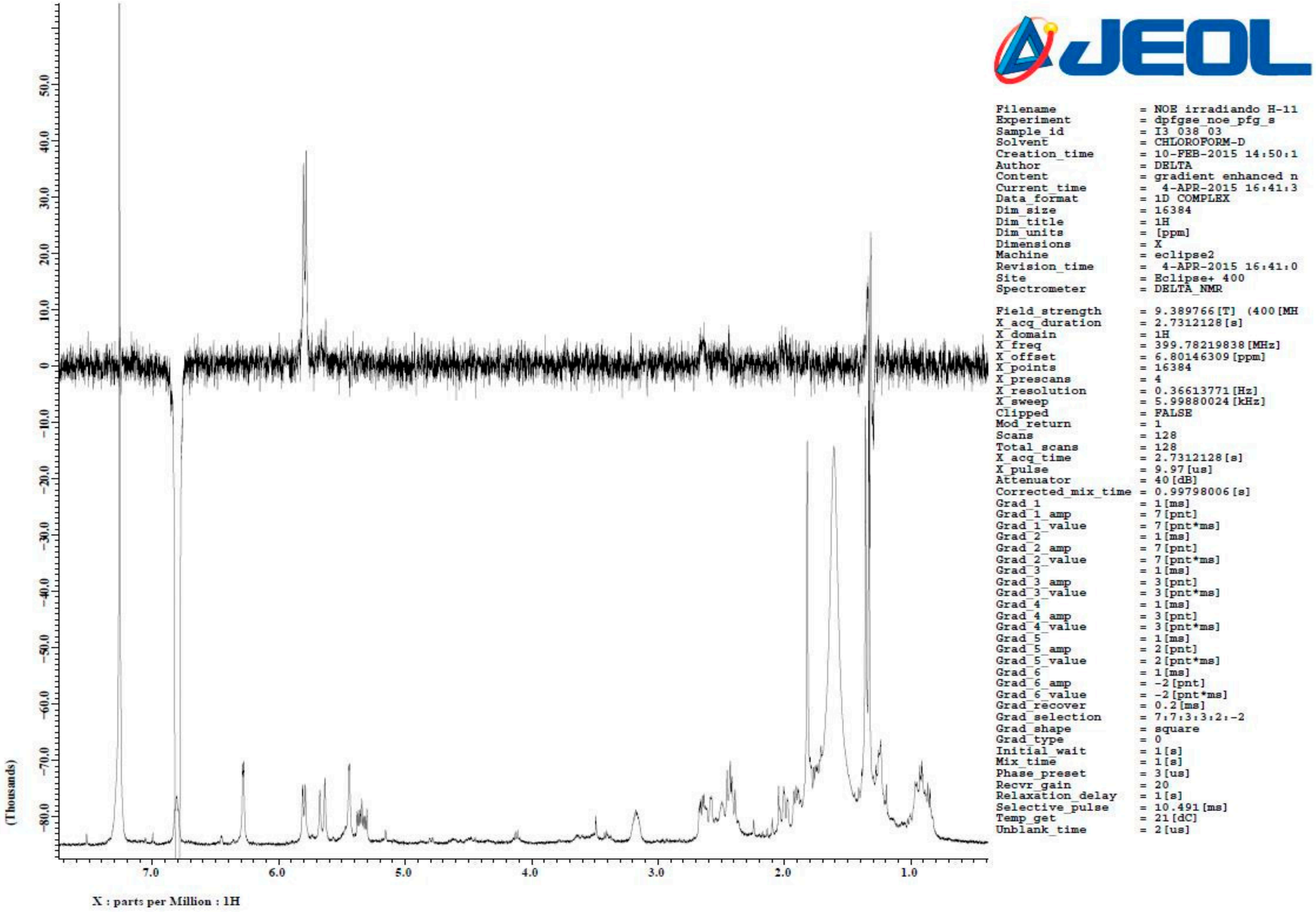

Figure S10. Uprolide N, 1D NOE irradiation on H-11.

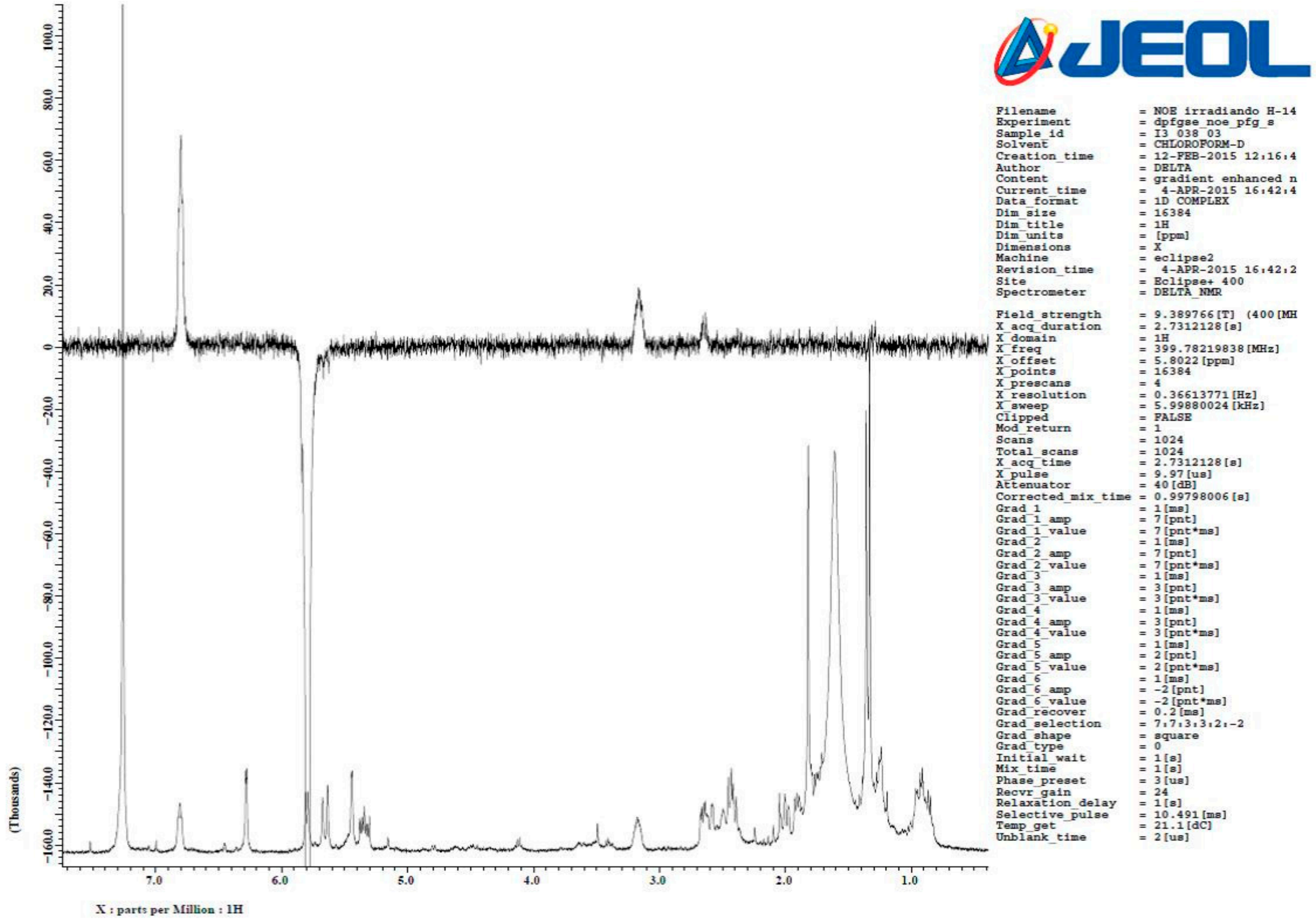

Figure S11. Uprolide N, 1D NOE irradiation on H-14.

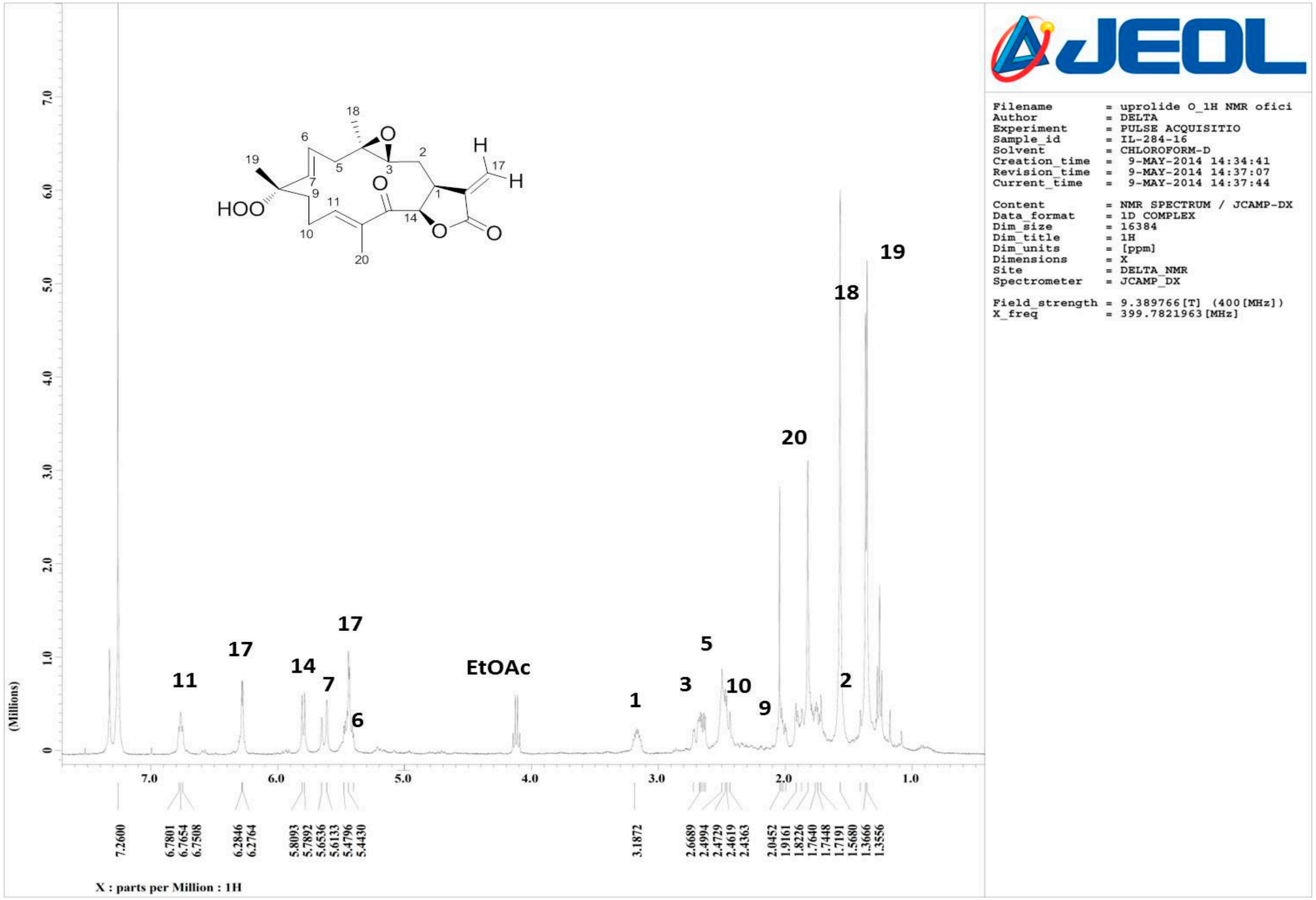

Figure S12. Uprolide O, ^{1}H-NMR spectrum.

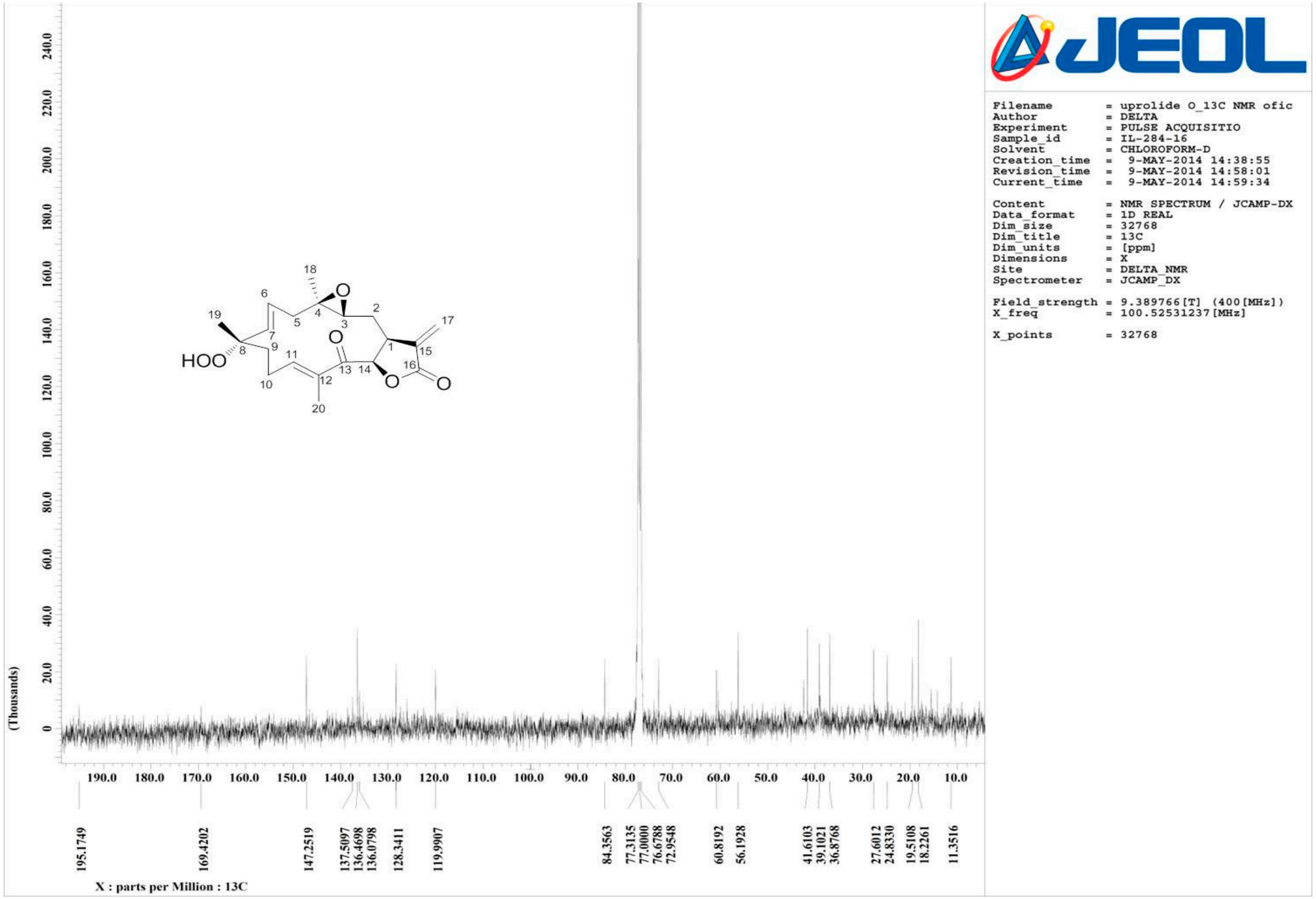

Figure S13. Uprolide O, ^{13}C-NMR spectrum.

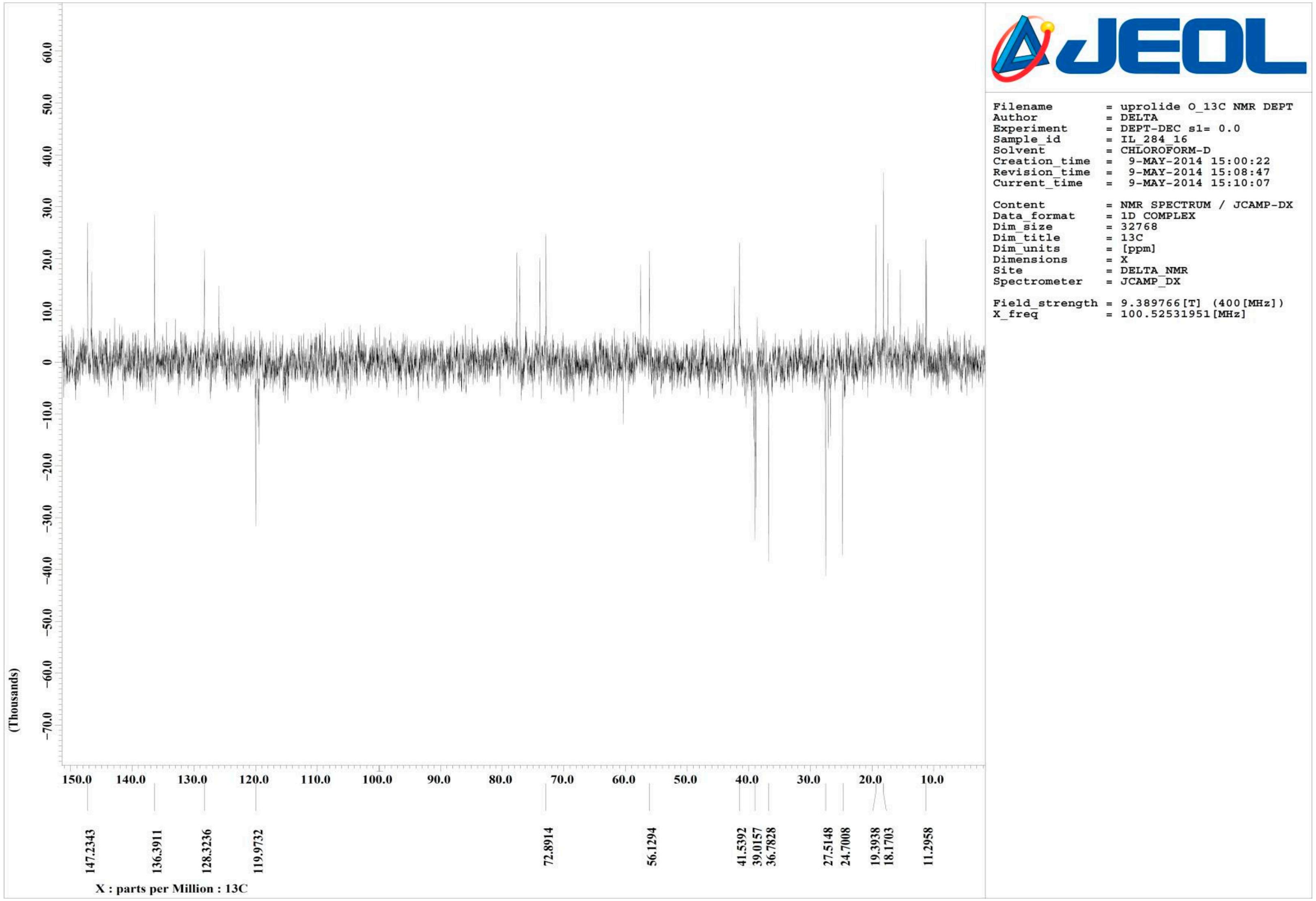

Figure S14. Uprolide O, ^{13}C-NMR-DEPT 135 spectrum.

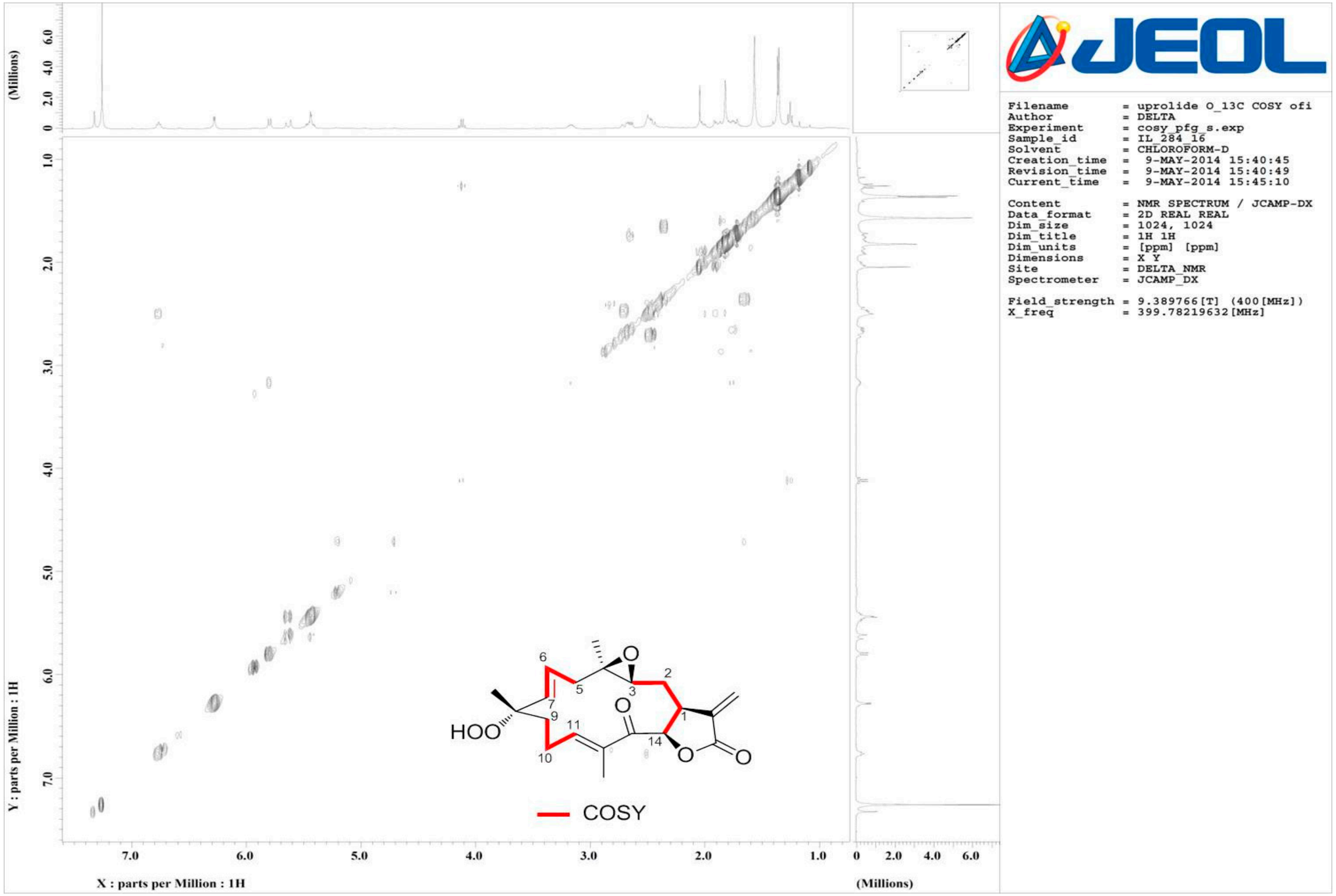

Figure S15. Uprolide O, ^{1}H-^{1}H-COSY spectrum.

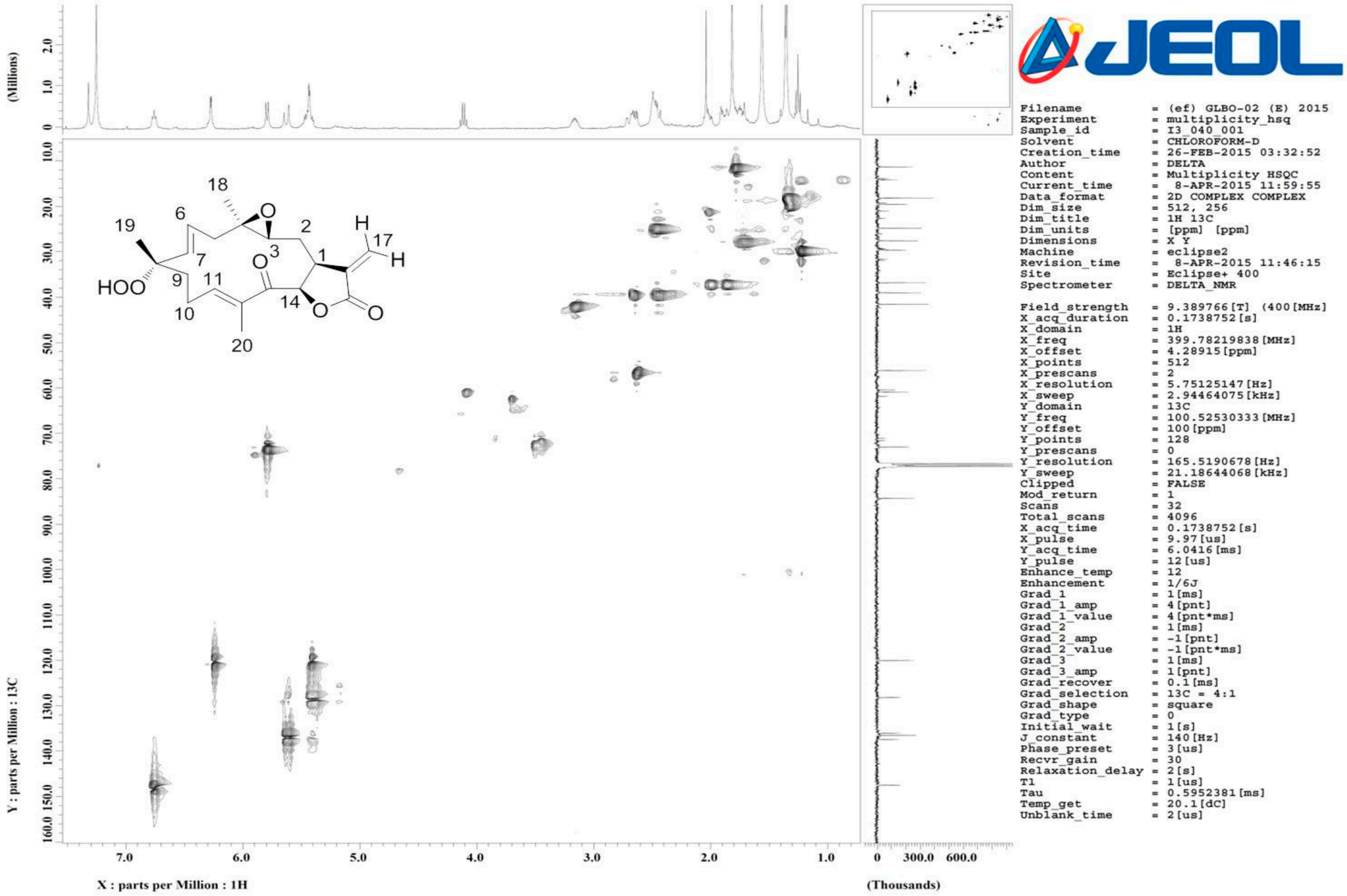

Figure S16. Uprolide O, HSQC spectrum.

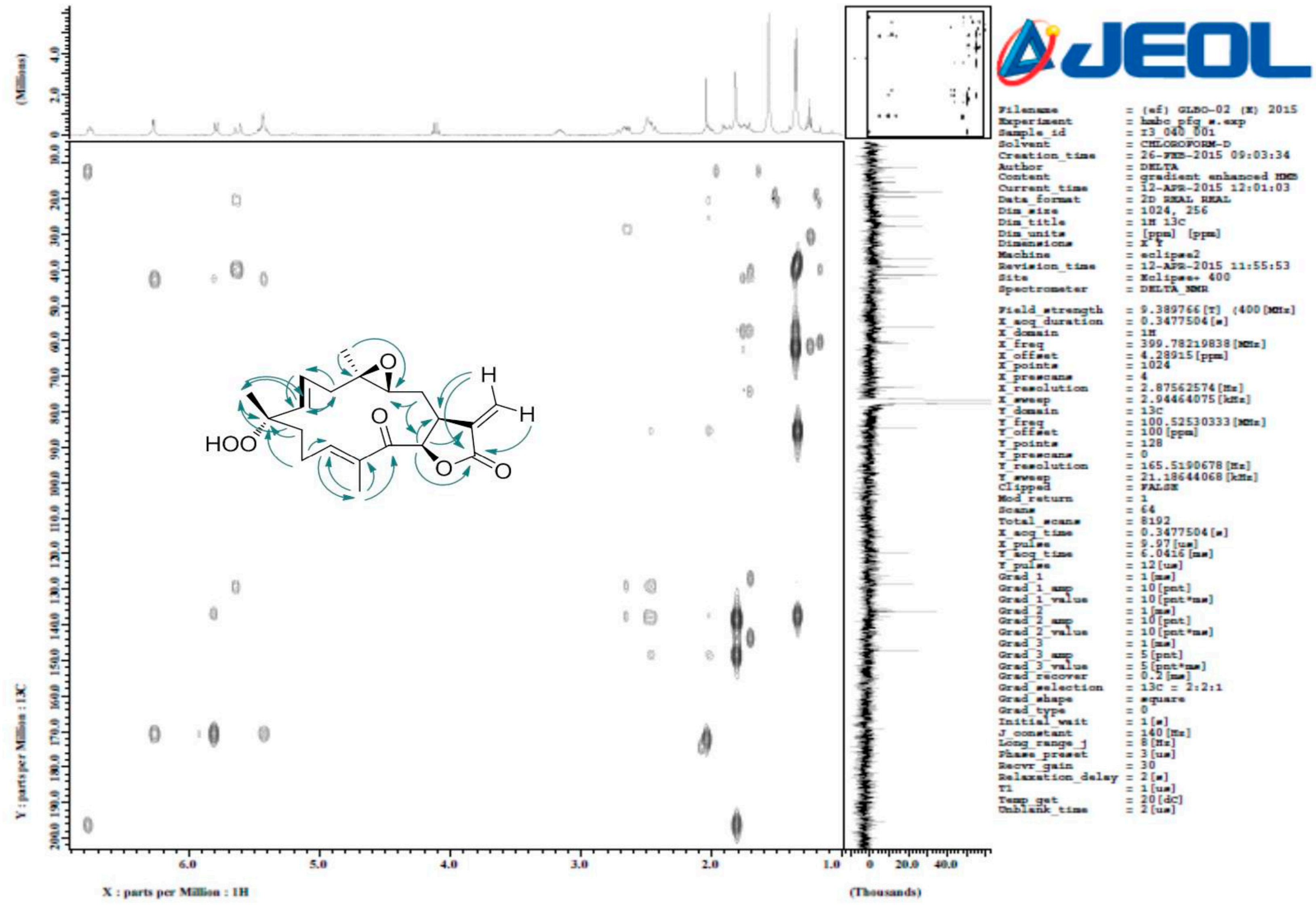

Figure S17. Uprolide O, HMBC spectrum.

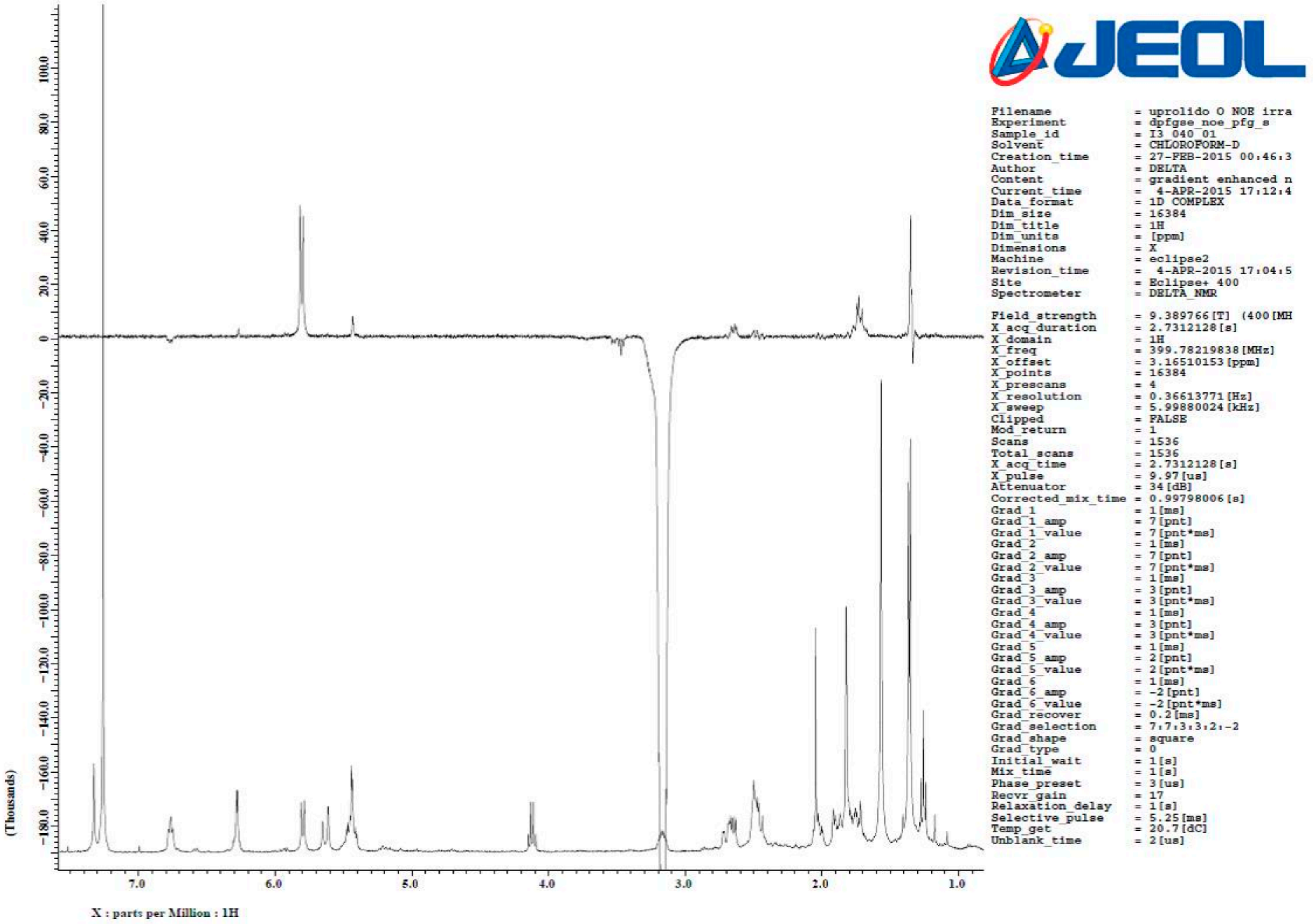

Figure S18. Uprolide O, 1D NOE irradiation on H-1.

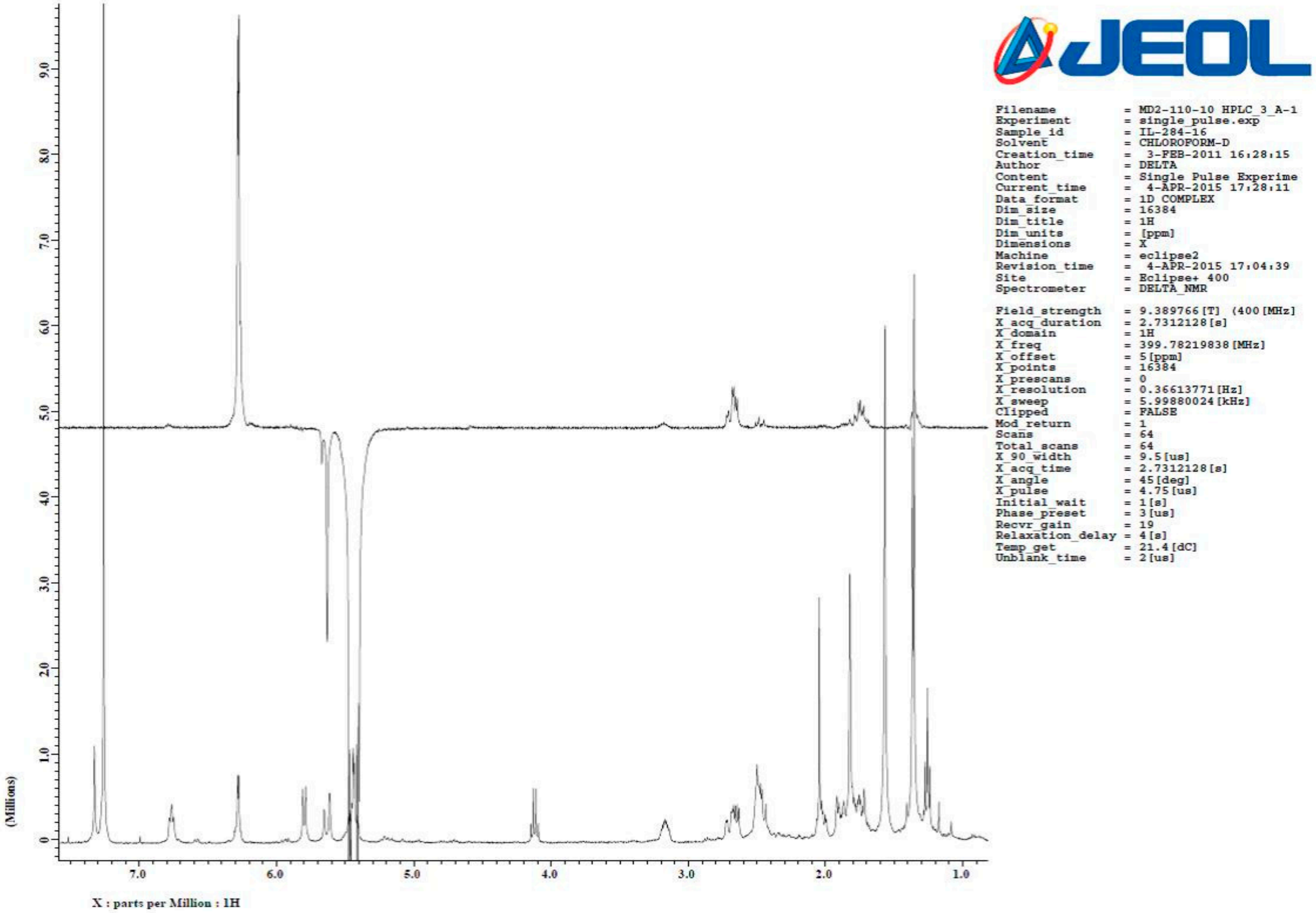

Figure S19. Uprolide O, 1D NOE irradiation on H-6/H-17.

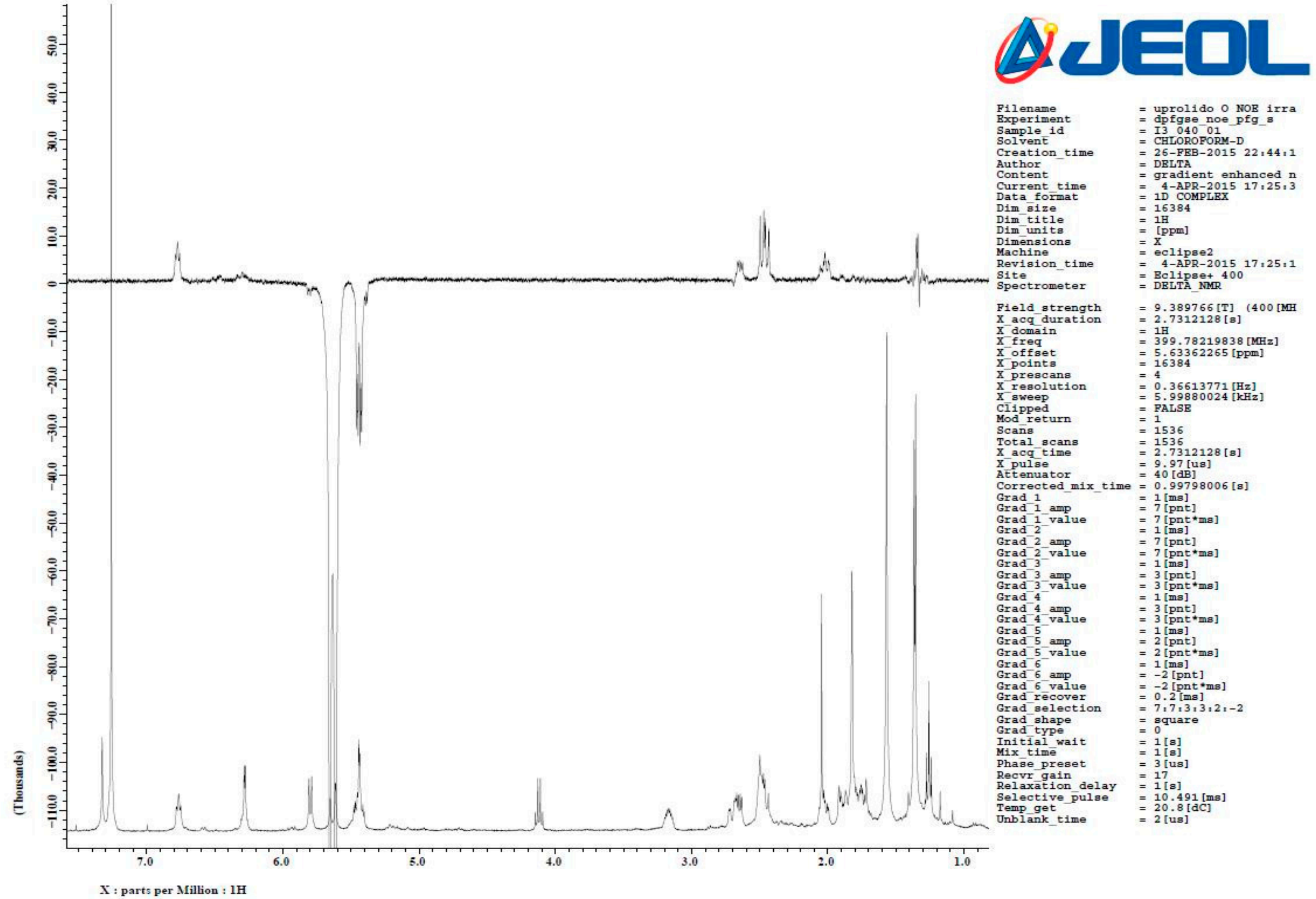

Figure S20. Uprolide O, 1D NOE irradiation on H-7.

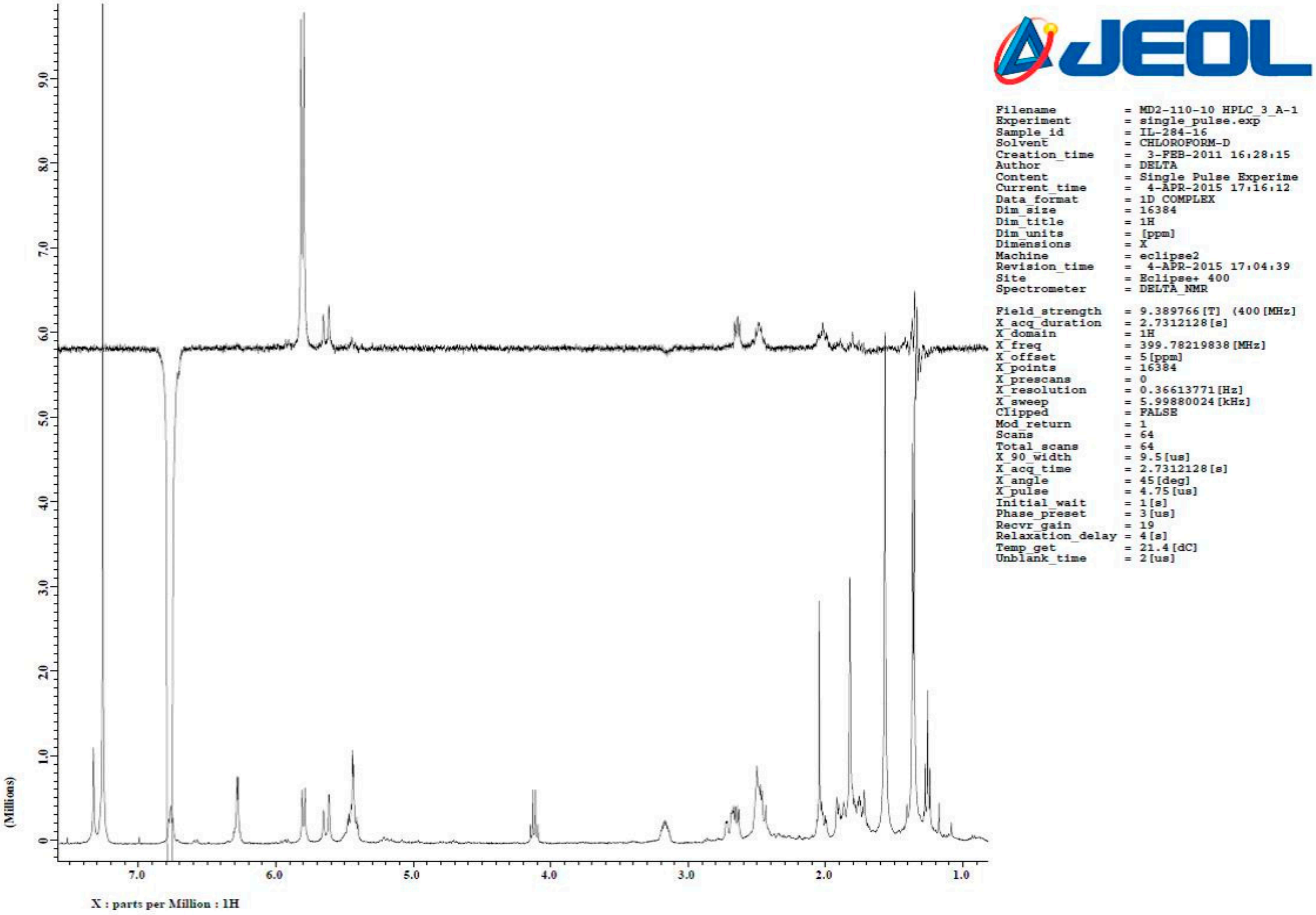

Figure S21. Uprolide O, 1d NOE irradiation on H-11.

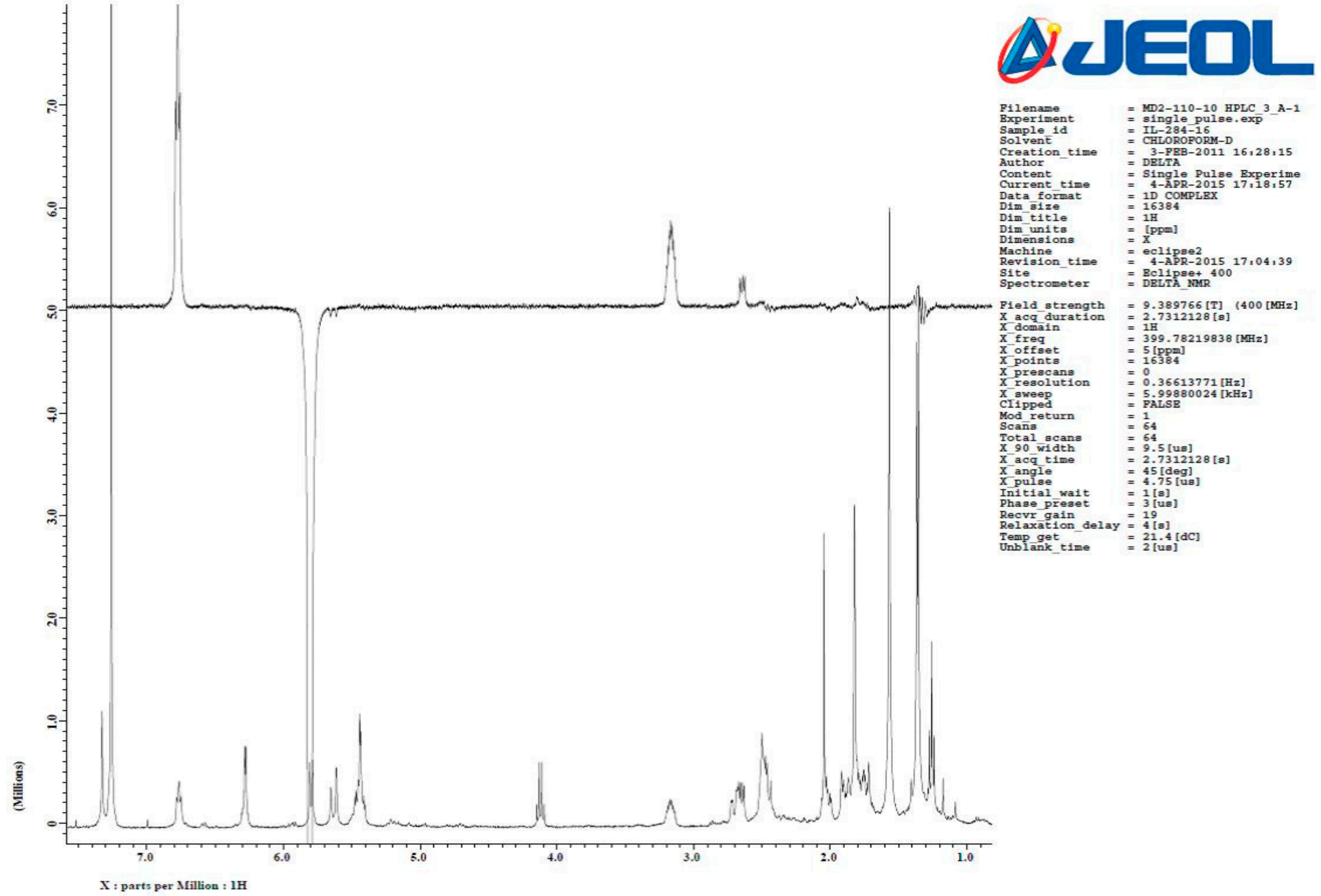

Figure S22. Uprolide O, 1D NOE irradiation on H-14.

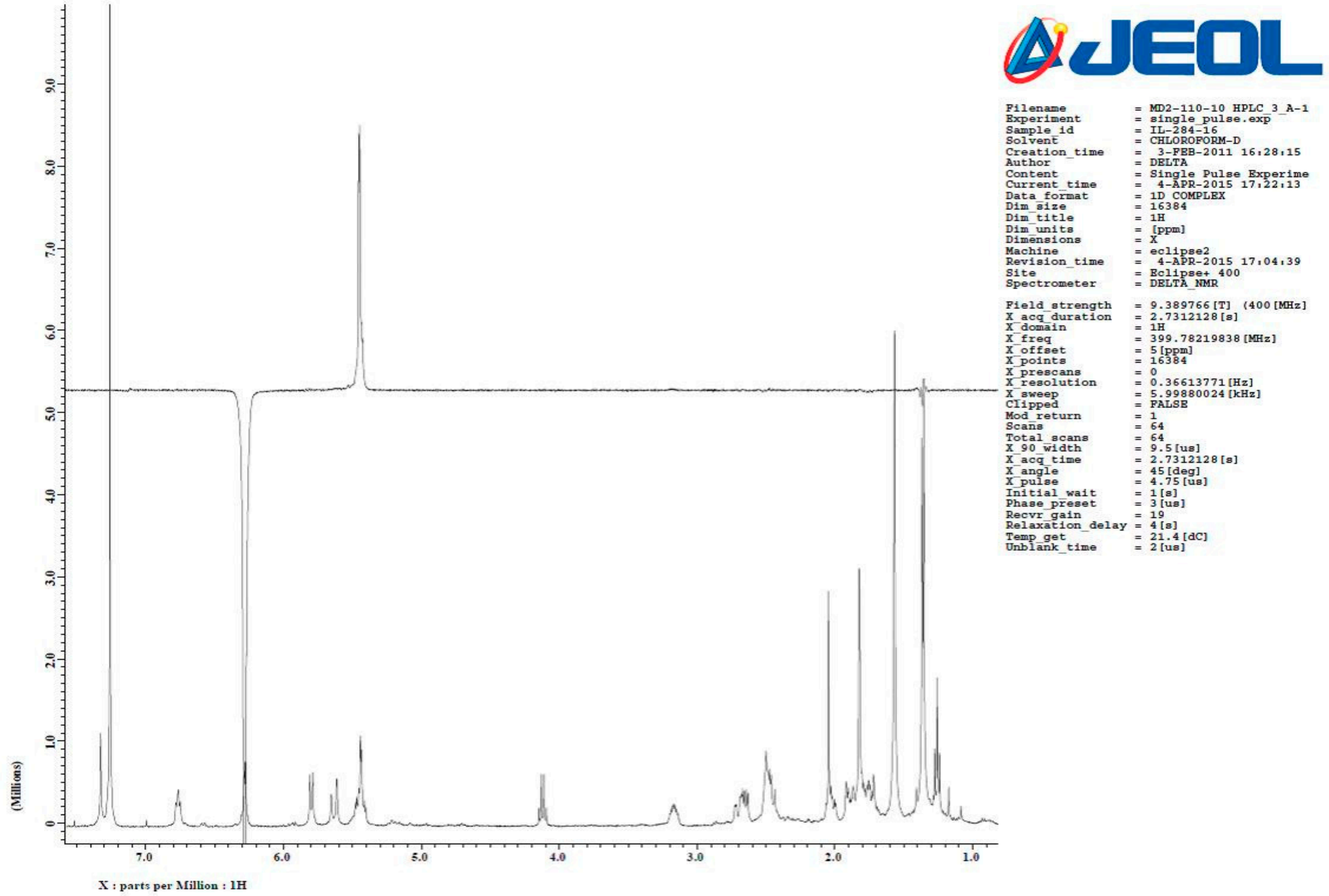

Figure S23. Uprolide O, 1D NOE irradiation on H-17

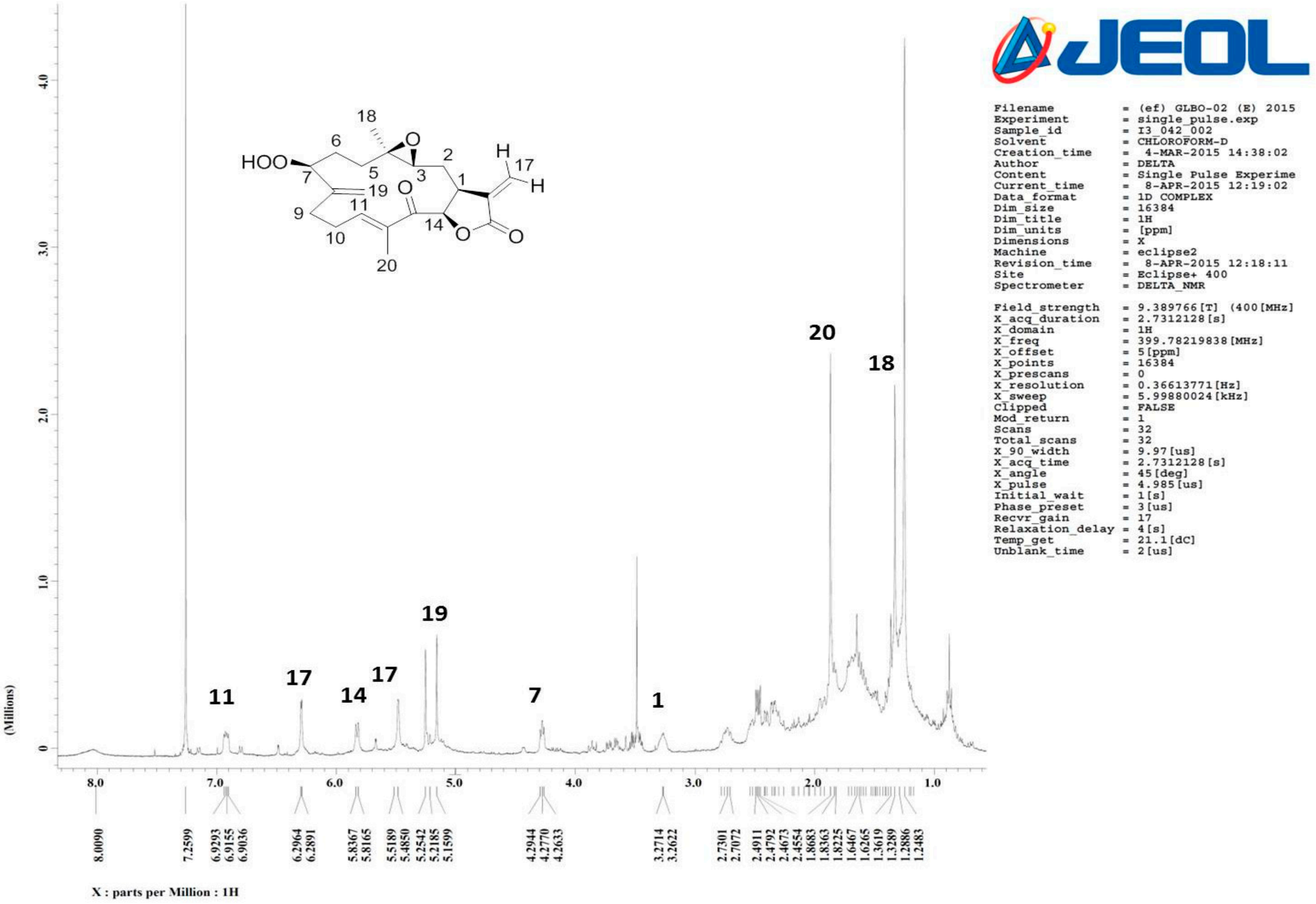

Figure S24. Uprolide P, ¹H-NMR spectrum.

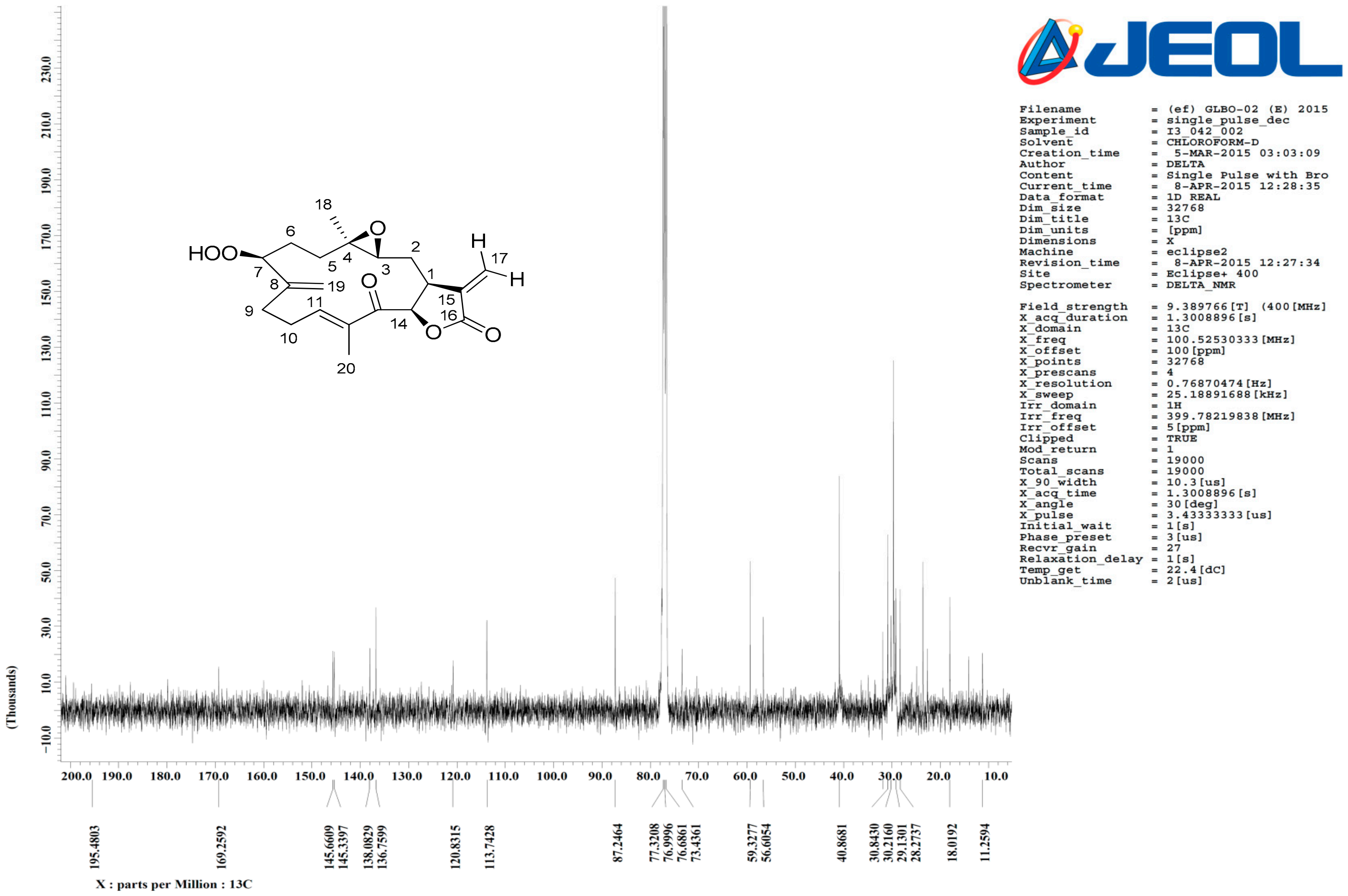

Figure S25. Uprolide P, ^{13}C-NMR spectrum.

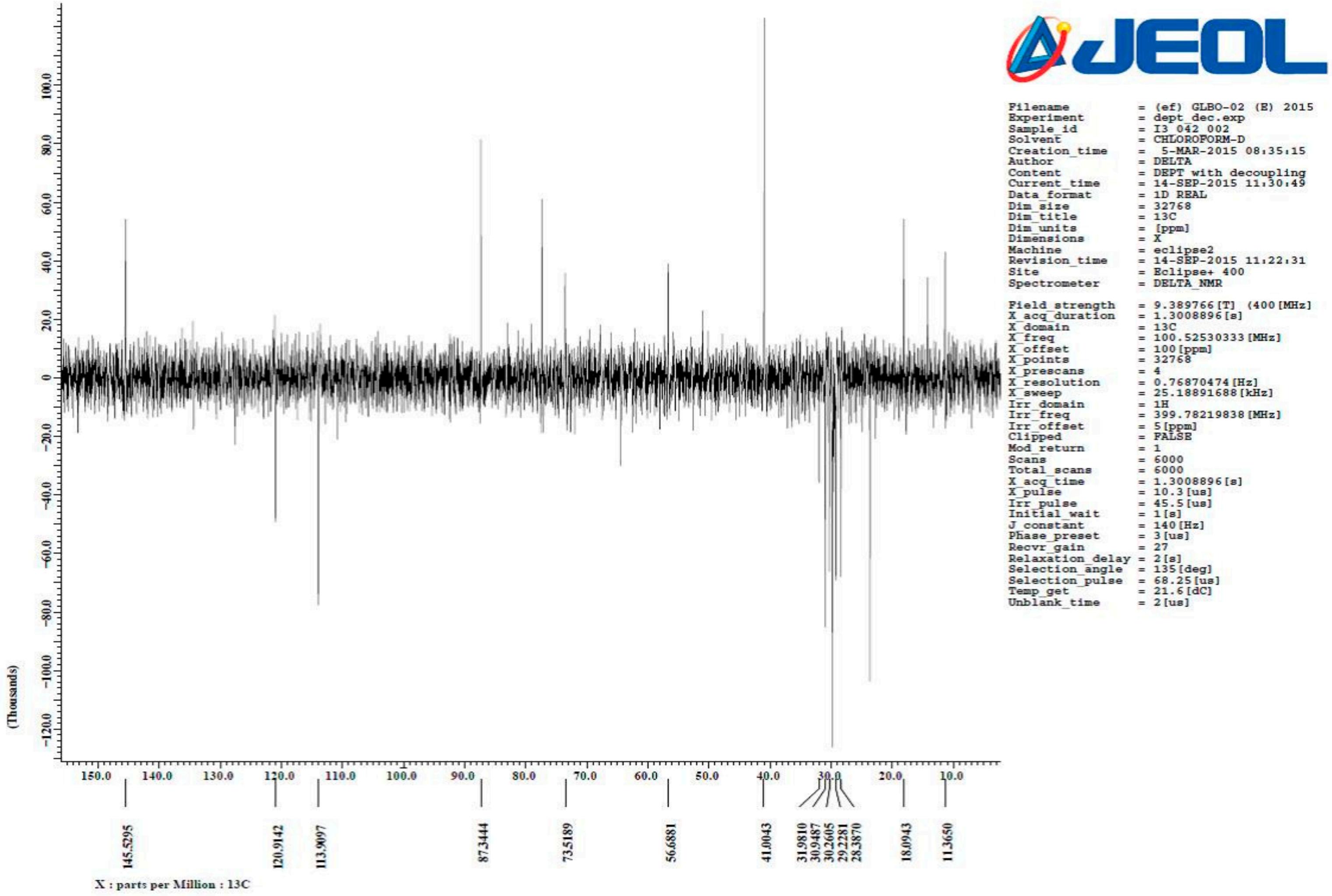

Figure S26. Uprolide P, ^{13}C-NMR-DEPT 135 spectrum.

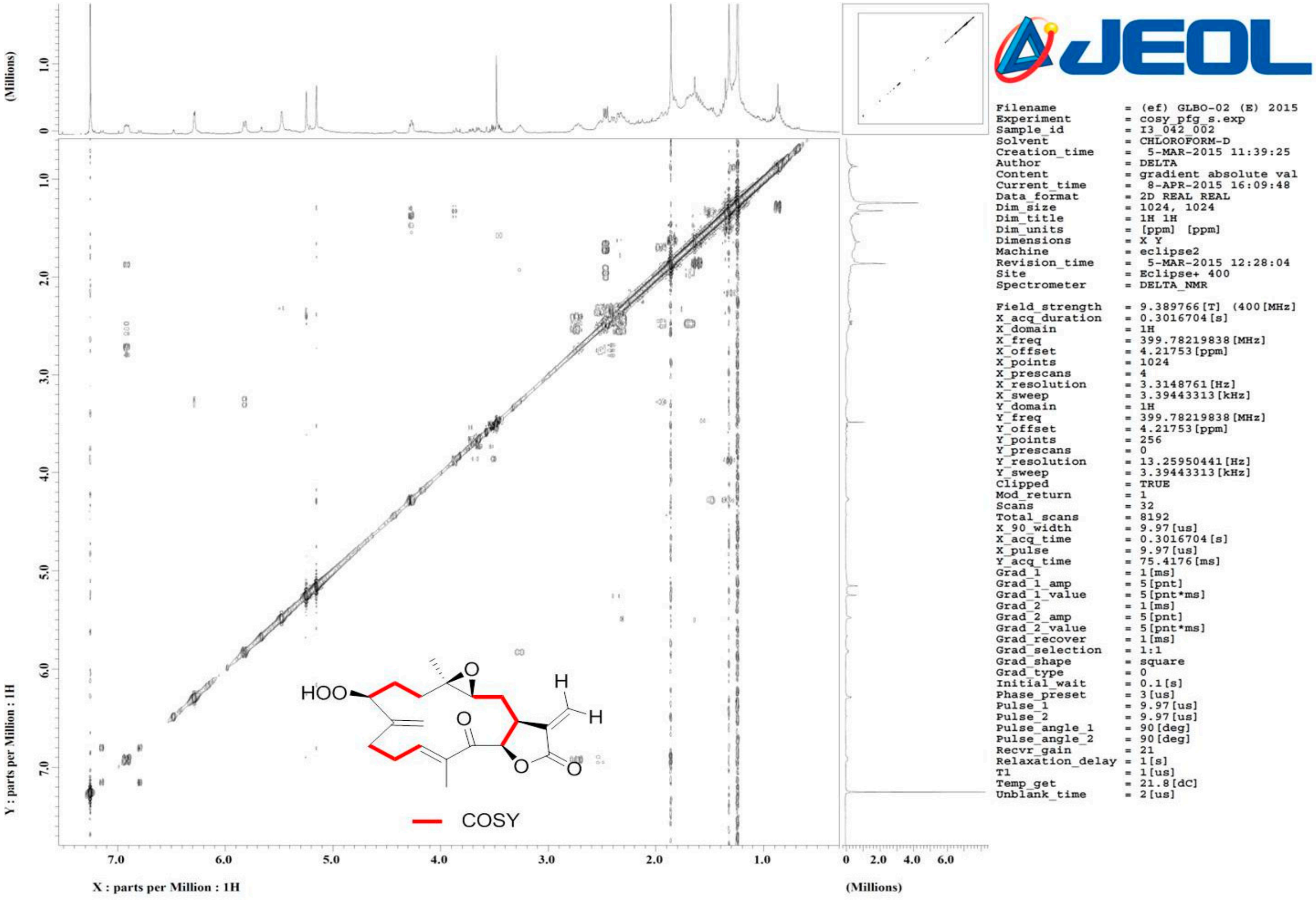

Figure S27. Uprolide P, ^{1}H-^{1}H-COSY spectrum.

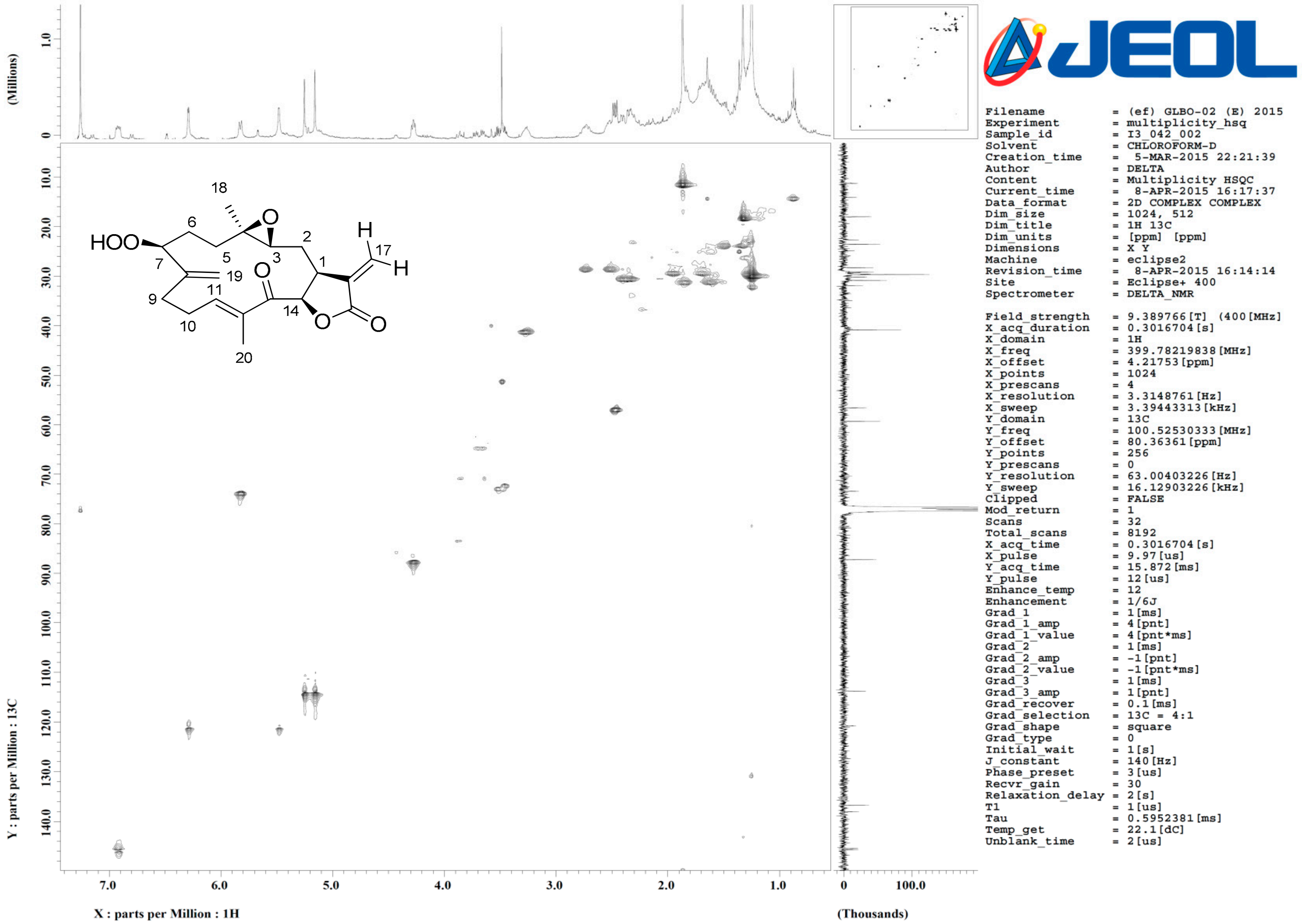

Figure S28. Uprolide P, HSQC spectrum.

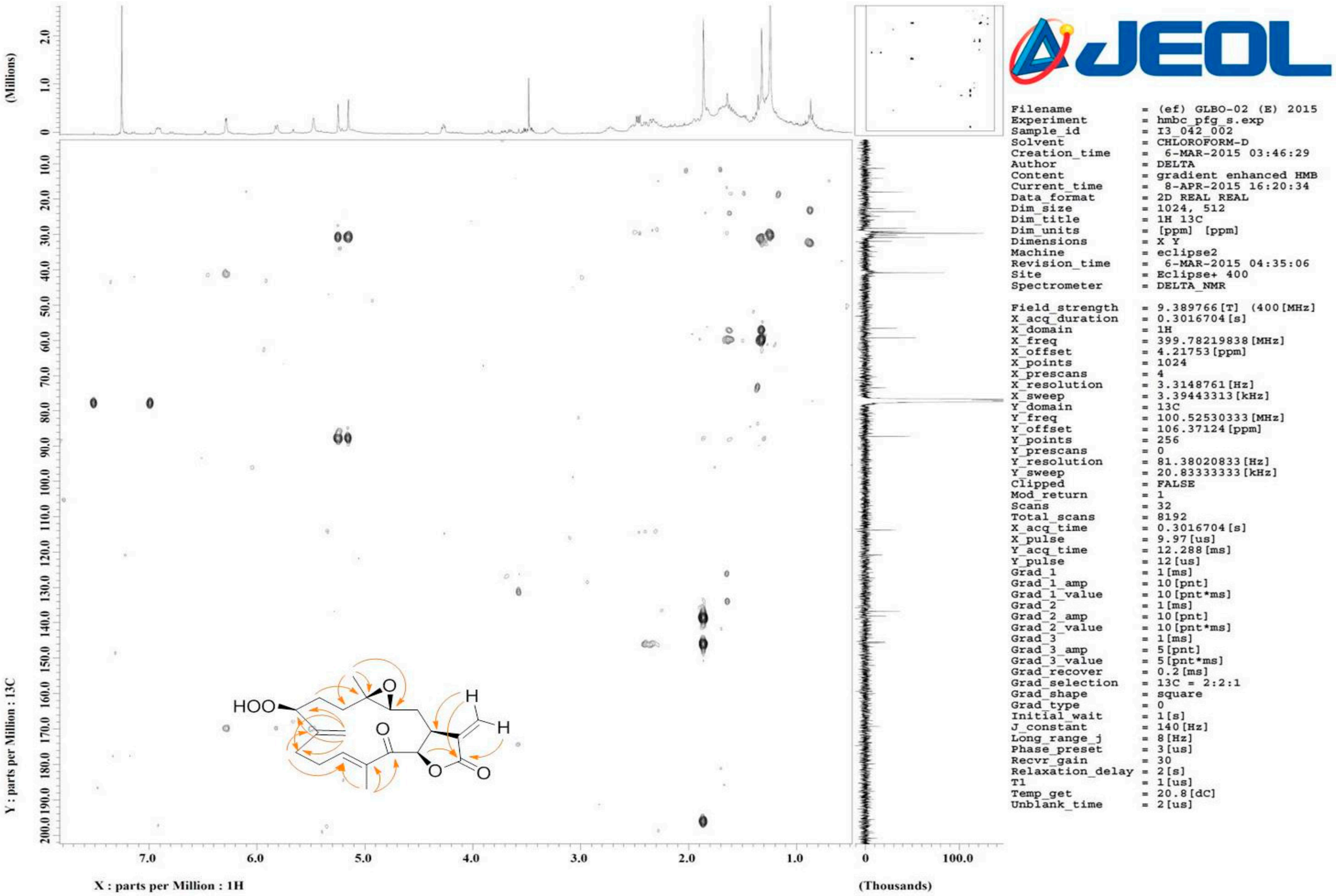

Figure S29. Uprolide P, HMBC spectrum.

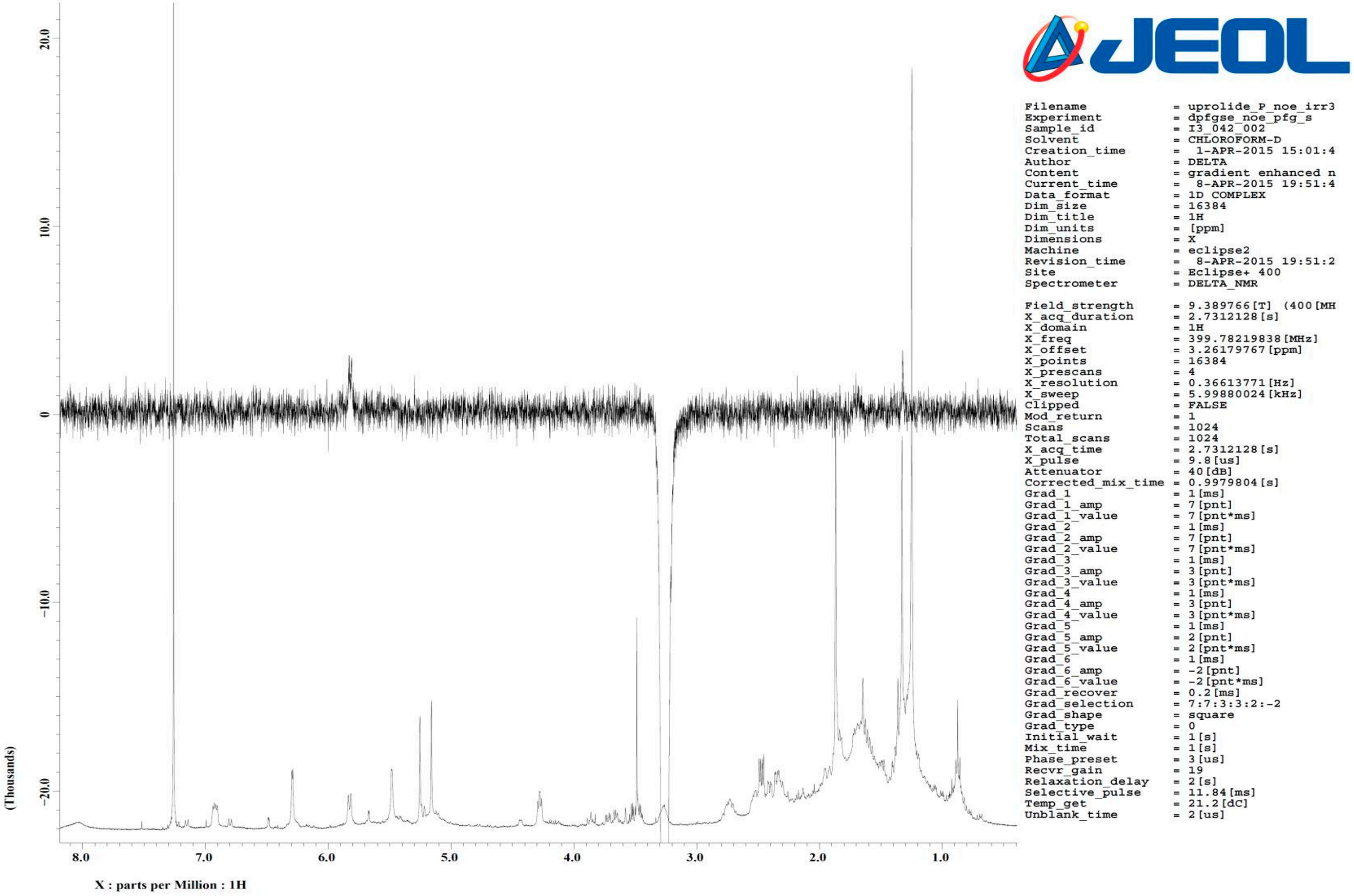

Figure S30. Uprolide P, 1D NOE irradiation on H-1.

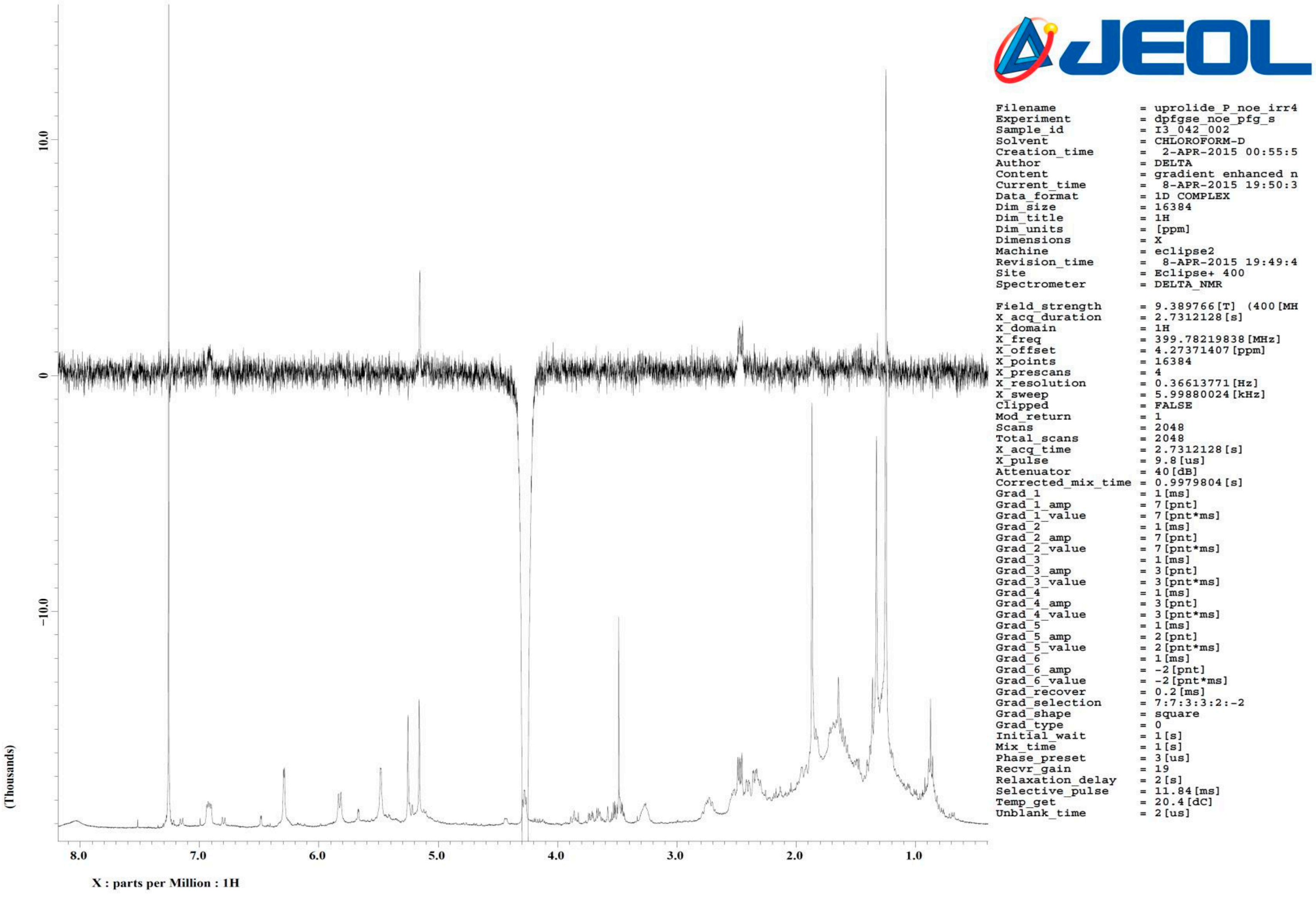

Figure S31. Uprolide P, 1D NOE irradiation on H-7.

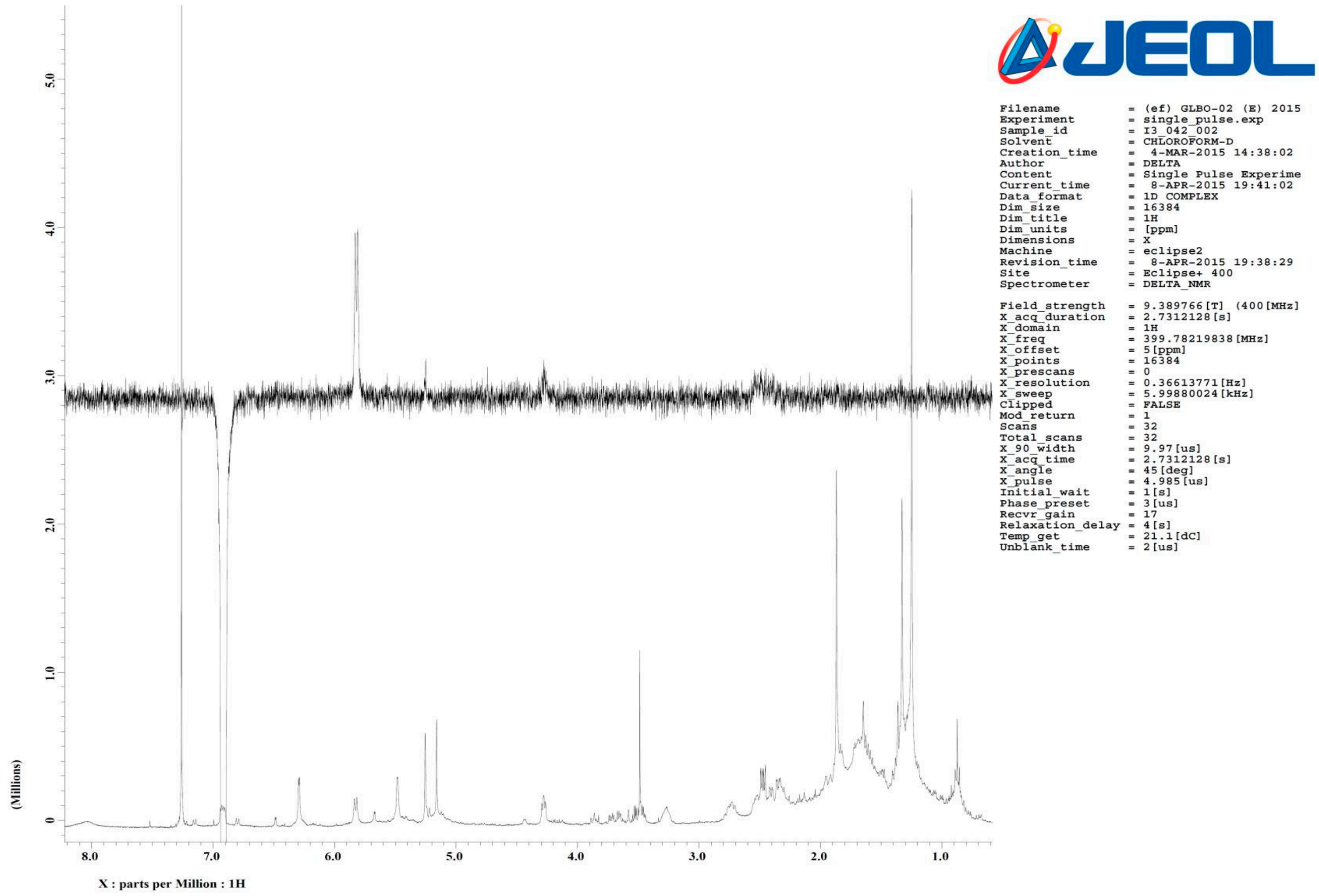

Figure S32. Uprolide P, 1D NOE irradiation on H-11.

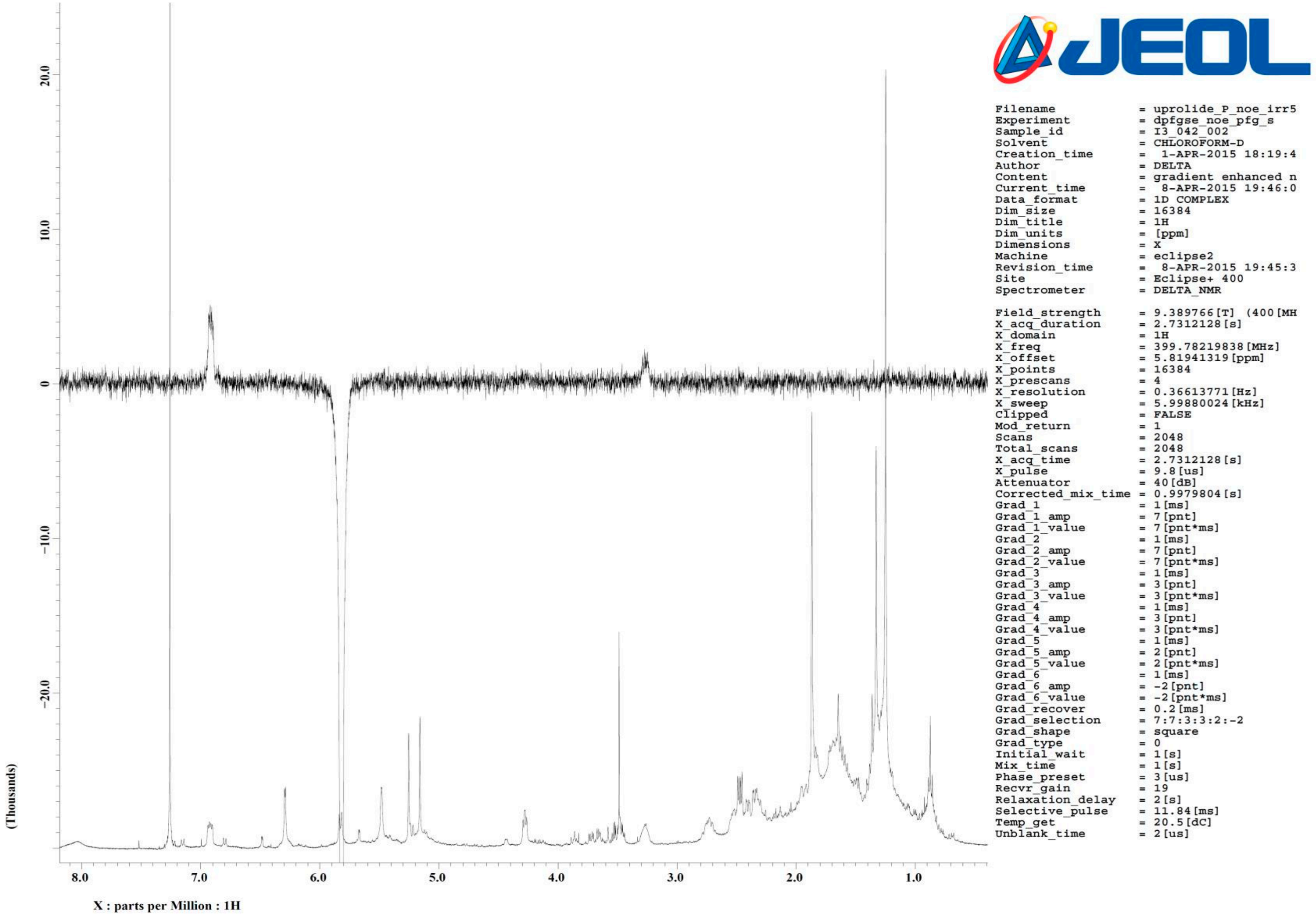

Figure S33. Uprolide P, 1D NOE irradiation on H-14.

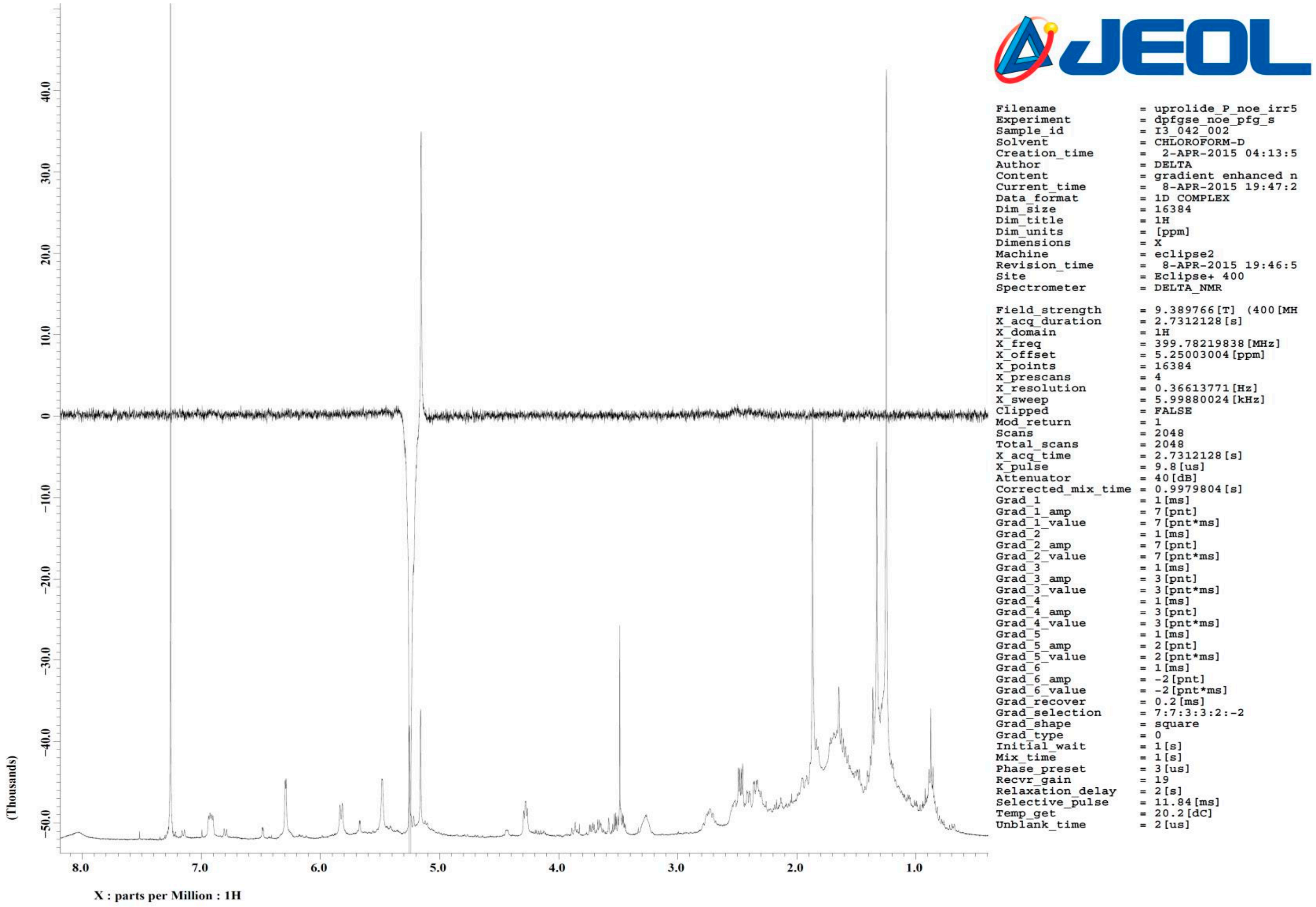

Figure S34. Uprolide P, 1D NOE irradiation on H-19a.

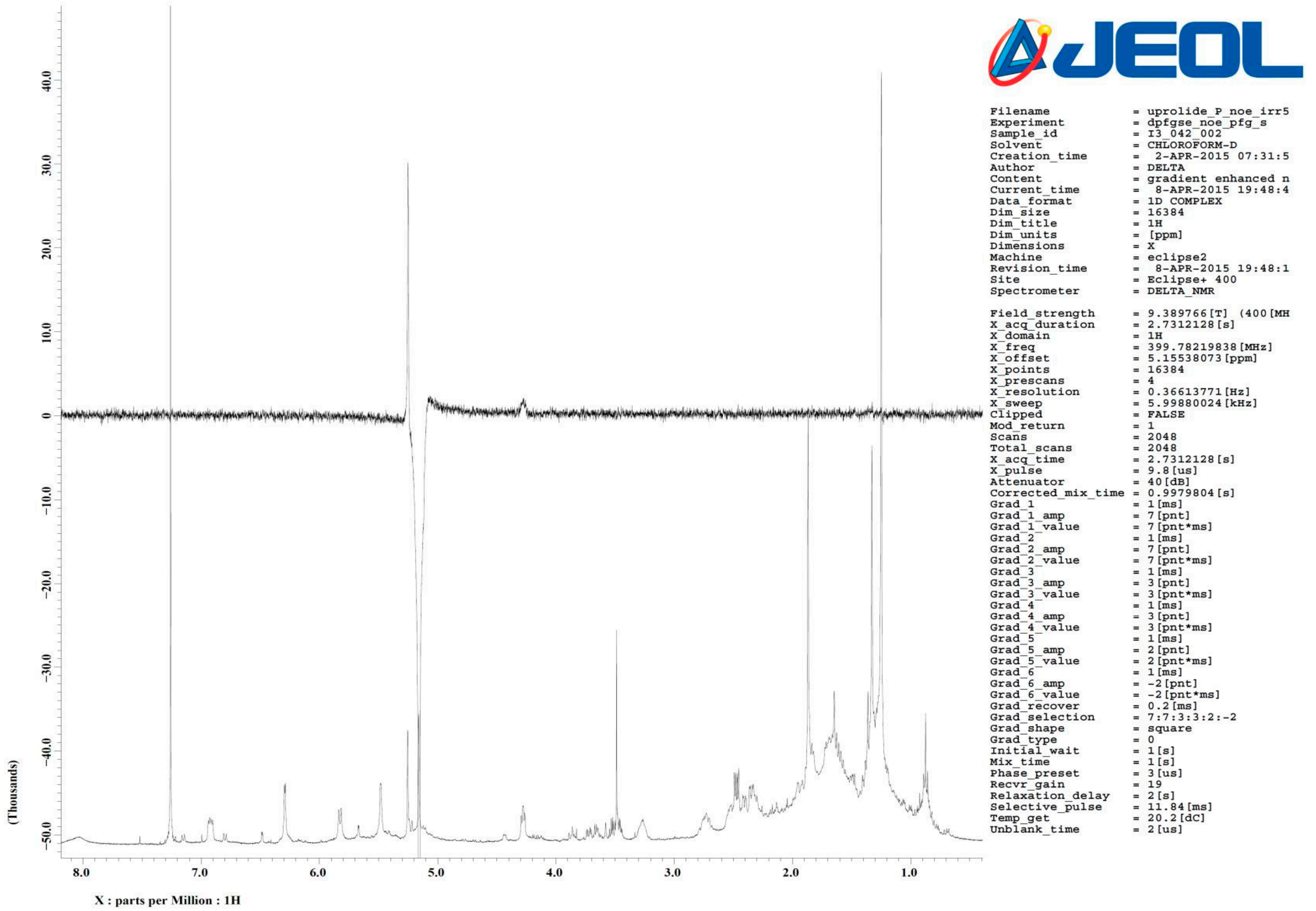

Figure S35. Uprolide P, 1D NOE irradiation on H-19b.

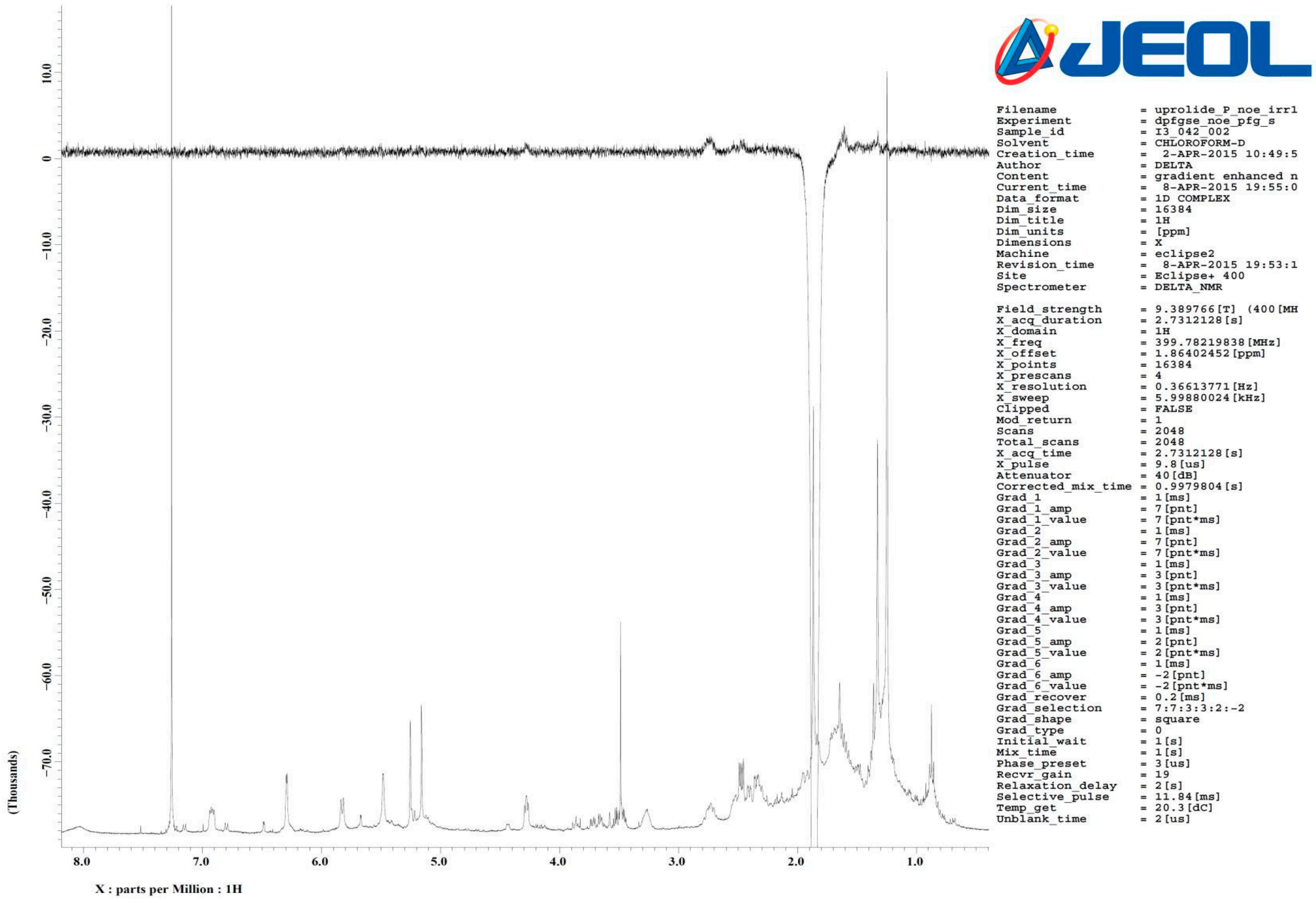

Figure S36. Uprolide P, 1D NOE irradiation on H-20.

Analysis Info

Analysis Name	\\Qt20251\d\Data\Experimentos\DTorres\20160607\Eunicea-E_39-61-ACN_Agil_C18_P1-A-2_01_3487.d	Acquisition Date	6/8/2016 12:57:42 PM
Method	tune_wide_lc.m	Operator	BDAL@DE
Sample Name	Eunicea-E_39-61-ACN_Agil_C18	Instrument	micrOTOF-Q III 228888.20451
Comment			

Acquisition Parameter

Source Type	ESI	Ion Polarity	Positive	Set Nebulizer	2.0 Bar
Focus	Active	Set Capillary	4500 V	Set Dry Heater	200 °C
Scan Begin	50 m/z	Set End Plate Offset	-500 V	Set Dry Gas	10.0 l/min
Scan End	3000 m/z	Set Collision Cell RF	680.0 Vpp	Set Divert Valve	Waste

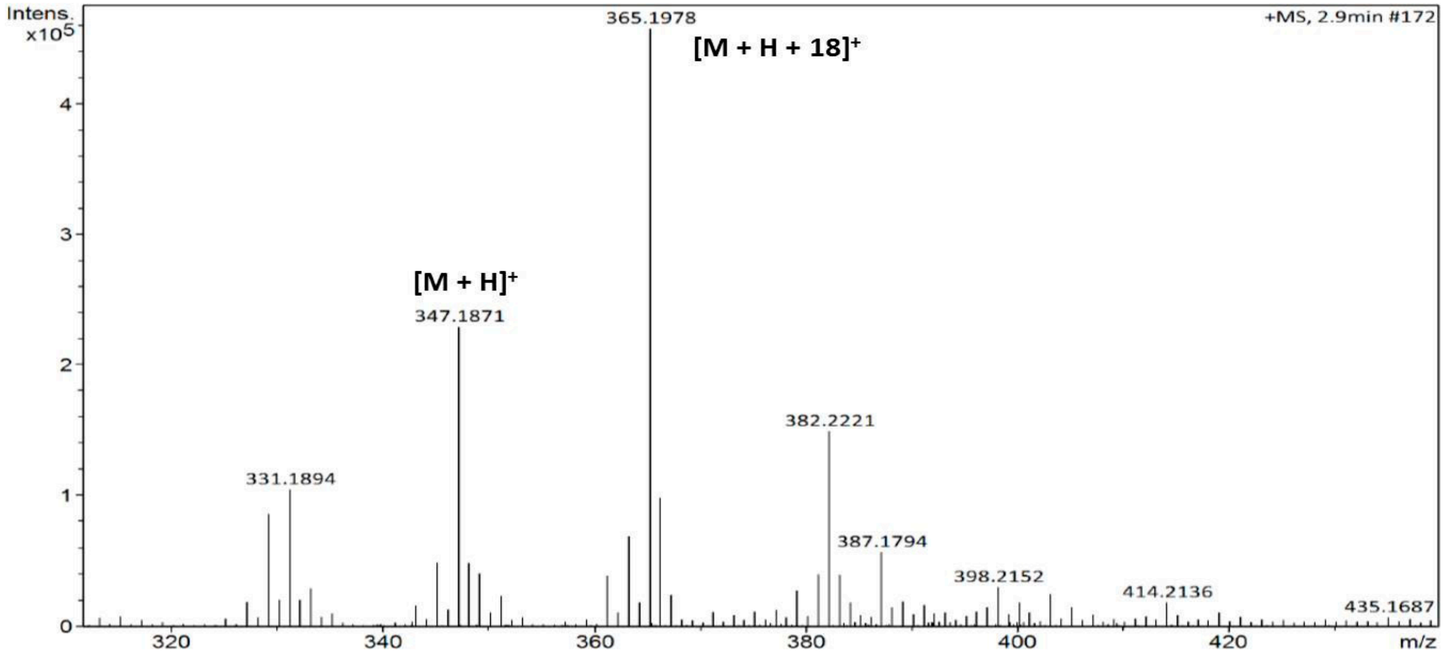

Meas. m/z	#	Ion Formula	m/z	err [ppm]	mSigma	# Sigma	Score	rdb	e⁻ Conf	N-Rule
347.1871	1	C20H27O5	347.1853	-5.0	74.9	1	100.00	7.5	even	ok

Figure S37. Uprolide N, HRESITOF-MS.

Compound Spectrum SmartFormula Report

Analysis Info

Analysis Name	G:\micrOTOF-QIII Data (ESI & APCI)\Users\DTorres\uprolido-O_20150421_res.d
Method	tune_wide-low_dir_ms2_gard2014.m
Sample Name	uprolido-O_20150421_res
Comment	Concentration: 50 ug/mL<br>Prepared on: 20150421<br><br>Spiked with Reserpine standard 0.5 ug/mL for lock mass calibration<br>Dissolved in 100% Methanol
Acquisition Date	4/21/2015 12:07:50 PM
Operator	BDAL@DE
Instrument	micrOTOF-Q III 228888.10451

Acquisition Parameter

Source Type	ESI	Ion Polarity	Positive	Set Nebulizer	0.3 Bar
Focus	Active	Set Capillary	4500 V	Set Dry Heater	200 °C
Scan Begin	50 m/z	Set End Plate Offset	-500 V	Set Dry Gas	4.0 l/min
Scan End	3000 m/z	Set Collision Cell RF	265.0 Vpp	Set Divert Valve	Waste

#	RT [s]	Area	Int. Type	I	S/N	Chromatogram	Max. m/z	FWHM [s]
n.a.	5.2	n.a.	Single spectrum	n.a.	n.a.	n.a.	385.1615	n.a.

+MS, 5.2s #12

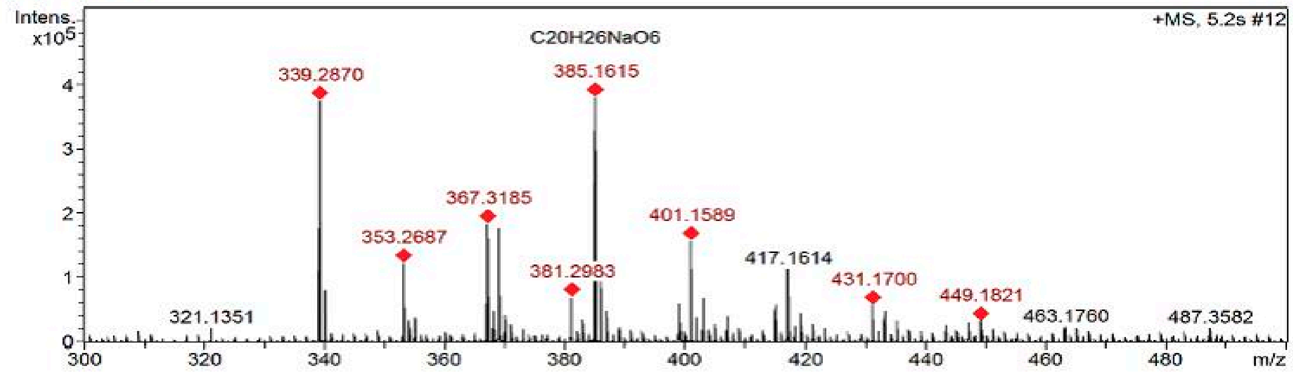

Meas. m/z	#	Ion Formula	m/z	err [ppm]	mSigma	# Sigma	Score	rdb	e⁻ Conf	N-Rule
385.1615	1	C20H26NaO6	385.1622	1.7	55.0	1	100.00	7.5	even	ok

Figure S38. Uprolide O, HRESITOF-MS.

Compound Spectrum SmartFormula Report

Analysis Info

		Acquisition Date	5/31/2016 3:30:36 PM
Analysis Name	\\Qt20251\d\Data\Experimentos\DTorres\20160531\Uprolido-p+reserpine.d		
Method	tune_wide.m	Operator	BDAL@DE
Sample Name	Uprolido-p	Instrument	micrOTOF-Q III 228888.20451
Comment	Concentration: 100 ug/mL<br>Prepared on: 20160531<br><br>Spiked with reserpine 0.5 ug/mL<br>Dissolved in 100% Methanol		

Acquisition Parameter

Source Type	ESI	Ion Polarity	Positive	Set Nebulizer	0.3 Bar
Focus	Active	Set Capillary	4500 V	Set Dry Heater	200 °C
Scan Begin	50 m/z	Set End Plate Offset	-500 V	Set Dry Gas	4.0 l/min
Scan End	3000 m/z	Set Collision Cell RF	550.0 Vpp	Set Divert Valve	Source

#	RT [min]	Area	Int. Type	I	S/N	Chromatogram	Max. m/z	FWHM [min]
n.a.	0.2	n.a.	Single spectrum	n.a.	n.a.	n.a.	369.1665	n.a.

+MS, 0.2min #11

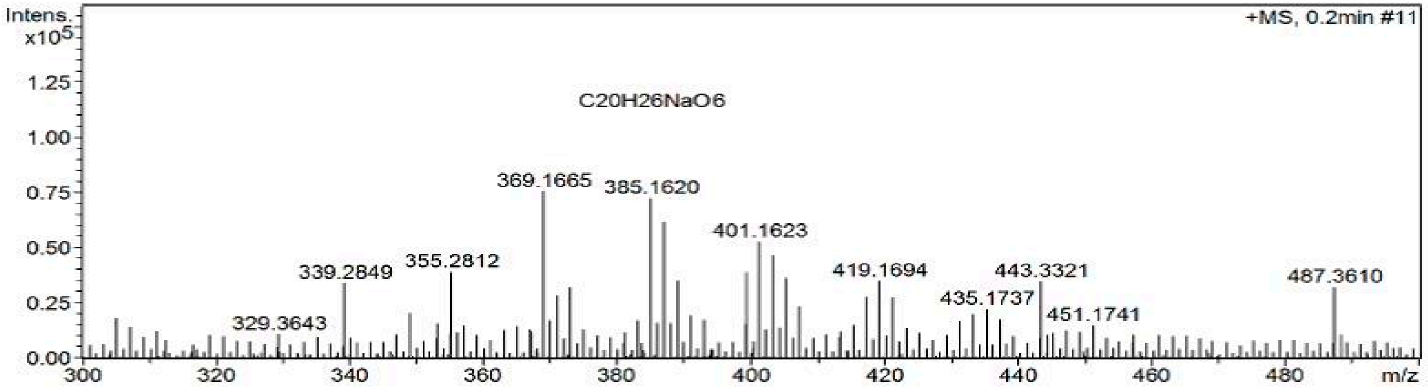

Meas. m/z	#	Ion Formula	m/z	err [ppm]	mSigma	# Sigma	Score	rdb	e¯ Conf	N-Rule
385.1620	1	C20H26NaO6	385.1622	0.4	422.9	1	100.00	7.5	even	ok

Figure S39. Uprolide P, HRESITOF-MS spectrum.

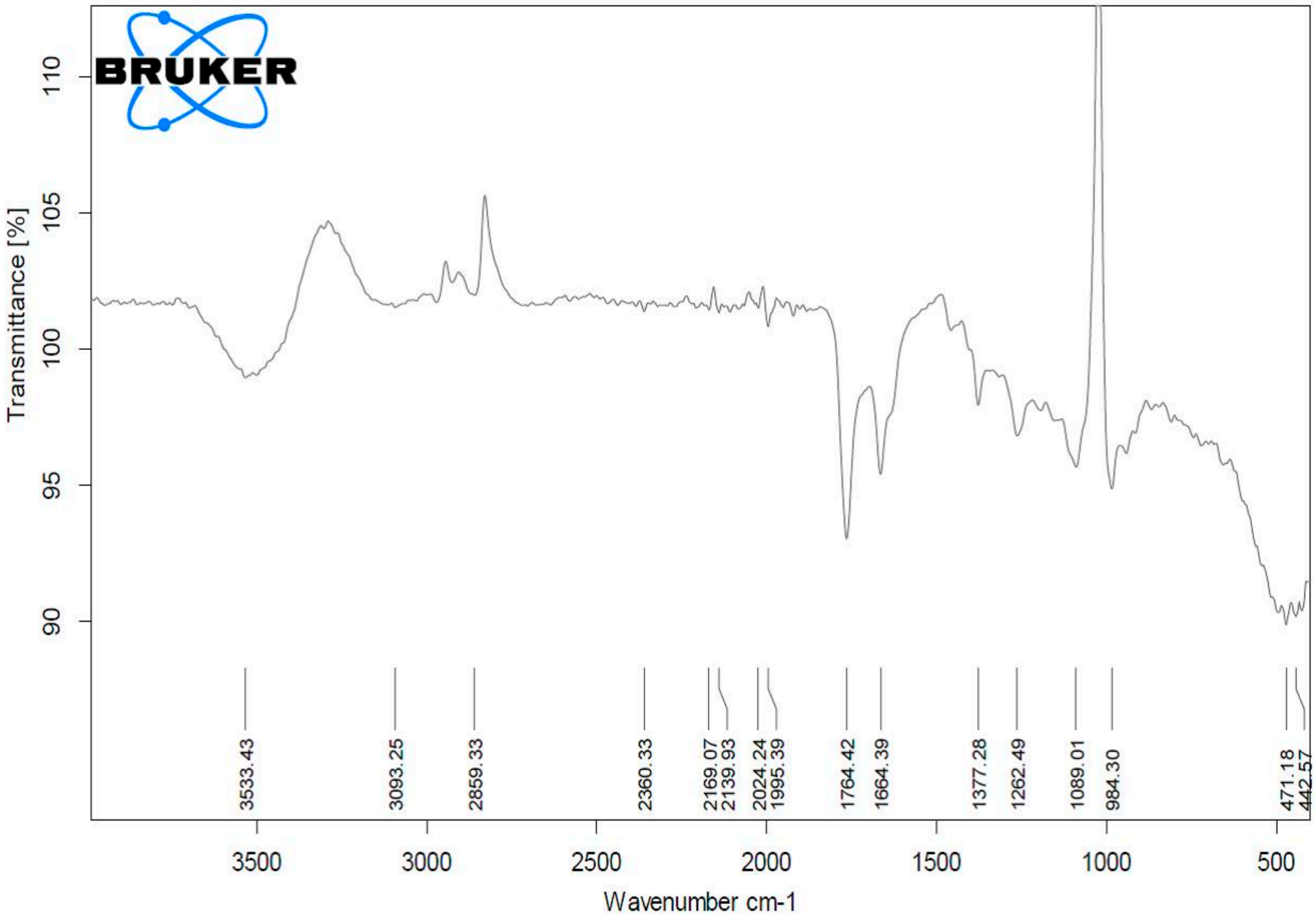

Figure S40. Uprolide N IR spectrum.

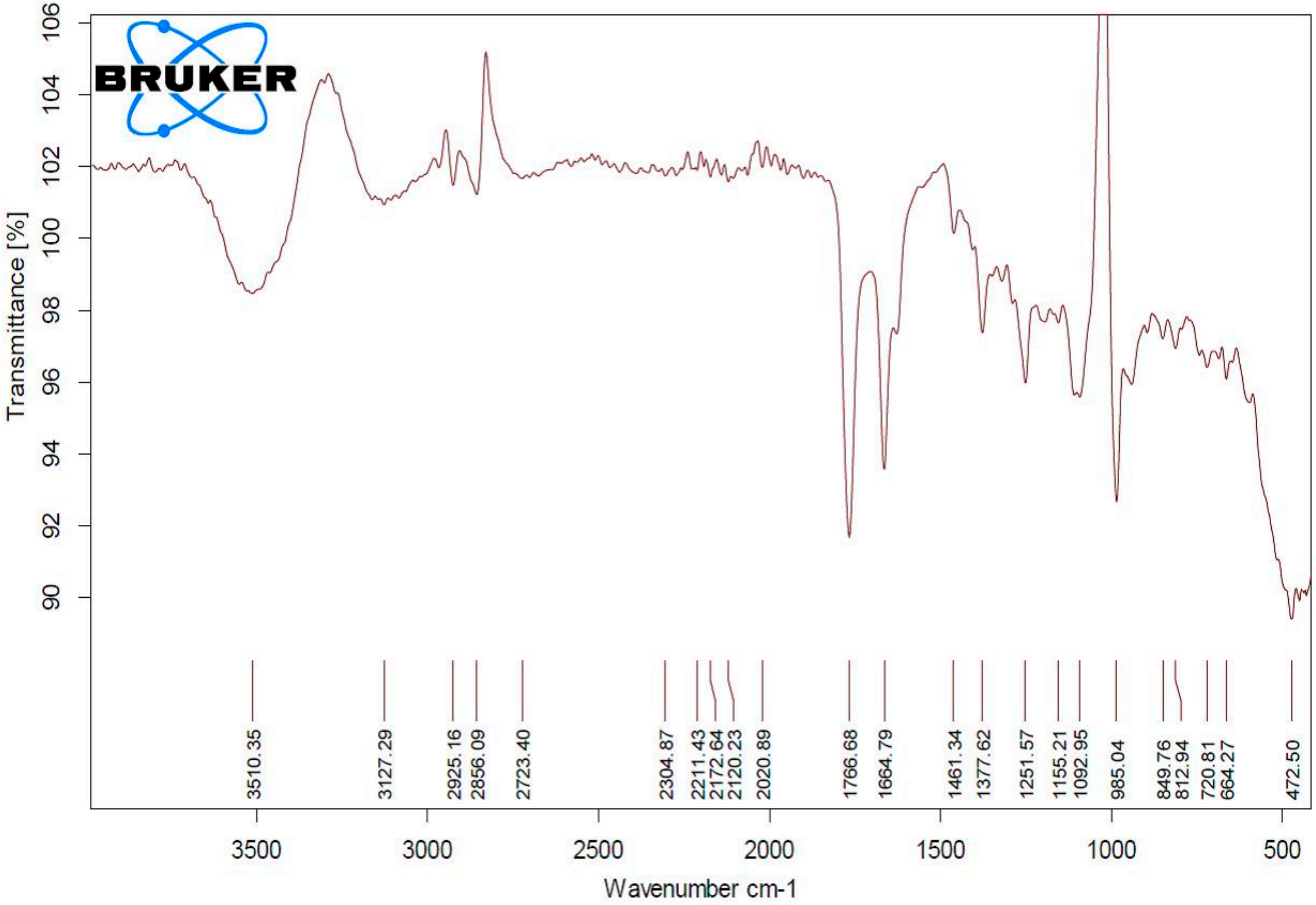

Figure S41. Uprolide O IR spectrum.

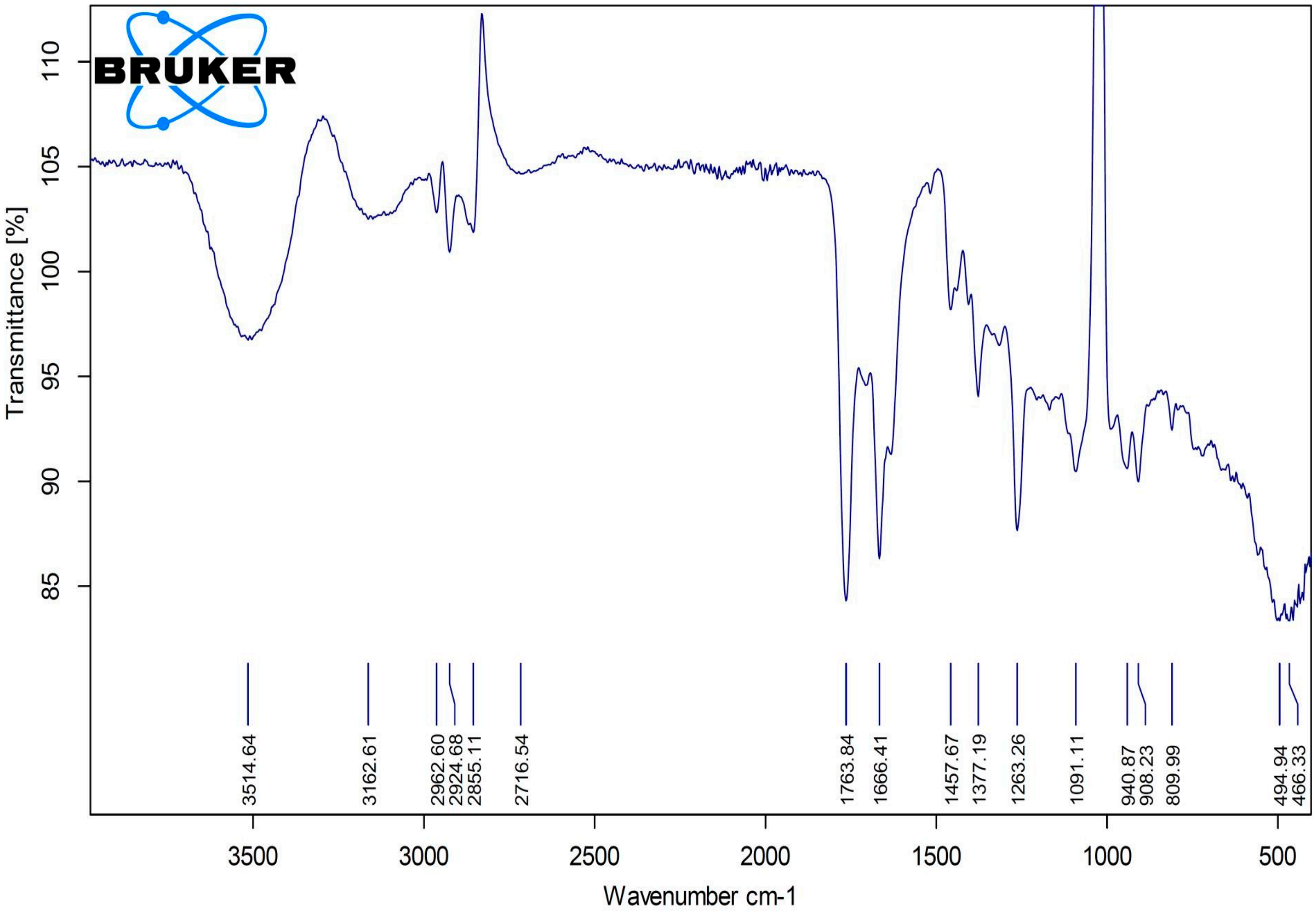

Figure S42. Uprolide P, IR spectrum.

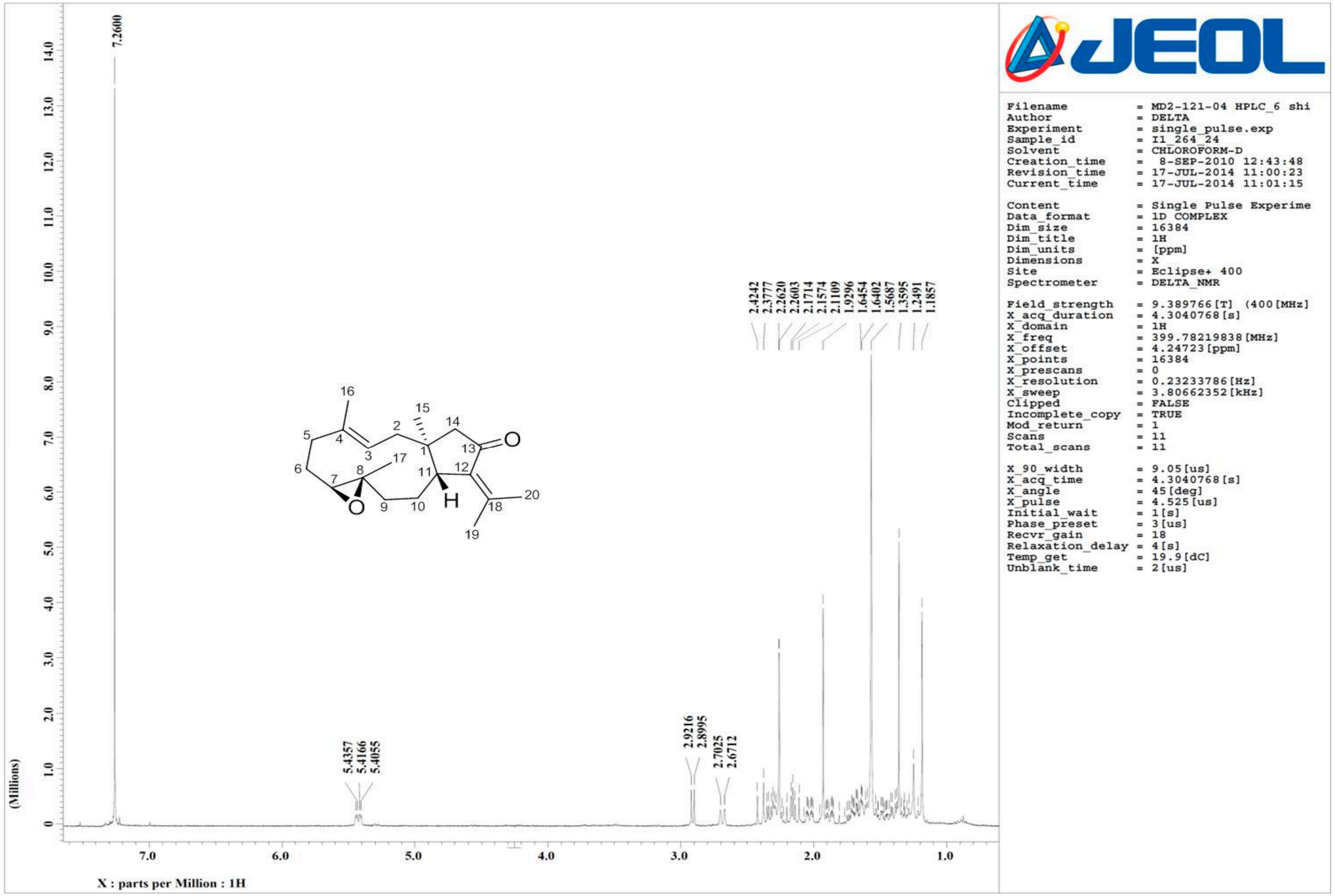

Figure S43. Dolabellane, ^{1}H-NMR spectrum.

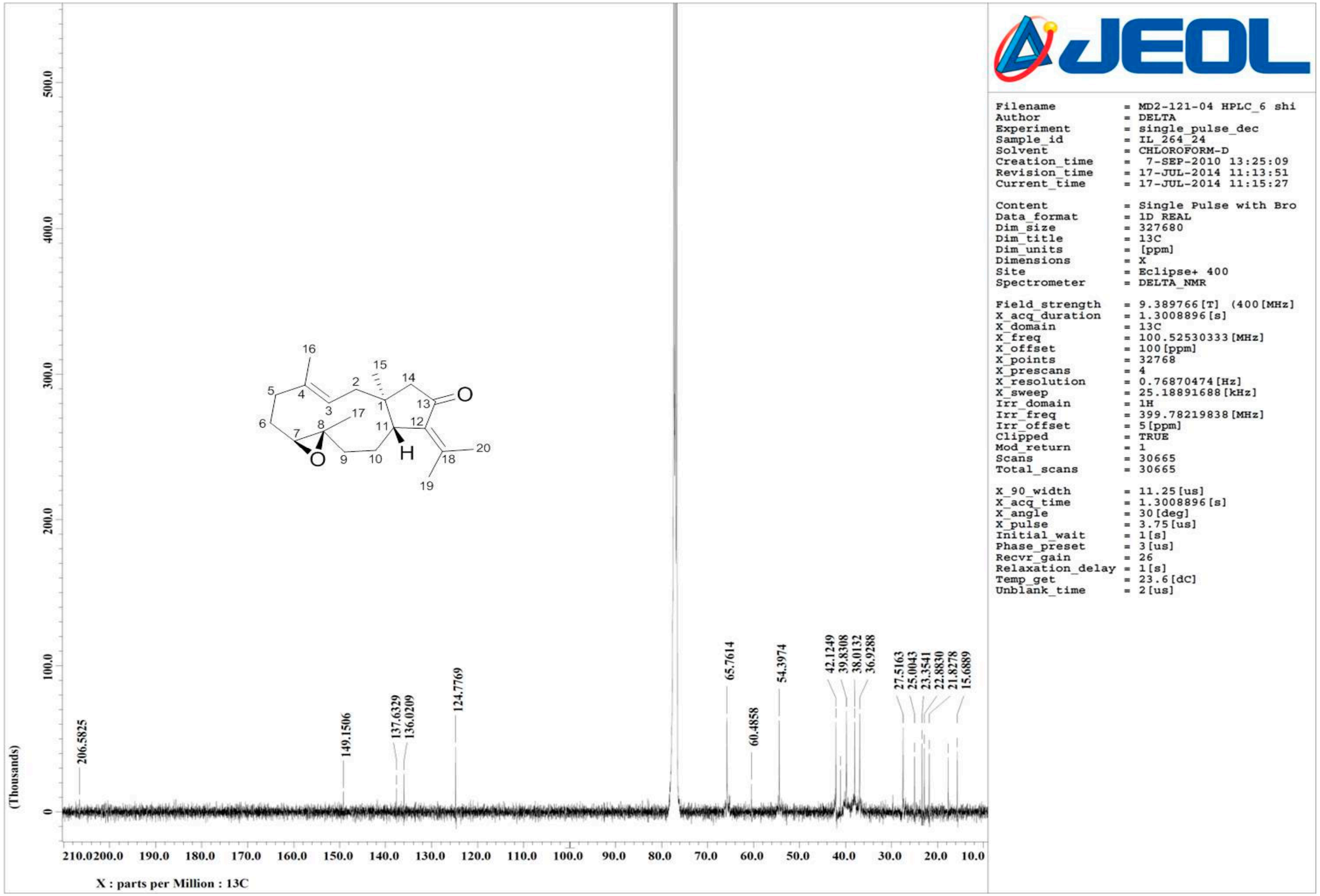

Figure S44. Dolabellane, ^{13}C-NMR spectrum.

File: masa exacta MD2-121-04 HPLC_6Date Run: 09-08-2010 (Time Run: 16:27:29)
Sample: fraccion coral Eunicea masa exacta
Instrument: JEOL LCmate
Inlet: My Inlet Ionization mode: APCI+

Scan: 149 R.T.: 3.72
Base: m/z 303; 23.8%FS TIC: 829328 #Ions: 311

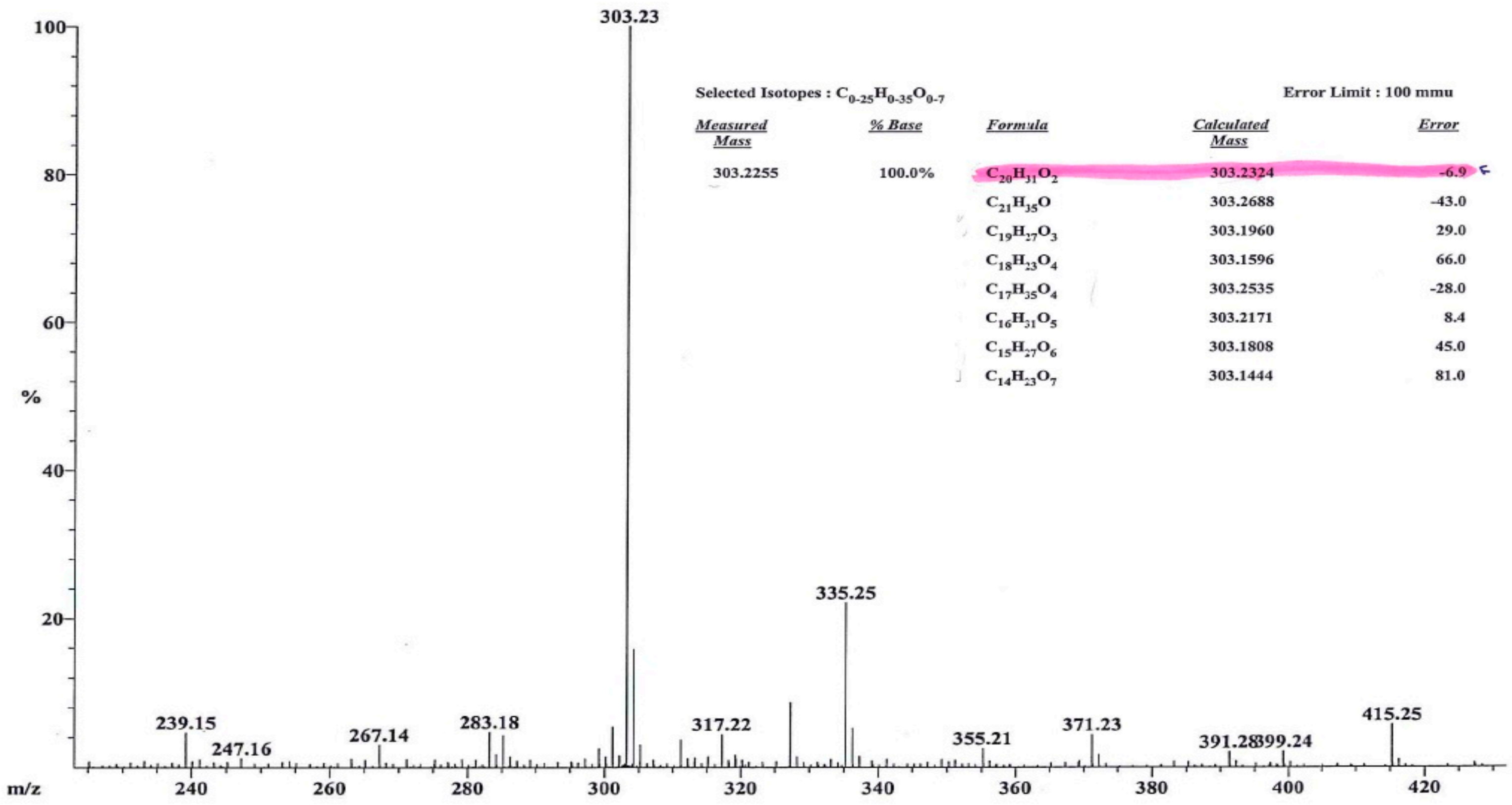

Figure S45. Dolabellane, LRAPCI-MS.

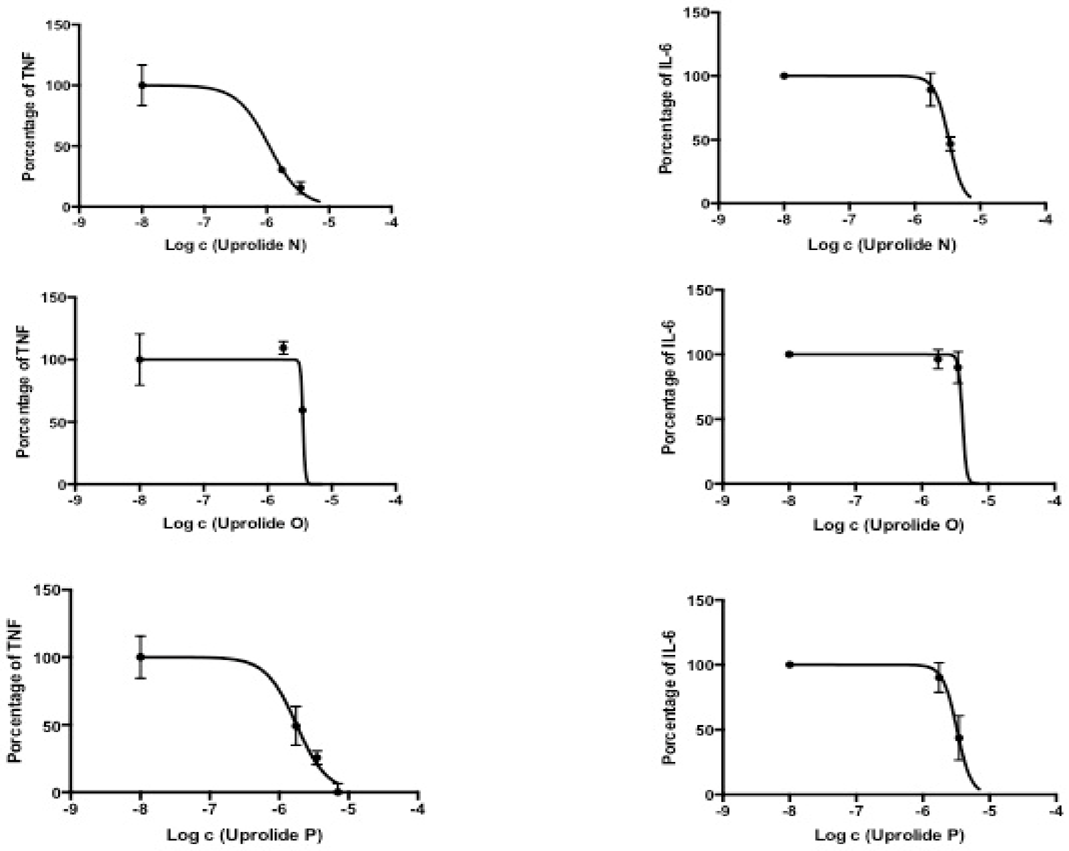

Figure S46. Uprolides N, O and P inhibit the production of inflammatory mediators induced by LPS in murine macrophages. IC_{50} sigmoidal curves calculated by the statistical software package GraphPad Prism 5 from a representative experiments of two Graphs represent the sigmoidal curves for the IC_{50} calculation of TNF (left column) and IL-6 (right column) induced by LPS in the presence of the uprolides N, O or P. Results represent means ± S.D. from stimuli performed in duplicates.

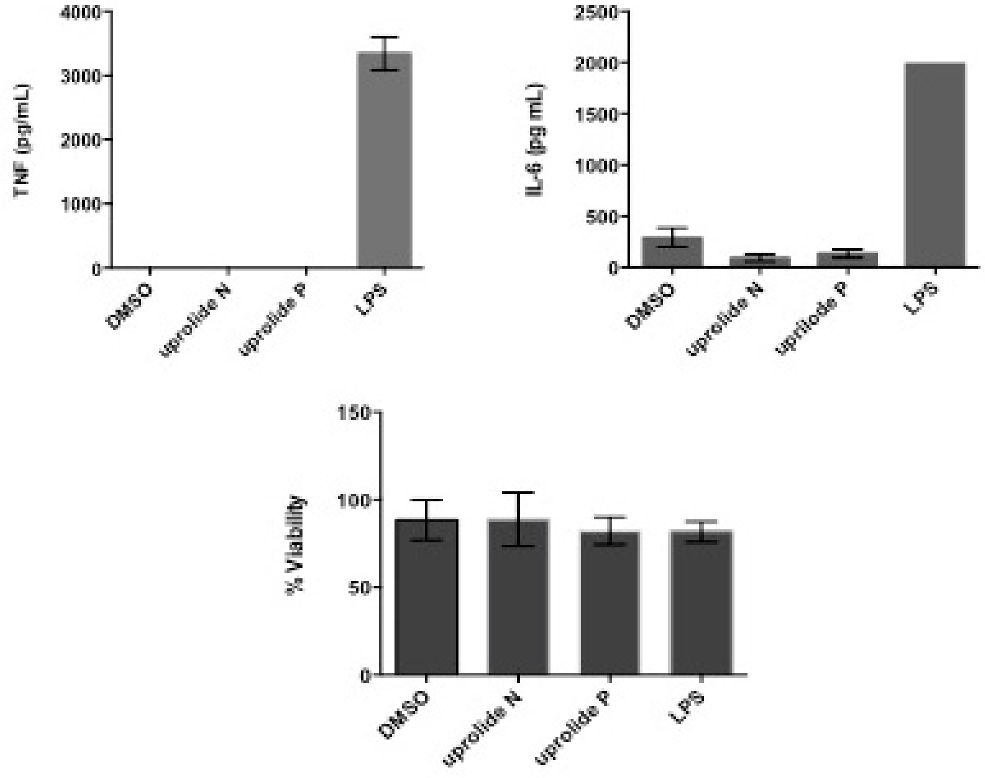

Figure S47. Compound N and P do not induced the production of TNF and IL-6. Macrophages from C57B1/6 mice were treated with 7 μM of compounds **1** or **3** (uprolides N or P) 1 h before stimulation with 100 g/mL of LPS. The supernatants were harvested after 16 h, and cytokine concentrations were determined by ELISA. The cell viability was assessed using MTT assay after collecting the supernatants. Results are represented as Mean ± SD from stimuli performed in duplicate.
